# Novel 1*H*‑1,2,3-Triazole Derivatives of Praziquantel with TRPM_PZQ_ Modulatory Activity and Antiparasitic Effects on Larvae, Juvenile, and Adult Worms of *Schistosoma mansoni*

**DOI:** 10.1021/acsinfecdis.5c00914

**Published:** 2025-12-10

**Authors:** Floriano Paes Silva Junior, Rafael Ferreira Dantas, Sang-Kyu Park, Helen Whiteland, Camilla Thomaz da Silva Oliveira, João M. Rezende-Neto, Jordano Ferreira Reis, Josephine Forde-Thomas, Luciano Pinho Gomes, Walter C. G. Valente, Giuliana Viegas Schirato, Frederico Ricardo de Castro Noronha, Karl F. Hoffmann, Jonathan S. Marchant, Sabrina Baptista Ferreira

**Affiliations:** † Laboratório de Bioquímica Experimental e Computacional de Fármacos, Instituto Oswaldo Cruz, Fiocruz, Rio de Janeiro 21040-900, Brazil; ‡ Department of Cell Biology, Neurobiology & Anatomy, Medical College of Wisconsin, Milwaukee, Wisconsin 53226, United States; § Department of Life Sciences, Aberystwyth University, Edward Llwyd Building, Aberystwyth SY23 3DA, U.K.; ∥ Laboratório de Síntese Orgânica e Prospecção Biológica, Universidade Federal do Rio de Janeiro, Instituto de Química, Departamento de Química Orgânica, Rio de Janeiro 21941-590, Brazil

**Keywords:** schistosomiasis drug discovery, Schistosoma mansoni, praziquantel analogues, triazoles, phenotypic screening, TRPM modulators

## Abstract

The identification of a transient receptor potential ion channel of the melastatin subfamily activated by praziquantel (TRPM_PZQ_) has opened new opportunities for target-based schistosomiasis drug discovery. In this study, eight new 1*H*-1,2,3-triazole derivatives of praziquantel (PZQ), and their synthetic intermediates, were prepared and evaluated for their schistosomicidal activity on schistosomula, juvenile, and adult *Schistosoma mansoni*. Their ability to activate schistosome wild-type (WT) and mutant TRPM_PZQ_ (*Sm*.TRPM_PZQ_), as well as a schistosome TRPM channel activated by meclonazepam (*Sm*.TRPM_MCLZ_), and TRPM_PZQ_ from *Fasciola hepatica* (*Fh*.TRPM_PZQ_) and *Echinococcus granulosus* (*Eg*.TRPM_PZQ_), was also assessed. Initial screening of schistosomula identified six compounds significantly affecting parasite motility/morphology at 25–50 μM. Compounds **3**, **4**, and **5e** were active against juveniles by two orthogonal methods. All compounds impaired adult worm motility, with **4** being the most potent in males (EC_50_: 1.3–2.3 μM) and **5e** being the most potent in females (EC_50_: 3.1–3.9 μM). Compound **5e** showed the highest selectivity indexes (75 for females and 155 for males) when compared with the HepG2 human cell line. Compounds **2**, **3**, **4**, and **5e** activated WT (EC_50_: 0.9–13.5 μM), and mutant *Sm*.TRPM_PZQ_ showing a similar activation profile to PZQ. Like PZQ, they did not activate *Fh*.TRPM_PZQ_ or *Sm*.TRPM_MCLZ_ at the tested concentrations but activated *Eg*.TRPM_PZQ_ with similar potencies to *Sm*.TRPM_PZQ_. Molecular modeling studies suggest that the PZQ binding site on *Sm*.TRPM_PZQ_ may accommodate extended substituents on position 9 of the pyrazinoisoquinoline ring due to a conformational flexibility of the Y1517 side chain. This feature could be explored to design new PZQ analogues with improved drug metabolism and pharmacokinetic properties.

## Graphical abstract

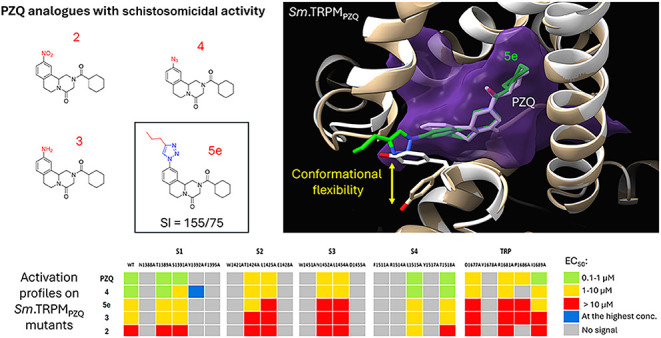

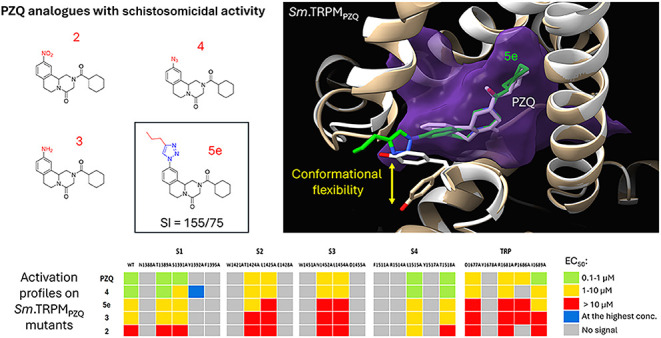

Schistosomiasis (Bilharzia) is a neglected tropical disease caused by infection with trematode blood flukes of the genus *Schistosoma* (e.g., *Schistosoma mansoni*, *Schistosoma japonicum*, *Schistosoma hematobium*). According to the World Health Organization (WHO), approximately 240 million people are affected globally by this disease, and over 700 million live in endemic areas—primarily in low-income communities located in tropical and subtropical regions.^1,2^ It is estimated that schistosomiasis is responsible for a global burden of approximately 3.31 million disability-adjusted life years (DALYs).^3^

Praziquantel (PZQ), commercially known as Biltricide, is a pyrazinoisoquinoline derivative that has been used as the drug of choice for the treatment of schistosomiasis for over four decades. Its anthelmintic activity was identified in the early 1970s as a result of collaborative research between Merck and Bayer AG.^4^ The therapeutic success of PZQ is attributed to several factors, including its efficacy against all *Schistosoma* species, good tolerability, and low price. In addition, PZQ also displays activity against a broad spectrum of other blood, lung, intestinal, and liver flukes, with the exception of *Fasciola hepatica*.^5,6^ These characteristics led the WHO to recommend its use in periodic mass drug administration (MDA) campaigns, which help treat millions of people every year—primarily school-aged children.^5,7^

Despite its success, PZQ has several important limitations. Although with varying determined potencies, *in vitro* studies with cultivated parasites have demonstrated that PZQ is active against schistosomula (1.3–4.8 μM^8–11^ ), juvenile (0.09–0.54 ^12^ and 4.7 ^13^ μM) and adult (0.16–0.75 μM^9,13–15^ ) forms of *S. mansoni*. However, *in vivo* studies using animal models have shown that PZQ is less effective or relatively ineffective against 2–4-week-old juveniles,^16,17^ a developmental stage that may be present during PZQ treatment in humans and potentially contribute to treatment failure.^18,19^ Additionally, PZQ is marketed as a racemic mixture of the R- and S-enantiomers in large bitter-tasting tablets, making it difficult to administer to school-aged children. These issues are largely attributed to the (S)-PZQ isomer, which is poorly water-soluble and pharmacologically inactive.^20^ Recently, an orodispersible formulation of R-PZQ (arpraziquantel, Merck) has been developed and represents a promising alternative to improve treatment acceptability, especially in pediatric populations.^21^ The widespread use of PZQ in MDA also raises concerns regarding the potential emergence of resistant strains, especially given that resistance has already been induced under laboratory conditions.^19^ Together, these limitations highlight the need for novel therapeutic alternatives to treat schistosomiasis.

For many years, the exact mechanism of action of PZQ was unknown. However, in 2019, a study demonstrated that PZQ activates a Ca^2+^-permeable ion channel in *S. mansoni* known as the transient receptor potential channel of the melastatin subfamily (*Sm*.TRPM_PZQ_).^22^ Subsequent studies have shown robust evidence identifying *Sm*.TRPM_PZQ_ as the potential “primary” therapeutic target of PZQ.^23^ Consequently, this channel represents a promising target for the discovery of novel schistosomicidal drugs.^24^ TRPM_PZQ_ orthologs were also found in all available parasitic flatworm genomes, including *F. hepatica* (*Fh*.TRPM_PZQ_) and *Echinococcus granulosus* (*Eg*.TRPM_PZQ_), suggesting that TRPM_PZQ_ modulators may also be applicable to other parasitic diseases.^25^

PZQ is predicted to bind within the voltage-sensor-like domain (VSLD) cavity of *Sm*.TRPM_PZQ_, where it interacts with 23 residues located in the S1–S4 transmembrane helices and the cytosolic TRP box domain.^26^ Sequence analysis of TRPM_PZQ_ channels from different trematodes revealed that these residues are highly conserved, except for *Fasciola spp.*, which possess a threonine at a specific position in the S1 segment (T1270) instead of the asparagine found in other trematodes (e.g., N1388 in *S. mansoni*).^25^ This single amino acid substitution prevents *Fh*.TRPM_PZQ_ from being activated by PZQ and may provide an explanation for the natural insensitivity of *Fasciola spp*. to PZQ treatment.^26^

Over the past 20 years, much has been reported in the literature on the preparation of praziquantel analogues with the aim of circumventing some of its limitations, as well as being prepared for the case of resistance development by the parasites. However, despite these efforts, a significant improvement in antischistosomal activity was never achieved, especially against the juvenile form. There are many reports in the literature of modifications made to the cyclohexane amide portion of PZQ, which, in most analogues, resulted in a total loss of activity. Another type of structural modification of PZQ in the literature is the modification of the substituents of the aromatic ring.^27,28^ Some authors have reported the synthesis of new derivatives of PZQ with modifications in the aromatic ring at different positions, and have identified that position 9 in the ring produces potential new compounds, but all were less efficient than PZQ against adult worms (Figure 1).^29,30^

**Figure 1:**
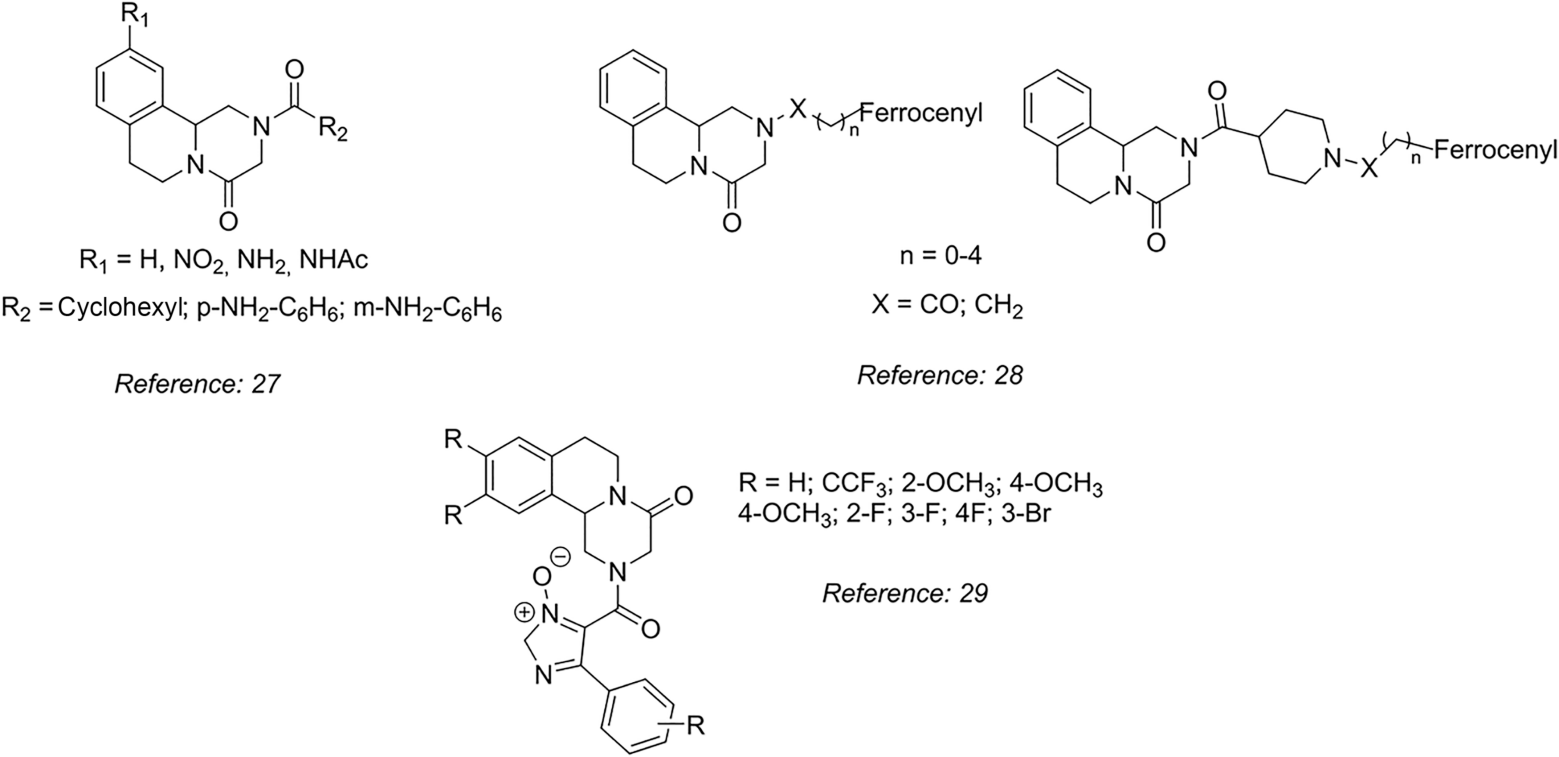
Examples of PZQ derivatives reported in the literature.

Heterocyclic-based azo derivatives are potent pharmacophore groups that can be used for drug development of novel molecules against parasitic diseases, such as schistosomiasis.^31^ Among those, the triazole scaffold has attracted great interest as the basis for the synthesis of various biologically important compounds including derivatives with activity against *S. mansoni*.^32–35^ Triazole has great stability against oxidation reactions and enzymatic hydrolysis processes, and this heterocycle can establish several noncovalent interactions such as hydrogen bonding, π-stacking, and dipole–dipole, which justifies the biological interest in its derivatives. Furthermore, unlike with other aza-heterocycles, due to its weak basicity, the 1,2,3-triazole ring is not protonated at physiological pH.^36^

In this study, we synthesized a novel series of PZQ analogues containing a 1,4-disubstituted 1,2,3-triazole nucleus attached to position 9 of the aromatic ring of PZQ. These triazoles, along with their synthetic intermediates, were subsequently evaluated in phenotypic assays to assess their schistosomicidal activity against schistosomula, juvenile, and adult stages of *S. mansoni*. Their cytotoxicity and selectivity were also determined using human cell lines. Finally, the ability of the PZQ analogues to activate both wild-type and mutant versions of *Sm*.TRPM_PZQ_ was investigated to elucidate whether they share a similar mechanism of action and binding mode with PZQ. Their activity was also assessed on three additional TRPM channels, *Fh*.TRPM_PZQ_, *Eg*.TRPM_PZQ_, and *Sm*.TRPM_MCLZ_ (a channel implicated in mediating the toxic effects of meclonazepam (MCLZ) on juvenile schistosomes^37^) to evaluate selectivity and investigate whether these new analogues could potentially broaden the therapeutic scope of PZQ.

## Results and Discussion

### Chemistry

A synthetic methodology was developed starting from commercially available (R/S)-PZQ **1**, as outlined in Scheme 1. The ^1^H NMR (300 MHz, CDCl_3_) and ^13^C NMR (75 MHz, CDCl_3_) spectra for all synthesized compounds and intermediates, along with COSY (500 MHz, CDCl_3_), HSQC (500 MHz, CDCl_3_), HMBC (500 MHz, CDCl_3_), and NOESY (500 MHz, CDCl_3_) spectra for compound **2** can be found in Supporting Information (Figures S1–S26). This route comprises a sequence of reactions designed to obtain the key azide intermediate **4**, which was subsequently employed in the synthesis of target compounds **5a**–**h**. The synthesis of the 1,2,3-triazole derivatives started with the nitration of praziquantel **1**. The preparation of the 9-NO_2_–PZQ (**2**) derivative was carried out using a nitration methodology widely reported in the literature involving the *in situ* generation of the nitronium ion via a mixture of sulfuric and nitric acids.^27^ Modification of reaction temperature to −5 °C for 4 h led to a higher yield of the desired nitrated product. Following the synthesis of nitrated compound **2**, the reduction of the nitro group was carried out. The methodology employed was the catalytic hydrogenation^38^ of compound **2** to obtain the corresponding amino derivative 9-NH_2_–PZQ (**3**). The key intermediate azide **4** was synthesized in two steps: diazotization of the aromatic amine group, followed by substitution with sodium azide. Finally, the synthesis of 1,4-disubstituted 1,2,3-triazoles was carried out using the 1,3-dipolar cycloaddition methodology, commonly referred to as the Huisgen reaction, which has been extensively employed.^39–41^ This approach involves the copper­(I)-catalyzed reaction between an azide group and a terminal alkyne. Thus, the PZQ-triazoles were obtained by reacting the azide derivative **4** with different terminal alkynes a-h through microwave irradiation, in the presence of copper­(II) sulfate pentahydrate and sodium ascorbate, using H_2_O and tert-butanol under magnetic stirring (600 rpm) for a period ranging from 10 to 15 min, as monitored by thin-layer chromatography. After isolation, in some cases, purification by column chromatography was required due to the presence of residual reagents, yielding the products **5a**–**h** with yields ranging from 42 to 99%. The 1,2,3-triazole derivatives **5a–h**, along with their respective intermediates, were characterized by ^1^H and ^13^C NMR and infrared (IR) spectroscopy.

**Scheme 1:**
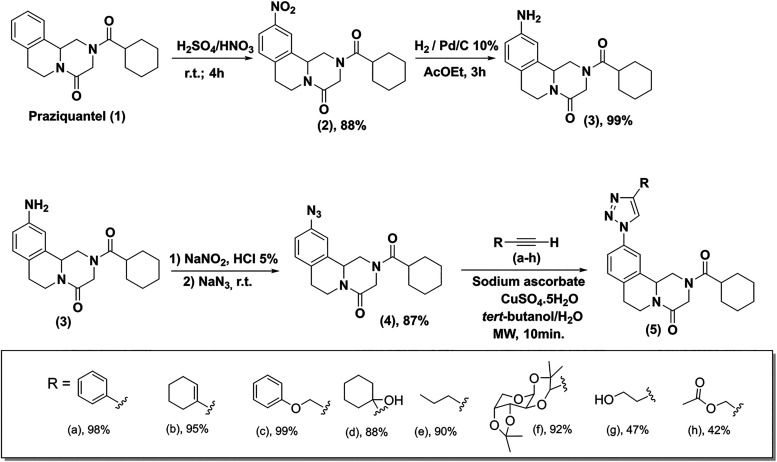
Synthesis of 1*H*-1,2,3-Triazoles Derivatives of (R/S)-PZQ

### New PZQ Derivatives Antischistosomal Activity Profile and *In Vitro* Selectivity

#### Schistosomula

All PZQ derivatives were synthesized in the racemic form and evaluated as such. The compounds were initially screened at 10 μM against *S. mansoni* schistosomula using the Roboworm high-content screening assay^42,43^ and were declared hits or nonhits based on their effect on parasites’ motility and phenotype scores after 72 h of incubation. As none of the compounds were considered hits at this concentration, we performed a titration from 6.25 to 50 μM (Figure S27). Notably, PZQ was not classified as a hit at the lowest concentration tested (6.25 μM) and other derivatives were borderline at concentrations below 50 μM, such as **4** at 12.5 μM and **5c** at 25 μM. Except for compound **4** (declared a hit at 25 μM), all other PZQ derivatives that were declared hits were effective only at 50 μM (Figure 2).

**Figure 2:**
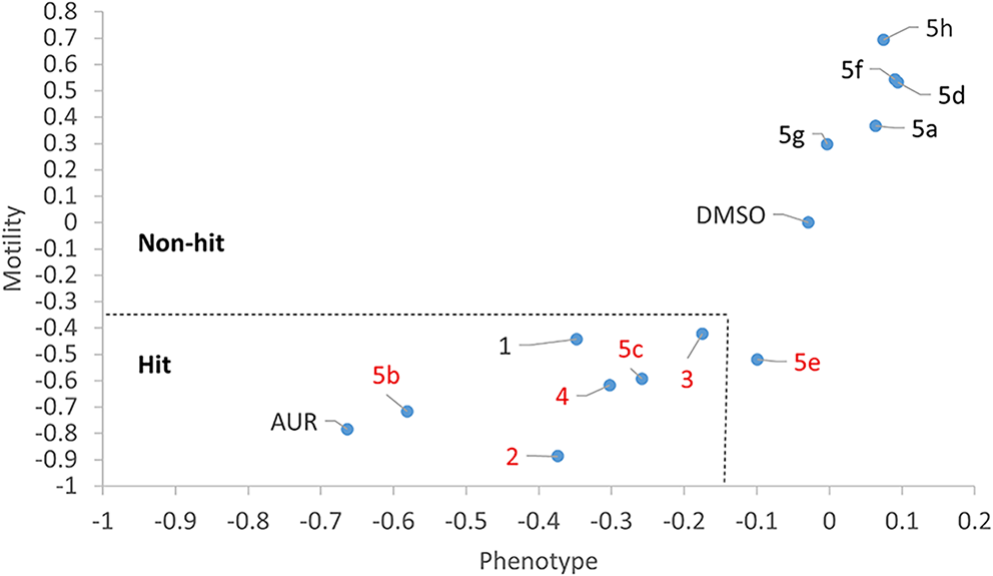
Screening of PZQ analogues against *S. mansoni* schistosomula at 50 μM after 72h of incubation using the Roboworm high-content screening assay. PZQ (**1**) and auranofin (AUR) were both included (at 10 μM) as positive controls. DMSO was included as a negative control. Compounds were considered hits if both their motility and phenotype scores were lower than −0.35 and −0.15, respectively (region delimited by the dashed lines). In red, PZQ analogues selected for further analysis.

In total, six out of 11 tested compounds were declared hits at 50 μM: two triazoles with bulk cyclohexenyl or phenoxymethyl substituents (**5b** and **5c**, respectively) and the three synthetic intermediates (**2**, **3**, and **4**). Another triazole, **5e**, which carries a shorter n-propyl substituent, also showed a significant effect on parasites’ motility, but did not change their phenotype score enough to be considered a hit (Figure 2). Nonetheless, it was selected for further analysis along with the hit compounds.

#### Juveniles

The low sensitivity of schistosome juvenile worms (21 days p.i.) to PZQ is considered the Achilles heel of current antischistosomal chemotherapy, given the potential to reduce treatment efficacy, especially in mass drug administration scenarios.^19^ Therefore, in view of the key importance of this lifecycle stage, all compounds that were active against schistosomula were subsequently evaluated in juveniles by two assay methods: a colorimetric assay based on the metabolic reduction of XTT, and a microscopy analysis that assigns a viability score based on visual alterations in parasite morphology and motility. By combining two orthogonal assay methods, confidence in the efficacy evaluation of the compounds can be increased. We first screened compounds at high concentration (100 μM) and shorter incubation time (48h) using the XTT assay to determine if there was any activity for a given compound before proceeding to the more time-consuming microscopy method (Figure S28).

Only PZQ (**1**) and its analogues **3**, **4**, and **5e** significantly decreased parasite metabolic activity after 48h incubation at 100 μM compared to the DMSO control group. Despite **4** having reduced juvenile worm’ viability to 44.6% ± 13.3 compared to 71.5% ± 12.6 by PZQ, one-way ANOVA followed by Dunnett’s multiple comparison test indicated none of the analogues impaired juvenile worm viability differently from PZQ (Figure S28). These results indicate that some PZQ analogues may exert an effect on the metabolism of juvenile schistosomes similar to that of PZQ at this concentration and incubation time.

For microscopy analysis, juvenile schistosomes treated with DMSO, PZQ, or its synthetic derivatives were assigned viability scores ranging from 0 to 3, with 3 representing a normal phenotype and 0 corresponding to extreme damage or death. This analysis allowed the generation of a phenotypic profile for each treatment group, which was then compared with that of the DMSO control (Figure 3 and Table S1).

**Figure 3:**
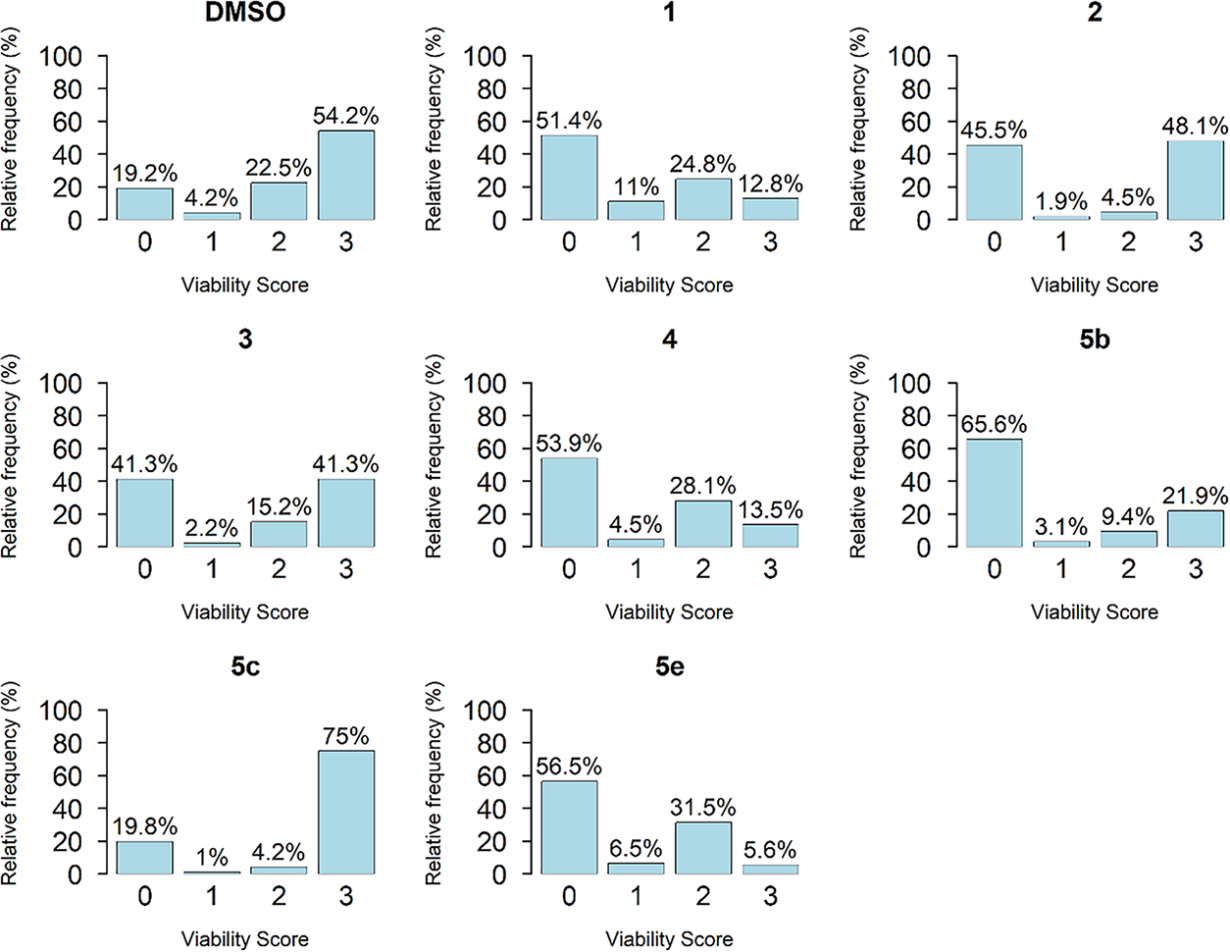
–Microscopy analysis of juvenile schistosomes incubated with 10 μM of PZQ (**1**) and its derivatives or 0.02% DMSO for 72 h. The bars represent the relative frequencies for each viability score.

The phenotypic profiles observed after incubating parasites with 10 μM PZQ or its derivatives for 72 h were significantly different from those observed with DMSO treatment, according to Pearson’s Chi-squared test (Table S2). These results confirmed the metabolic assessment by the XTT method, where PZQ, **3**, **4**, and **5e** induced phenotypes associated with increased frequency of dead or damaged worms or reduced frequency of healthy worms. Notably, **5e** treatment also displayed a tendency for increased deleterious effects compared to PZQ (e.g., reduction in frequency of healthy juveniles to 5.6% compared to 12.8%); however, no statistically significant difference was observed (Pearson’s Chi-squared test) (Table S3).

On the other hand, **5c** showed a higher relative frequency of normal phenotypes (i.e., viability score 3) compared to DMSO-treated worms (Figure 3). This unexpected result may be attributed to inherent difficulties in visual analysis, particularly in identifying more subtle alterations in parasites, which can sometimes lead to misclassification. To test this hypothesis, we grouped scores 2 and 3, as well as scores 0 and 1, and analyzed the resulting relative frequencies (Figure S29). The results indicate that the phenotypic profiles of DMSO- and **5c**-treated parasites are similar (no statistical difference by the Chi-squared homogeneity test), suggesting that this compound lacks activity against juvenile schistosomes under the conditions tested. This conclusion is further supported by the XTT assay, which also showed no statistically significant differences between the two groups. Distinct from PZQ analogue **5e**, compound **5c** carries a bulkier phenoxymethyl substituent on position 4 of the triazole ring, which indicates that the size of the R group may be key to schistosomicidal activity.

#### Adults

The PZQ derivatives selected after the initial screening on schistosomula were also tested at concentrations ranging from 0.01 to 100 μM on adult schistosomes using our automated image-based motility assay.^44,45^ The EC_50_ values, derived from an analysis of the dose–response curves, are presented in Table 1.

**Table 1:** Male and Female Worms’ Motility Inhibition by PZQ Analogues

Cpd/Time	Male worm motility inhibition (EC_50_; μM)				Female worm motility inhibition (EC_50_; μM)			
	0 h^a^	24 h	48 h	72 h	0 h^a^	24 h	48 h	72 h
±PZQ (**1**)	0.28	0.25	0.25	0.16	ND	ND	ND	ND
**2**	11	14	16	15	27.7	29	29	30
**3**	UD	5.4	4.5	3.5	8.36	9.6	9.9	11
**4**	2.3	1.8	1.6	1.3	6.7	3.1	9.4	3.5
**5b**	33	47	49	40	101	96	95	89
**5c**	NI	PF (>100)	PF (>100)	PF (>100)	ND	ND	ND	ND
**5e**	2.6	2.5	1.9	1.7	3.5	3.6	3.9	3.1

a Immediately after the addition of compounds. The EC_50_ values represent either a single experiment or the geometric mean of the values calculated from independent experiments (see Table S3). Dose–response curves are shown in Figures S30–S36. For some compounds, EC_50_ values could not be calculated either because the experiment was not performed (ND: not determined), the concentrations tested were insufficient to reach the bottom plateau of the dose–response curve (partial fit: PF), or the curve fitting algorithm failed (UD: undefined). NI: no inhibition.

The EC_50_ values obtained for PZQ analogues ranged from approximately 1.3–49 μM for male worms and 3.1–101 μM for female worms (Table 1). These values were at least ten times higher than those obtained for PZQ (∼0.2 μM) in male worms, suggesting that the chemical modifications introduced into the PZQ molecule, yet tolerated, mostly negatively impacted its antischistosomal activity *in vitro*. Overall, the EC_50_ values remained largely unchanged over time, indicating that like PZQ, the effect on the adult worm musculature occurs almost immediately upon incubation with the tested compounds. Among the PZQ analogues, intermediate **4**, carrying an azide group on position 9, had the highest potency against male worms (1.3–2.3 μM), and triazole **5e** was the most potent against female worms (3.1–3.9 μM), while compound **5b** exhibited the lowest potency (>30 μM) in both sexes. Once again, a triazole bearing an R group bulkier than the n-propyl substituent had diminished potency activity on *S. mansoni*.

#### Cytotoxicity

Alongside the studies of schistosomes, the PZQ analogues were also tested on human cell lines (WSS-1 and HepG2) to measure overt cytotoxicity. An initial screening at 10 μM against WSS-1 kidney epithelial cells revealed that none of the compounds significantly decreased cell viability after 48 h of incubation (Figure S37). Next, we conducted a more detailed investigation by performing dose–response assays with each compound on HepG2 hepatic epithelial cells, as the liver is one of the major organs affected in intestinal schistosomiasis. From this analysis, the half-maximum cytotoxic concentration (CC_50_) and selectivity index (SI), relative to adult worms, were calculated after 48 h of incubation (Table 2 and Figure S38).

**Table 2:** Cytotoxicity and Selectivity of PZQ Analogues after 48 h of Incubation

		Worm motility inhibition (EC_50_; μM)		Selectivity Index (CC_50_/EC_50_)	
Compound	HepG2 cytotoxicity (CC_50_; μM)	Male	Female	Male	Female
±PZQ (**1**)	>300	0.25	ND	>1200	ND
2	368	16	29	23	13
3	>300	4.5	9.9	>67	>30
4	156	1.6	9.4	98	17
5b	28	49	95	0.6	0.3
5c	88	PF (>100)	ND	<0.9	ND
5e	294	1.9	3.9	155	75

The EC_50_ values represent either a single experiment or the geometric mean of the values calculated from independent experiments (see Table S4). Dose–response curves are shown in Figures S30–36 and S38. For some compounds, EC_50_ values could not be calculated either because the experiment was not performed (ND: not determined) or the concentrations tested were insufficient to reach the bottom plateau of the dose–response curve (PF: partial fit).

According to Table 2, PZQ and three of its analogues (**2**, **3**, and **5e**) exhibited CC_50_ values near or above 300 μM. The precise CC_50_ values for PZQ and **3** could not be determined, as the higher concentrations required to fit the dose response curves would exceed the maximum concentration of DMSO tolerated by HepG2 cells.

PZQ showed the highest SI (>1200), being over a thousand times more active against schistosomes than HepG2 cells (Table 2). This result was expected, as PZQ is known for its high tolerability. Apart from **3**, whose CC_50_ was inconclusive, and **5c**, for which the EC_50_ was inconclusive (male) or not determined (female), **5e** was the most selective PZQ analogue, with an SI of 155 for male and 75 for female worms compared to human cells. Although these SI values are not as high as those observed for PZQ, **5e** remains a promising candidate for structural optimization due to its high activity against schistosomes (EC_50_ = 1.9–3.9 μM) and good selectivity (SI > 100). In contrast, compounds **5b** and **5c** were more potent on HepG2 cells than on parasites (SI < 1), suggesting that the chemical modifications in these compounds significantly enhanced PZQ’s cytotoxicity.

#### New PZQ Analogues Act as *Sm*.TRPM_PZQ_ Agonists

To determine whether our PZQ analogues share the same mechanism of action as PZQ, we performed Ca^2+^ reporter activation assay profiling of *Sm*.TRPM_PZQ_. Compounds were tested at various concentrations (0.001 to 100 μM), and EC_50_ values for channel activation were determined. These data are summarized in Table 3 and Figure 4.

**Figure 4:**
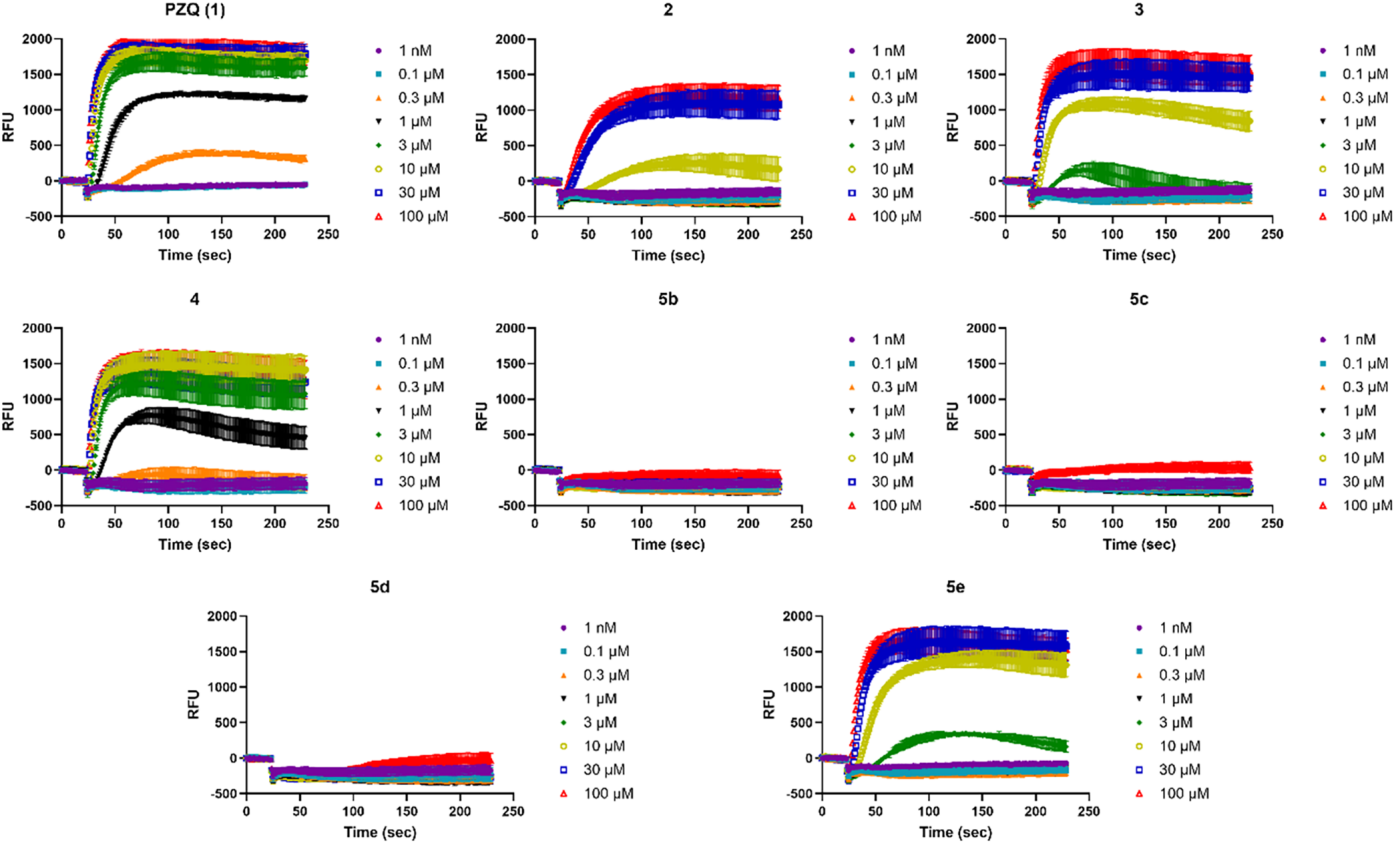
–*Sm*.TRPM_PZQ_ activation by PZQ and its analogues tested at various concentrations. Channel activation is represented by an increase in relative fluorescence unit (RFU) values over time.

**Table 3:** EC_50_ Values for Wild-Type *Sm*.TRPM_PZQ_ Activation by PZQ Derivatives

Compound	EC_50_ (μM)	Bmax (%)
±PZQ (**1**)	0.50 ± 0.04	100
**2**	13.53 ± 0.49	66.62 ± 1.98
**3**	7.09 ± 0.18	90.04 ± 1.31
**4**	0.89 ± 0.03	80.53 ± 2.19
**5b**	No activity	No activity
**5c**	No activity	No activity
**5d**	No activity	No activity
**5e**	4.23 ± 0.15	90.25 ± 1.53

Four analogues were observed to activate *Sm*.TRPM_PZQ_ (Table 3). The most potent analogue was **4** (EC_50_ = 0.89 ± 0.03 μM) followed by **5e** (EC_50_ = 4.23 ± 0.15 μM), **3** (EC_50_ = 7.09 ± 0.18 μM), and **2** (EC_50_ = 13.53 ± 0.49 μM). In contrast, **5b**, **5c**, and **5d** failed to activate *Sm*.TRPM_PZQ_, even at the highest concentrations tested (Figure 4). **5d** was previously found to be inactive against both schistosomula (Figure 2) and adult worms (data not shown). **5b** and **5c** were, however, active against larvae (Figure 2) and adult worms (Table 1), suggesting an action independent from *Sm*.TRPM_PZQ_. We note that both of these compounds have a pronounced effect on HepG2 cell viability (Table 2). This activity profile suggests that the *Sm*.TRPM_PZQ_ activation closely matches the antischistosomal activity of the compounds throughout the three evolutive stages evaluated in the present study.

The three inactive analogues were then screened in antagonist mode to assess any ability to block PZQ activation of *Sm*.TRPM_PZQ_. Preincubation of cells with **5b**, **5c**, or **5d** (1 nM-100 μM final concentration for 10 min) failed to impact subsequent responses to PZQ (data not shown). These data suggest that the PZQ binding site can accommodate additions to the 9 position of the pyrazinoisoquinoline ring system, but up to a size similar to that of an n-propyl group.

Finally, selectivity for *Sm*.TRPM_PZQ_ was assessed by profiling responses on the *F. hepatica* (*Fh*.TRPM_PZQ_)^26^ and *E. granulosus* (*Eg*.TRPM_PZQ_)^46^ TRPM_PZQ_ orthologs, as well as the schistosome TRPM paralog *Sm*.TRPM_MCLZ_, a distinct TRPM channel activated by meclonazepam.^37^ Meclonazepam is an anthelmintic benzodiazepine known for its toxicity to juvenile schistosome worms. PZQ does not activate either *Fh*.TRPM_PZQ_, or *Sm*.TRPM_MCLZ_. In the case of *Fh*.TRPM_PZQ_, this lack of activity has been attributed to a single amino acid substitution in the predicted PZQ binding pocket: instead of an asparagine in the S1 domain, as observed in *Sm*.TRPM_PZQ_ (N1388), *Fh*.TRPM_PZQ_ contains a threonine (T1270). The interaction of PZQ with N1388 is essential to *Sm*.TRPM_PZQ_ activation, providing a plausible explanation for why *F. hepatica* does not respond to this drug.^26^ Consequently, the development of PZQ analogues capable of activating either channels could provide a strategy to either broaden the therapeutic scope of PZQ and/or help circumvent any emerging TRPM_PZQ_-based resistance to this drug.^25,26^

Unfortunately, none of the PZQ analogues activated *Fh*.TRPM_PZQ_ or *Sm*.TRPM_MCLZ_ in our assays (data not shown). Given the high structural similarity of our compounds to PZQ, one possible explanation for the lack of *Fh.*TRPM_PZQ_ activation is that, like PZQ, our PZQ analogues also contain a carbonyl group attached to the pyrazin-2-one ring through an amide bond, which is proposed to form a critical hydrogen bonding interaction with the side chain of asparagine (N1388) in the S1 domain.^26^ Therefore, the channel-modulatory activity of these analogues may also depend on interaction with the asparagine residue, which is replaced with threonine (T1270) in *Fh*.TRPM_PZQ_.

Except for **5b** and **5c**, all of the other PZQ derivatives activated *Eg*.TRPM_PZQ_ (Table 4). The most potent analogue was compound **4**, which had an EC_50_ of 0.21 μM—a value only 2.3 times higher than that estimated for PZQ (EC_50_ = 0.09 μM)—suggesting an equivalent potency. Interestingly, compound **5d**, which was not effective on *Sm.*TRPM_PZQ_, was active on *Eg*.TRPM_PZQ_ (EC_50_ = 30.06 μM). This differential response has already been reported for other cyclohexyl-modified and thioamide analogues of PZQ, indicating a broader tolerance of the *Eg*.TRPM_PZQ_ binding pocket to these compounds compared to *Sm*.TRPM_PZQ_.^46^

**Table 4:** EC_50_ Values for *Eg*.TRPM_PZQ_ Activation by PZQ Derivatives

Compound	EC_50_ (μM)^a^	Bmax (%)^a^
±PZQ (**1**)	0.09 ± 0.02	133.08 ± 16.01
**2**	3.72 ± 0.39	113.58 ± 10.47
**3**	1.60 ± 0.30	137.79 ± 13.18
**4**	0.21 ± 0.06	144.95 ± 14.56
**5b**	No signal	10.42 ± 0.31
**5c**	No signal	7.63 ± 0.72
**5d**	30.06 ± 0.90	94.0 ± 4.08
**5e**	1.49 ± 0.28	131.54 ± 15.01

a Values expressed as mean ± standard deviation.

#### Interaction of PZQ Derivatives with *Sm*.TRPM_PZQ_

In order to gain insights into the interaction of these PZQ derivatives with *Sm*.TRPM_PZQ_, we evaluated the activity of each analogue at discrete *Sm*.TRPM_PZQ_ point mutants previously mapped in close proximity to the predicted binding pose of PZQ.^26^ Overall, each PZQ derivative exhibited a response pattern similar to that of PZQ in this panel of *Sm*.TRPM_PZQ_ mutants, notwithstanding their different potencies at the wild-type channel (Figure 5). This trend likely indicates a similar binding mode.

**Figure 5:**
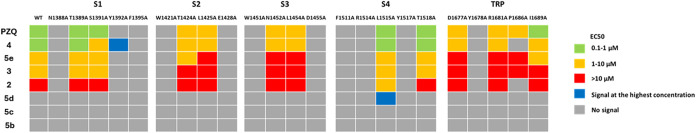
Activation profiles of PZQ and PZQ analogues on wild-type and mutant forms of *Sm*.TRPM_PZQ_. Each column corresponds to either the wild-type or a point-mutated form of the channel at one of 23 residues located in the S1–S4 helices and TRP box domain, which together constitute the predicted binding site for PZQ. The colored cells represent the range of EC_50_ values for each compound based on their ability to activate the channel.

To better understand the binding modes of the active PZQ analogues to *Sm*.TRPM_PZQ_, we performed molecular modeling to compare binding poses of the R-enantiomers with those of R-PZQ (Figures 6 and S39).

**Figure 6:**
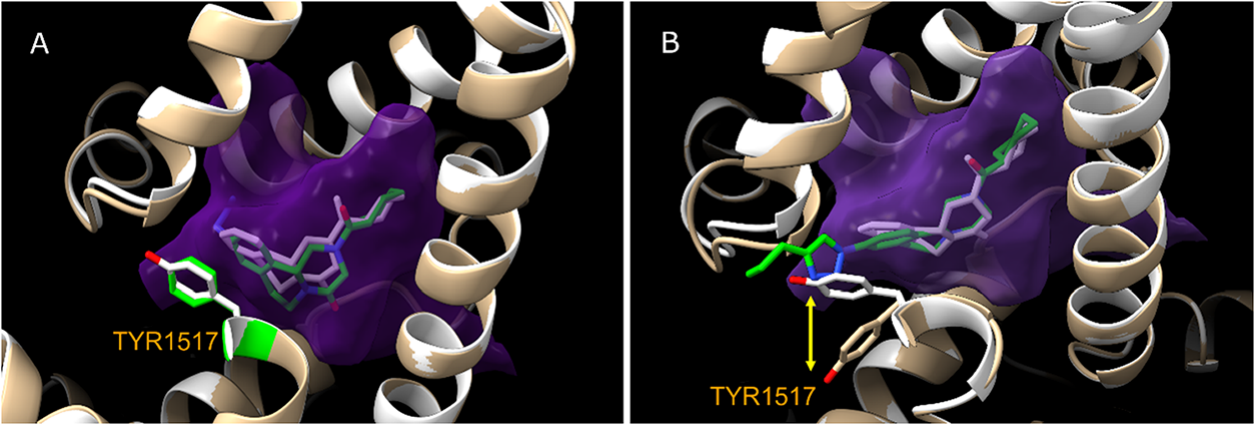
Comparison of the binding poses of R-PZQ and its channel-active analogues **4** (A) and **5e** (B) within the predicted PZQ binding site of the wild-type *Sm*.TRPM_PZQ_. Protein helices were colored white and copper to illustrate the binding poses of PZQ and its analogues, respectively. Compounds are represented as white (PZQ) or green (PZQ analogues) sticks. The cavity surface of the PZQ binding site is colored purple. The yellow double-headed arrow in (B) indicates the conformational change of the Y1517 side chain in the presence of **5e**, compared to its location when bound to PZQ.

Compound **4** showed the highest profile similarity to PZQ. Interestingly, unlike PZQ, it also retained residual activity at 100 μM in *Sm*.TRPM_PZQ_ carrying the Y1392A mutation. In contrast, **5d** activated a mutant form (L1515A) of the channel at 100 μM, suggesting that the isobutyl side chain of L1515 may cause steric hindrance that prevents **5d** from activating the wild-type *Sm*.TRPM_PZQ_. However, this hypothesis is not supported by our computational model as the L1515 side chain is not in contact distance to the substituted triazole moiety in **5d** (Figure S40). An alternative hypothesis is that the leucine-to-alanine substitution in the L1515A mutants may induce a conformational change in the **5d** binding region, thereby increasing its affinity for *Sm*.TRPM_PZQ_.

The predicted binding poses of most of the PZQ analogues are unsurprisingly similar to that of PZQ. However, the triazole compound **5e** induced a prominent conformational shift of the phenol group of Y1517 (Figure 6B), a change not observed in the binding poses of **4** (Figure 6A) and other nontriazole analogues (Figure S39). On the other hand, other triazoles, such as **5b**, **5c**, and **5d**, with R groups bulkier than the n-propyl substituent found in **5e**, would have these groups extending into the space occupied by the phospholipid bilayer of the cell membrane, where the PZQ binding site in *Sm*.TRPM_PZQ_ is predicted to be found.^47^ Therefore, the molecular models suggest a conformational plasticity of the PZQ binding site in *Sm*.TRPM_PZQ_ that could be exploited to accommodate a broader range of chemotypes that retain reasonable antischistosomal efficacy *in vitro* and potency at this target. This feature could represent an opportunity to design new PZQ analogues with improved DMPK (drug metabolism and pharmacokinetics) parameters matching the physicochemical properties associated with known antischistosomal compounds.^48^

Even though mesenteric vein concentration is the main driver of PZQ efficacy,^49^ higher exposure (i.e., AUC or *C*_max_), inferior plasma protein binding, lower PgP efflux, or reduced phase I metabolization may be some of the PK outputs that would likely improve upon PZQ analogues’ efficacy *in vivo* (especially against juveniles). As reported with PZQ metabolite, 4OH-PZQ, less potent but higher exposure metabolites may still contribute to the clinical efficacy of the drug.^50^ These “new” metabolites may prove to be more efficacious than 4OH-PZQ in killing schistosomes of all lifecycle stages.

## Conclusions

This study investigated the schistosomicidal potential of a novel series of 1*H*-1,2,3-triazole derivatives of PZQ and their synthetic intermediates. In total, six out of 11 PZQ analogues demonstrated activity against schistosomula during the initial screening: three triazoles (**5b**, **5c**, **5e**) and three synthetic intermediates (**2**, **3**, **4**). Among these, compounds **3**, **4**, and **5e** were considered the most promising, as they also exhibited a significant effect against juvenile and adult worms while maintaining good selective indices over the parasites compared to the HepG2 human cell line. TRPM channel activation studies revealed that compounds **2**, **3**, **4**, and **5e** are able to modulate *Sm*.TRPM_PZQ_, sharing a similar activation profile to PZQ. Similar to PZQ, they did not activate *Fh*.TRPM_PZQ_ or *Sm*.TRPM_MCLZ_ at the tested concentrations. These compounds, along with **5d**—a PZQ analogue with no detectable activity on schistosomula, adult worms, or *Sm*.TRPM_PZQ_—were also able to activate *Eg*.TRPM_PZQ_. Altogether, these findings indicate that compounds **3**, **4**, and **5e** can be used as molecular scaffolds for the rational design of novel PZQ analogues with schistosomicidal activity. The latter can be achieved by exploring the newly revealed plasticity of the *Sm*.TRPM_PZQ_ through Y1517 side chain rotation to accommodate modifications on position 9 of the pyrazinoisoquinoline nucleus of PZQ. Finally, future studies will focus on studying the pharmacokinetics and pharmacodynamic (PK/PD) profiles of these compounds *in vivo* to further explore their potential as PZQ surrogates.

## Materials and Methods

### Compound Synthesis

Reagents and methods are described in detail in Supporting Information.

### Biology

#### Parasites

Experiments involving schistosomula were performed at Aberystwyth University. For *S. mansoni* (Naval Medical Research Institute; NMRI strain) lifecycle maintenance, six- to twenty-six-week-old female TO (HsdOLa:TO-Tuck Ordinary; Envigo, UK) mice (6–12 animals/month) were percutaneously exposed by immersion of their tails in water containing 180 cercariae for 45 min. At 47 days post infection, animals were administered an intraperitoneal injection of a lethal dose of sodium pentobarbital containing 100 U/mL heparin, and parasites were collected by reverse perfusion of the hepatic-portal venous system. Following perfusion, eggs were recovered from infected livers, miracidia hatched, and used to infect susceptible *Biomphalaria glabrata* snails (NMRI albino and pigmented outbred strains).^51^ All procedures performed on mice (project licenses 40/3700 and P3B8C46FD) adhered to the United Kingdom Home Office Animals (Scientific Procedures) Act of 1986 as well as the European Union Animals Directive 2010/63/EU and were approved by Aberystwyth University’s (AU) Animal Welfare and Ethical Review Bodies (AWERB).

For the collection of schistosomula, *B. glabrata* snails infected with *S. mansoni* were shed for 2 h under light at 26 °C. Cercariae were collected, mechanically transformed into schistosomula as previously described.^52^

For the production of juvenile and adult schistosomes, Swiss Webster mice (∼60 days old) were inoculated subcutaneously with 400 (adults) or 1000 (juveniles) *S. mansoni* cercariae (BH strain), provided by the Laboratory of Malacology (IOC/Fiocruz). After 21 (juveniles) or 42 (adults) days of infection, the animals were euthanized, and the parasites were collected through perfusion of the mesenteric and portal-hepatic veins.^53–55^ Adult worms were then transferred to a Petri dish containing DMEM medium (Vitrocell) supplemented with 10% fetal bovine serum and kept overnight in an incubator (37 °C and 5% CO_2_). Juvenile worms were sedimented successively following washing with serum-free DMEM (microscopy analysis) or M199 medium (supplemented with l-glutamine, sodium bicarbonate, 2% calf serum, penicillin-streptomycin 100 IU/mL penicillin −0.10 mg/mL streptomycin solution, and no phenol red), for XTT assay. All procedures with animals were conducted in accordance with current national legislation and under authorization from the Ethics Committee on the Use of Animals (CEUA/IOC/FIOCRUZ, Brazil; License number L-012/2023).

#### Schistosomula Assay

The effect of PZQ analogues on the motility and morphological features of schistosomula after 72 h of incubation was evaluated using a high-content screening assay (“Roboworm”), as previously described.^42,43^ PZQ derivatives were tested at a final concentration of 50 μM (in 0.625% DMSO). PZQ and Auranofin (AUR) were included as positive controls at a final concentration of 10 μM (in 0.625% v/v DMSO). DMSO (0.625%, v/v) was included as a negative control. Z′ values for the assay were calculated to be 0.542 for phenotype and 0.361 for motility.

#### XTT Juvenile Viability Assay

To determine the viability of juvenile worms after exposure to the PZQ analogues, a method based on the metabolic reduction of the soluble tetrazolium salt XTT (2,3-Bis­(2-Methoxy-4-Nitro-5-Sulfophenyl)-2H-Tetrazolium-5-Carboxanilide) was adapted from Aguiar et al, 2017.^56^ More details are provided in the Supporting Information.

#### Microscopy Analysis of Juvenile Morphology and Motility

The effect of PZQ analogues on parasites’ morphology and motility was assessed visually using microscopy images as described in the Supporting Information.

#### Adult Worm Ex Vivo High-Content Assay

Adult schistosomes’ motility was determined using a high-content assay previously developed by our group,^44,54^ with images analyzed in CellProfiler software.^57^ More details are provided in the Supporting Information.

#### Cytotoxicity Assay

The *in vitro* cytotoxic activity of the PZQ analogues was evaluated in hepatic HepG2 (BCRJ 0103) and kidney WSS-1 (ATCC CRL-2029) epithelial human cell lines by using a fluorometric viability assay based on the metabolic reduction of resazurin into resorufin. More details are provided in the Supporting Information.

#### Ca^2+^ Reporter Assay

The Ca^2+^ reporter assay was performed using a Fluorescence Imaging Plate Reader (FLIPR) system, as described in the Supporting Information.

#### Molecular Modeling

The ligand–protein complex models were constructed based on the methodology previously described for the R-PZQ *Sm.*TRPM_PZQ_ complex.^26^ Despite experimental results were generated with the racemic mixtures of all PZQ derivatives, only the R-enantiomers of all compounds were modeled considering this is the known eutomer for PZQ binding at *Sm.*TRPM_PZQ_.^26^ Ligands were manually edited using the Maestro software suite,^58^ and residues within a 10 Å radius of each ligand were optimized in UCSF Chimera^59^ using the AMBER force field with Gasteiger partial charges. Energy minimization was performed using 5000 steps of steepest descent, followed by 500 steps of conjugate gradient. Cavity surface was calculated using Chimerax.^60^ All molecular graphics and visualizations were generated with UCSF Chimera.

## Supplementary Material



## Notes

Experimental Section detailing compound synthesis along with analytical data and NMR spectra (Figures S1–S26), XTT juvenile viability assay, microscopy analysis of juvenile morphology and motility, adult worm ex vivo high-content assay, cytotoxicity assay, and Ca^2+^ reporter assay. Supporting (Figures S27–S40) and (Tables S1–S4) (PDF)

## Abbreviations

DALYs: disability-adjusted life years
DMEM: Dulbecco’s modified eagle medium
FBS: fetal bovine serum
HBSS: Hank’s balanced salt solution
HEPES: 4-(2-hydroxyethyl)-1-piperazineethanesulfonic acid
INLA: integrated nested laplace approximation
MCLZ: meclonazepam
MDA: mass drug administration
MHR: TRPM homology region (MHR)
NUDT9H: nudix-type hydrolase domain 9 homologue
PZQ: Praziquantel
SI: selectivity index
TRP: transient receptor potential
TRPM: transient receptor potential channels of the melastatin subfamily
TRPM_MCLZ_: transient receptor potential channel of the melastatin subfamily activated by meclonazepam
TRPM_PZQ_: transient receptor potential channel of the melastatin subfamily activated by praziquantel
VSLD: voltage-sensor-like domain (VSLD)
WHO: world health organization
XTT: 2,3-Bis­(2-Methoxy-4-Nitro-5-Sulfophenyl)-2*H*-Tetrazolium-5-Carboxanilide
