# Supplementary material for: Novel 1H‑1,2,3-Triazole Derivatives of Praziquantel with TRPMPZQ Modulatory Activity and Antiparasitic Effects on Larvae, Juvenile, and Adult Worms of Schistosoma mansoni
Source: ACS Infect Dis. 2025 Dec 10;12(4):1301–12. doi: 10.1021/acsinfecdis.5c00914 (PMC12718081; doi:10.1021/acsinfecdis.5c00914)
Supplement: Supplementary file 1 [file id5c00914_si_001.pdf]

Supplementary material

Novel 1H-1,2,3-triazole derivatives of Praziquantel with $TRPM_{PZQ}$ modulatory activity and antiparasitic effects on larvae, juvenile and adult worms of *Schistosoma mansoni*

Floriano Paes Silva Junior,[1,+,*] Rafael Ferreira Dantas,[1,+] Sang-Kyu Park,[2] Helen Whiteland,[3] Camilla Thomaz da Silva Oliveira,[4] Joao M Rezende-Neto,[1] Jordano Ferreira Reis,[1] Josephine Forde-Thomas,[3] Luciano Pinho Gomes,[1] Walter C.G. Valente,[1] Giuliana Viegas Schirato,[1] Frederico Ricardo de Castro Noronha Jr,[4] Karl F. Hoffmann,[3] Jonathan S. Marchant,[2] Sabrina Baptista Ferreira,[4,*]

(1) Laboratório de Bioquímica Experimental e Computacional de Fármacos, Instituto Oswaldo Cruz, Fiocruz, Brazil; (2) Department of Cell Biology, Neurobiology & Anatomy, Medical College of Wisconsin, 8701 Watertown Plank Road, Milwaukee, WI 53226, USA; (3) Department of Life Sciences, Aberystwyth University, Edward Llwyd Building, Penglais, Aberystwyth,UK; (4) Laboratório de Síntese Orgânica e Prospecção Biológica, Universidade Federal do Rio de Janeiro, Instituto de Química, Departamento de Química Orgânica, 21941-590, Rio de Janeiro, Brazil.

KEYWORDS: schistosomiasis drug discovery, *Schistosoma mansoni*, praziquantel analogues, triazoles, phenotypic screening, TRPM modulators.

[+] These authors contributed equally to this work.

*Corresponding authors: Chemistry: sabrinab@iq.ufrj.br; Biology: floriano@ioc.fiocruz.br

Chemistry

Compound synthesis

General reagents. Reagents were used as purchased without further purification. Reagents were purchased from Aldrich or LabSynth. Column chromatography was performed with silica gel 60 (Merck 70-230 mesh). Analytical thin-layer chromatography was performed with silica gel plates (Merck, TLC silica gel 60 F254), and the plots were visualized under UV light or developed by immersion in an ethanolic solution of vanillin or ninhydrin, followed by heating. Solvent removal was conducted under reduced pressure using an IKA rotary evaporator with a thermostatic bath and controlled temperature, followed by drying under high vacuum to eliminate residual solvents. Yields refer to chromatographically and spectroscopically homogeneous materials. Melting points were obtained on a Fisatom digital melting point apparatus, model 430 D. Infrared spectra data were recorded from KBr pellets on a Perkin-Elmer model 1420 FT-IR Spectrophotometer, calibrated relative to the 1,601.8 cm^{-1} absorbance of polystyrene. NMR spectra were acquired using Bruker instruments operating at 7.05 Tesla (300 MHz for ^1H and 75 MHz for ^{13}C) and 11.74 Tesla (500 MHz for ^1H and 125 MHz for ^{13}C). Spectral widths were set at 10.0 ppm for ^1H and 240 ppm for ^{13}C, with a relaxation delay of 2 seconds. Samples (20–25 mg) were dissolved in 0.6 mL of $CDCl_3$ containing 0.05% v/v TMS as internal reference (0.00 ppm). Parameters for 2D NMR experiments followed those described in the literature. Additional equipment employed a Parr hydrogenation apparatus operating under a hydrogen atmosphere at 35 PSI. Elemental analysis was used to ascertained purity ≥95% for all compounds for which biological data were determined.

(2-(Cyclohexanecarbonyl)-10-nitro-2,3,6,7-tetrahydro-1H-pyrazino[2,1-a]isoquinolin-4(11bH)-one) (**2**). In a 500 mL round-bottom flask, praziquantel 1 (3.00 g; 9.60 mmol) and concentrated sulfuric acid (5.14 mL; 96 mmol) were added. The flask was then placed in an ice bath (−5 °C) under magnetic stirring, and concentrated nitric acid (2.12 mL; 48 mmol) was slowly added dropwise. After complete addition, the reaction mixture was maintained under the same conditions for 4 hours. Reaction progress was monitored by thin-layer chromatography (TLC). Upon completion, while still in the ice bath, approximately 150 mL of distilled water was added, and the pH was adjusted to 8 by the addition of sodium bicarbonate. Subsequently, 150 mL of ethyl acetate was added, and the mixture was transferred to a separatory funnel. The aqueous phase was washed twice with 100 mL of ethyl acetate, and the combined organic layers were dried over anhydrous sodium sulfate (Na_2SO_4), filtered, and the solvent was removed using a rotary evaporator. The resulting product was subjected to vacuum drying to remove residual solvents. Since TLC analysis indicated a mixture of substances, purification was carried out by flash column chromatography on silica gel (40–63 µm), using a gradient elution starting with ethyl acetate:petroleum ether (3:1, v/v) and ending with pure ethyl acetate. The isolated fraction was concentrated under reduced pressure and subjected to vacuum drying to eliminate residual solvents, yielding a yellowish solid in 88% yield. TLC Rf: 0.69 (eluent: ethyl acetate/petroleum ether, 3:1).

9-Amino-2-(ciclohexanecarbonyl)-2,3,6,7-tetraydro-1H-pyrazino[2,1-a]isoquinolin-4(11bH)-one (**3**). In a hydrogenation reactor, compound 9-NO_2-PZQ 2 (0.90 g; 2.56 mmol) and palladium on

activated carbon (10%) (0.11 g; 2.56 mmol) were added. Subsequently, 10 mL of ethyl acetate was introduced, and the reaction mixture was stirred under hydrogen gas at a pressure of 35 PSI for approximately 3 hours. The reaction progress was monitored by ^{1}H NMR spectroscopy, which confirmed complete conversion to the desired product. Upon completion, the reaction mixture was filtered three times through filter paper. The solvent was removed under reduced pressure using a rotary evaporator, followed by drying under high vacuum to eliminate residual solvents, affording a brown solid in 80% yield. TLC Rf: 0.78 (eluent: ethyl acetate/petroleum ether, 3:1).

9-Azido-2-(ciclohaxenocarbonyl)-2,3,6,7-tetraydro-1H-pyrazino[2,1-a]isoquinolin-4(11bH)-one (**4**). In a 50 mL flask, compound 3 (0.200 g; 0.61 mmol) was added and maintained in an ice bath with stirring. Subsequently, 5 mL of a 6 M HCl solution was slowly added. After 15 minutes, a dropwise addition of a 0.6 M sodium nitrite solution was performed. After 45 minutes, a cooled 0.8 M sodium azide solution was added slowly. The reaction conditions were maintained for an additional hour. Finally, 15 mL of water was added, and the mixture was extracted three times with 50 mL portions of ethyl acetate in a separatory funnel. The organic phase was washed twice with saturated sodium bicarbonate solution, dried over Na_2SO_4, filtered, and the solvent was removed under rotary evaporation. The residual solvent was then eliminated under vacuum. The resulting product was a light brown oil with an 87% yield. Rf: 0.58 (Eluent: Ethyl acetate / Petroleum ether - 3:1).

2-(ciclohexanecarbonyl)-9-(4-fenil-1H-1,2,3-tryazol-1-yl)-1,2,3,6,7,11b-hexahydro-4H-pyrazino[2,1-a]isoquinolina-4-one (**5a**). In a G30 vial (microwave-compatible), compound 4 (0.059 g; 0.17 mmol) and phenylacetylene (0.04 mL; 0.34 mmol) were dissolved in 5 mL of tert-butanol. Next, $CuSO_4 \cdot 5H_2O$ (0.084 g; 0.34 mmol) and sodium ascorbate (0.067 g; 0.34 mmol) were added. Then, rapidly, 5 mL of water and a magnetic stir bar were added. The vial was sealed and subjected to microwave irradiation for 5 minutes under conditions of 50 °C, 0 bar pressure, and 600 rpm stirring. The consumption of starting material was monitored by TLC, and it was necessary to repeat the microwave irradiation at least two more times, totaling 15 minutes of reaction time. After the reaction, the mixture was poured into a flask, to which 15 mL of water was added, and tert-butanol was removed by rotary evaporation. The resulting solution was extracted three times with 50 mL portions of ethyl acetate in a separatory funnel. The organic phases were combined, dried over Na_2SO_4, filtered, and the solvent was removed under rotary evaporation. The residual solvent was then eliminated under vacuum, yielding a pale yellow solid with a 98% yield. Rf: 0.38 (Eluent: Ethyl acetate / Hexane – 4:1).

9-(4-(ciclohex-1-en-1-yl)-1H-1,2,3-tryazol-1-il)-2-(ciclohexenocarbonyl)-1,2,3,6,7,11b-hexahydro-4H-pyrazino[2,1-a]isoquinolina-4-ona (**5b**). In a G30 vial, compound 4 (0.059 g; 0.19 mmol) and 1-ethynylcyclohexene (0.05 mL; 0.38 mmol) were dissolved in 5 mL of tert-butanol. Subsequently, $CuSO_4 \cdot 5H_2O$ (0.097 g; 0.38 mmol) and sodium ascorbate (0.077 g; 0.38 mmol) were added. Then, 5 mL of water and a magnetic stirring bar were quickly added. The vial was sealed and subjected to microwave irradiation for 5 minutes under the following conditions: 50°C/0 bar/600 rpm. The consumption of the starting material was monitored by TLC, and it was

necessary to repeat the microwave conditions at least two more times, totaling 15 minutes of reaction. After completion, the reaction mixture was transferred to a round-bottom flask, 15 mL of water was added, and the tert-butanol was removed under reduced pressure using a rotary evaporator. The aqueous phase was then extracted three times with 50 mL portions of ethyl acetate using a separatory funnel. The combined organic layers were dried over Na_2SO_4, filtered, and the solvent was removed under reduced pressure. Residual solvents were eliminated under high vacuum. TLC appeared to indicate complete conversion, but post-extraction, a mixture of compounds was observed, which was separated by preparative TLC. Finally, a pale yellow solid was obtained in 95% yield. Rf: 0.31 (eluent: ethyl acetate/hexane – 4:1).

2-(ciclohexanecarbonyl)-9-(4-(fenoxymetil)-1H-1,2,3-tryazol-1-yl)-1,2,3,6,7,11b-hexahydro-4H-pyrazino[2,1-a]isoquinolina-4-one (**5c**). In a G30 vial, compound 4 (0.155 g; 0.44 mmol) and phenylpropargyl ether (0.116 g; 0.88 mmol) were dissolved in 5 mL of tert-butanol. Then, $CuSO_4·5H_2O$ (0.220 g; 0.88 mmol) and sodium ascorbate (0.174 g; 0.88 mmol) were added. Subsequently, 5 mL of H_2O was added, and the vial was placed in a microwave reactor for 5 minutes under the following conditions: 50 °C / 0 bar / 600 rpm. The consumption of the starting material was monitored by TLC, and it was necessary to repeat the microwave conditions at least two more times, totaling 15 minutes of reaction.

After completion of the reaction, 15 mL of water was added to the mixture, and the tert-butanol was removed using a rotary evaporator. The reaction mixture was extracted three times with 50 mL portions of ethyl acetate in a separatory funnel. The organic phase was dried over Na_2SO_4, filtered, and the solvent was removed under reduced pressure using a rotary evaporator. Any remaining solvent residues were removed under high vacuum. Finally, a yellowish oil was obtained in over 99% yield. Rf: 0.38 (eluent: ethyl acetate/hexane – 4:1).

2-(ciclohexanecarbonyl)-9-(4-(1-hydroxyciclohexyl)-1H-1,2,3-tryazol-1-yl)-1,2,3,6,7,11b-hexahydro-4H-pyrazino[2,1-a]isoquinolina-4-one (**5d**). In a G30 vial, compound 4 (0.177 g; 0.50 mmol) and 1-ethynyl-1-cyclohexanol (0.124 g; 1.00 mmol) were dissolved in 8 mL of tert-butanol. Subsequently, $CuSO_4·5H_2O$ (0.249 g; 1.00 mmol) and sodium ascorbate (0.198 g; 1.00 mmol) were added. Then, an additional 8 mL of tert-butanol, 8 mL of water, and a magnetic stir bar were added. The vial was sealed and subjected to microwave irradiation for 5 minutes under the following conditions: 50 °C / 0 bar / 600 rpm. Consumption of the starting material was monitored by TLC, and it was necessary to repeat the microwave conditions once more, totaling 10 minutes of reaction.

After completion of the reaction, 15 mL of water was added to the mixture and the tert-butanol was removed using a rotary evaporator. The reaction mixture was extracted three times with 50 mL portions of ethyl acetate using a separatory funnel. The combined organic phases were dried over Na_2SO_4, filtered, and the solvent was removed under reduced pressure using a rotary evaporator. Final solvent traces were removed under high vacuum. TLC indicated complete conversion to the desired product. However, following extraction, the presence of another substance—possibly a degradation product—was observed. This impurity was removed by

washing with hexane:ethyl acetate (10:1). Finally, a yellow solid was obtained in 88% yield. Rf: 0.20 (eluent: ethyl acetate/hexane – 4:1).

2-(ciclohexanecarbonyl)-9-(4-propil-1H-1,2,3-tryazol-1-yl)-1,2,3,6,7,11b-hexahydro-4H-pyrazino[2,1-a]isoquinolina-4-one (**5e**). In a 50 mL round-bottom flask, compound 4 (0.159 g; 0.45 mmol) and 1-pentyne (0.061 g; 0.90 mmol) were dissolved in 5 mL of dry tert-butanol. Subsequently, $CuSO_4 \cdot 5H_2O$ (0.224 g; 0.90 mmol) and sodium ascorbate (0.178 g; 0.90 mmol) were added. Finally, 5 mL of water was added, and the reaction mixture was subjected to microwave irradiation for 5 minutes under the following conditions: 50 °C / 0 bar / 600 rpm. The consumption of starting material was monitored by TLC, and it was necessary to repeat the microwave conditions at least twice, resulting in a total reaction time of 15 minutes. Due to the high volatility of 1-pentyne, two additional 0.1 mL portions were added during the reaction. Upon completion, 15 mL of water was added, and tert-butanol was removed using a rotary evaporator. The mixture was extracted three times with 50 mL portions of ethyl acetate in a separatory funnel. The combined organic layers were dried over Na_2SO_4, filtered, and the solvent was evaporated under reduced pressure using a rotary evaporator. Residual solvent was removed under high vacuum. A dark yellow solid was obtained in >99% yield. Rf: 0.346 (eluent: ethyl acetate/hexane – 4:1).

2-(ciclohexanecarbonyl)-10-(4-(((3aS,5aR,8aR,8bS)-2,2,7,7-tetrametyltetrahydro-3aH-bis([1,3]dioxolo)[4,5-b:4',5'-d]pyran-3a-yl)metyl)-1H-1,2,3-tryazol-1-yl)-1,2,3,6,7,11b-hexahydro-4H-pyrazino[2,1-a]isoquinolina-4-one (**5f**). In a G30 vial, compound 4 (0.063 g; 0.19 mmol) and protected fructose-alkyne derivative (0.106 g; 0.36 mmol) were added. Subsequently, $CuSO_4 \cdot 5H_2O$ (0.088 g; 0.36 mmol) and sodium ascorbate (0.070 g; 0.36 mmol) were added. Then, 5.5 mL of tert-butanol, 5.5 mL of water, and a magnetic stir bar were added. The vial was sealed and subjected to microwave irradiation for 5 minutes under the conditions of 50 °C / 0 bar / 600 rpm. The consumption of the starting material was monitored by TLC, and it was necessary to repeat the microwave irradiation conditions once more, resulting in a total reaction time of 10 minutes. TLC indicated complete conversion to the desired product. After completion of the reaction, 15 mL of water was added and tert-butanol was removed using a rotary evaporator. The reaction mixture was extracted three times with 50 mL of ethyl acetate using a separatory funnel. The organic layers were combined, dried over Na_2SO_4, filtered, and the solvent was removed under reduced pressure using a rotary evaporator. Residual solvent traces were eliminated under high vacuum. A pale-yellow solid was obtained in 92% yield. Rf: 0.23 (eluent: ethyl acetate/hexane – 4:1).

2-(ciclohexanecarbonyl)-9-(4-(hydroxymetyl)-1H-1,2,3-tryazol-1-yl)-1,2,3,6,7,11b-hexahydro-4H-pyrazino[2,1-a]isoquinolina-4-one (**5g**). In a G30 vial, compound 4 (0.056 g; 0.16 mmol) and propargyl alcohol (0.04 mL; 0.32 mmol) were added. Subsequently, $CuSO_4 \cdot 5H_2O$ (0.080 g; 0.32 mmol) and sodium ascorbate (0.063 g; 0.32 mmol) were introduced. Then, 5 mL of tert-butanol, 5 mL of water, and a magnetic stir bar were added. The vial was sealed and subjected to microwave irradiation for 5 minutes under the conditions of 50 °C / 0 bar / 600 rpm.

Consumption of the starting material was monitored by TLC, and it was necessary to repeat the microwave irradiation conditions two additional times. During this process, an additional amount of propargyl alcohol (0.036 g; 0.32 mmol) was added, bringing the total reaction time to 15 minutes. Final TLC analysis showed complete conversion to the desired product. After completion of the reaction, 15 mL of water was added, and tert-butanol was removed using a rotary evaporator. The reaction mixture was extracted three times with 50 mL of ethyl acetate using a separatory funnel. The organic layers were combined, dried over Na_2SO_4, filtered, and the solvent was removed under reduced pressure using a rotary evaporator. Residual traces of solvent were eliminated under high vacuum. A pale yellow, highly hygroscopic solid was obtained in 47% yield. Rf: 0.04 (eluent: ethyl acetate/petroleum ether – 3:1).

(1-(2-(ciclohexanecarbonyl)-4-oxy-1,3,4,6,7,11b-hexahydro-2H-pyrazino[2,1-a]isoquinolina-9-yl)-1H-1,2,3-tryazol-4-yl)metyl acetato (**5h**). In a G30 vial, compound 4 (0.056 g; 0.16 mmol) and the corresponding alkyne (0.04 mL; 0.40 mmol) were added. Subsequently, $CuSO_4 \cdot 5H_2O$ (0.080 g; 0.32 mmol) and sodium ascorbate (0.063 g; 0.32 mmol) were added. Then, 5 mL of tert-butanol, 5 mL of water, and a magnetic stir bar were introduced. The vial was sealed and subjected to microwave irradiation for 5 minutes under the following conditions: 50 °C / 0 bar / 600 rpm. Consumption of the starting material was monitored by TLC, and it was necessary to repeat the microwave conditions two more times. During this process, an additional amount of the alkyne (0.032 mL; 0.32 mmol) was added, resulting in a total reaction time of 15 minutes. Final TLC analysis showed complete conversion to the desired product. After completion of the reaction, 15 mL of water was added to the mixture, and tert-butanol was removed using a rotary evaporator. The reaction mixture was extracted three times with 50 mL of ethyl acetate using a separatory funnel. The organic layers were combined, dried over Na_2SO_4, filtered, and the solvent was removed under reduced pressure in a rotary evaporator. Residual solvent traces were removed under high vacuum. A pale yellow, highly hygroscopic solid was obtained in 42% yield. Rf: 0.14 (eluent: ethyl acetate/petroleum ether – 3:1).

Analytical data

*(2-(Cyclohexanecarbonyl)-10-nitro-2,3,6,7-tetrahydro-1H-pyrazino[2,1-a]isoquinolin-4(11bH)-one) (**2**)*
IR (KBr, ν, cm^{-1}): 3482.4; 3294.3; 3062.3; 2929.8; 2854.1; 2661.4; 1733.6; 1653.7; 1588.8; 1523.7; 1419.4; 1346.7; 1283.2; 1245.1; 1213.4; 1172.9; 1101.4; 999.4; 894.9; 820.3; 734.9; 645.2; 442.2.^{1}H NMR (300 MHz, $CDCl_3$, δ ppm): 8.21 (s, 1H); 8.11 (d, J = 8.2 Hz, 1H); 7.39 (d, J = 8.4 Hz, 1H); 5.27 (d, J = 13.3 Hz, 1H); 4.93–4.85 (m, 2H); 4.51 (d, J = 17.5 Hz, 1H); 4.12 (d, J = 7.1 Hz, 1H); 4.10 (dd, J = 13.0, 5.9 Hz, 1H); 3.10–2.87 (m, 4H); 2.48 (t, J = 11.2 Hz, 1H); 1.77–1.24 (m, 10H).^{13}C NMR (75 MHz, $CDCl_3$, δ ppm): 174.9; 164.5; 147.1; 142.5; 134.6; 130.6; 122.6; 121.0; 60.5; 54.8; 49.1; 44.8; 40.8; 38.6; 29.3; 29.1; 25.8; 21.1; 14.3. HRMS (ESI) m/z: [M+Na]+ calcd for $C_{19}H_{23}N_3O_4$ 380,1689 found 380.1731

9-Amino-2-(ciclohexanecarbonyl)-2,3,6,7-tetraydro-1H-pyrazino[2,1-a]isoquinolin-4(11bH)-one (**3**)
IR (KBr, ν, cm^{-1}): 3440.5; 3349.7; 3234.5; 3009.1; 2853.5; 2664.4; 1731.5; 1640.6; 1511.4; 1422.2; 1359.8; 1327.7; 1280.4; 1215.1; 1125.9; 1082.0; 999.1; 821.5; 648.1; 603.3; 488.5.^{1}H NMR (300

MHz, $CDCl_3$, δ ppm): 6.95 (d, J = 8.7 Hz, 1H); 6.58 (d, J = 5.4 Hz, 2H); 5.14–5.01 (m, 1H); 4.81–4.65 (m, 2H); 4.45 (d, J = 17.4 Hz, 1H); 4.15–4.03 (m, 1H); 3.95–3.48 (m, 2H); 2.86–2.62 (m, 4H); 2.46 (t, J = 11.3 Hz, 1H); 1.81–1.26 (m, 10H).^{13}C NMR (75 MHz, $CDCl_3$, δ ppm): 174.9; 164.5; 145.4; 133.6; 130.2; 124.4; 114.8; 111.6; 60.4; 55.1; 49.1; 45.4; 40.9; 39.6; 29.2; 27.9; 25.8; 21.1; 14.3.; HRMS (ESI) m/z: [M+Na]+ calcd for $C_{19}H_{25}N_3O_2$ 350.1947 found 350.1932

9-Azido-2-(ciclohaxenocarbonyl)-2,3,6,7-tetraydro-1H-pyrazino[2,1-a]isoquinolin-4(11bH)-one (**4**)
IR (KBr, cm^{-1}): 3483.4; 3290.9; 3228.6; 3050.1; 2930.6; 2853.8; 2661.7; 2407.8; 2228.4; 2111.9; 1735.3; 1645.6; 1501.0; 1418.5; 1359.3; 1280.8; 1215.7; 1137.6; 1082.4; 998.9; 893.6; 818.5; 729.9; 646.9; 594.2; 532.0; 459.2; ^{1}H NMR (300 MHz, $CDCl_3$): δ 7.18 (d, J = 8.7 Hz, 1H); 6.92 (s, 2H); 5.13 (d, J = 13.5 Hz, 1H); 4.80 (dd, J = 15.6, 10.3 Hz, 2H); 4.48 (d, J = 17.5 Hz, 1H); 4.09 (dd, J = 15.7, 8.5 Hz, 1H); 2.95–2.74 (m, 4H); 2.47 (t, J = 11.3 Hz, 1H); 1.77–1.23 (m, 10H).^{13}C NMR (75 MHz, $CDCl_3$): δ 174.9; 164.5; 139.1; 134.6; 131.5; 131.2; 118.3; 116.1; 60.5; 55.0; 49.1; 45.1; 40.9; 39.2; 29.3; 29.1; 28.3; 25.8; 21.1; 14.3; HRMS (ESI) m/z: [M+Na]+ calcd for $C_{19}H_{23}N_5O_2$ 376.1852 found 376.1760

2-(ciclohexanecarbonyl)-9-(4-fenil-1*H*-1,2,3-tryazol-1-yl)-1,2,3,6,7,11b-hexahydro-4*H*-pyrazino[2,1-a]isoquinolin-4-one (**5a**)
IR (KBr, cm^{-1}): 3449.2; 3147.6; 3061.7; 3031.4; 3004.5; 2926.4; 2854.4; 2662.2; 1733.6; 1636.7; 1515.0; 1448.0; 1359.7; 1259.6; 1210.0; 1127.8; 1042.1; 892.8; 828.6; 761.9; 693.3; 456.0; ^{1}H NMR (300 MHz, $CDCl_3$): δ 8.27 (s, 1H); 7.92 (d, J = 7.2 Hz, 2H); 7.86–7.66 (m, 2H); 7.47 (t, J = 7.4 Hz, 2H); 7.38 (t, J = 7.6 Hz, 2H); 5.15 (d, J = 13.2 Hz, 1H); 4.95–4.81 (m, 2H); 4.49 (d, J = 17.6 Hz, 1H); 4.17–4.08 (m, 1H); 2.97 (dt, J = 30.7, 13.5 Hz, 4H); 2.47 (t, J = 11.1 Hz, 1H); 1.77–1.23 (m, 10H).^{13}C NMR (75 MHz, $CDCl_3$): δ 175.0; 164.5; 148.7; 136.0; 135.6; 134.7; 131.0; 130.2; 129.3; 128.6; 126.0; 119.9; 117.7; 117.2; 55.0; 49.2; 44.9; 40.8; 39.2; 29.3; 29.1; 28.5; 21.2; 14.3; HRMS (ESI) m/z: [M+Na]+ calcd for $C_{27}H_{29}N_5O_2$ 478.2321 found 478.2202

9-(4-(ciclohex-1-en-1-yl)-1*H*-1,2,3-tryazol-1-il)-2-(ciclohexenocarbonyl)-1,2,3,6,7,11b-hexahydro-4*H*-pyrazino[2,1-a]isoquinolin-4-one (**5b**)
IR (KBr, cm^{-1}): 3422.2; 3134.7; 2930.7; 2856.3; 2666.7; 1640.6; 1509.7; 1448.5; 1422.2; 1325.0; 1260.2; 1220.3; 1042.4; 893.3; 824.1; 648.2.^{1}H NMR (300.00 MHz, $CDCl_3$) δ: 7.84 (s, 1H); 7.72 (d, J = 8.5 Hz, 1H); 7.61 (s, 1H); 7.34 (d, J = 8.3 Hz, 1H); 6.66 (s, 1H); 5.14 (d, J = 13.6 Hz, 1H); 4.86 (d, J = 8.5 Hz, 2H); 4.49 (d, J = 17.3 Hz, 1H); 4.12 (dd, J = 12.5, 5.3 Hz, 1H); 2.96 (dt, J = 45.7, 14.9 Hz, 5H); 2.45 (d, J = 1.9 Hz, 2H); 2.24 (s, 2H); 1.76–1.25 (m, 14H).^{13}C NMR (75.00 MHz, $CDCl_3$) δ: 175.0; 164.5; 136.2; 135.3; 134.6; 130.9; 127.0; 126.2; 119.8; 117.0; 116.3; 55.1; 49.2; 45.0; 40.9; 39.2; 29.4; 28.5; 26.6; 25.8; 25.5; 22.6; 22.3; 21.1; 14.3. HRMS (ESI) m/z: [M+Na]+ calcd for $C_{27}H_{33}N_5O_2$ 482.2630 found 482.2620

2-(ciclohexanecarbonyl)-9-(4-(phenoxymethyl)-1H-1,2,3-tryazol-1-yl)-1,2,3,6,7,11b-hexahydro-4H-pyrazino[2,1-a]isoquinolin-4-one (**5c**)
IR (KBr, cm^{-1}): 3465.0; 3283.4; 3138.4; 3065.1; 3039.3; 2929.8; 2854.2; 2663.1; 2449.8; 1733.1; 1649.2; 1598.4; 1494.4; 1421.4; 1295.4; 1239.4; 1174.9; 1080.1; 1033.9; 887.6; 824.0; 754.8; 691.7; 509.8.^{1}H NMR (300.00 MHz, $CDCl_3$) δ: 8.11 (s, 1H); 7.67 (s, 1H); 7.35–7.29 (m, 3H); 7.05–6.96 (m, 4H); 5.29 (s, 2H); 5.13 (d, J = 9.6 Hz, 1H); 4.85 (d, J = 8.1 Hz, 1H); 4.69 (d, J = 2.4 Hz, 1H); 4.48 (d, J = 17.4 Hz, 1H); 4.11 (dd, J = 12.6, 5.4 Hz, 1H); 3.06–2.80 (m, 4H); 2.51 (dd, J = 14.3, 7.0 Hz, 1H); 1.80–1.23 (m, 10H).^{13}C NMR (75.00 MHz, $CDCl_3$) δ: 174.9; 164.5; 158.2; 157.6; 135.9; 134.7; 130.9; 129.7; 121.6; 121.4; 120.9; 119.8; 117.5; 114.8; 78.7; 75.6; 62.0; 55.8; 55.0; 49.1; 44.8; 40.8; 39.1; 29.3; 28.4; 25.7; 21.1; 14.3. HRMS (ESI) m/z: [M+Na]+ calcd for $C_{28}H_{31}N_5O_3$ 508.2427 found 508.2020

*2-(ciclohexanecarbonyl)-9-(4-(1-hydroxyciclohexyl)-1H-1,2,3-tryazol-1-yl)-1,2,3,6,7,11b-hexahydro-4H-pyrazino[2,1-a]isoquinolin-4-one (**5d**)*

IR (KBr, cm^{-1}): 3405.3; 3307.6; 3138.4; 2931.8; 2854.6; 2663.0; 1733.0; 1639.4; 1511.1; 1447.2; 1324.9; 1258.7; 1178.1; 1040.9; 966.9; 903.9; 824.3; 646.5. ^1H NMR (300.00 MHz, $CDCl_3$) δ: 7.99 (s, 1H); 7.70 (d, J = 8.3 Hz, 1H); 7.65 (s, 1H); 7.34 (d, J = 8.2 Hz, 1H); 5.13 (d, J = 12.6 Hz, 1H); 4.89–4.84 (m, 2H); 4.49 (d, J = 17.3 Hz, 1H); 4.17–4.08 (m, 2H); 3.01–2.87 (m, 5H); 2.56 (s, 1H); 2.49 (d, J = 11.4 Hz, 1H); 1.92 (d, J = 3.8 Hz, 2H); 1.79 (s, 2H); 1.73–1.69 (m, 4H); 1.58 (d, J = 10.6 Hz, 6H); 1.27 (d, J = 7.1 Hz, 4H). ^{13}C NMR (75.00 MHz, $CDCl_3$) δ: 175.0; 164.5; 135.5; 134.6; 130.9; 119.9; 117.4; 88.4; 72.2; 68.6; 60.5; 55.0; 49.2; 44.9; 40.8; 39.9; 39.1; 38.2; 29.3; 28.5; 25.7; 23.2; 22.1; 21.1; 14.3. HRMS (ESI) m/z: [M+Na]+ calcd for $C_{27}H_{35}N_5O_3$ 500.2740 found 500.2138

*2-(ciclohexanecarbonyl)-9-(4-propil-1H-1,2,3-tryazol-1-yl)-1,2,3,6,7,11b-hexahydro-4H-pyrazino[2,1-a]isoquinolin-4-one (**5e**)*

IR (KBr, cm^{-1}): 3596.6; 3472.5; 3341.3; 3137.8; 3087.3; 3007.6; 2927.7; 2855.9; 2667.8; 1735.7; 1646.4; 1517.7; 1418.7; 1322.5; 1219.0; 1085.2; 1056.9; 1001.1; 896.0; 820.5; 727.4; 644.0; 580.9; 535.4; 460.1.^1H NMR (300.00 MHz, $CDCl_3$) δ: 7.81 (s, 1H); 7.70 (d, J = 7.6 Hz, 1H); 7.64 (s, 1H); 7.34 (d, J = 7.8 Hz, 1H); 5.12 (d, J = 12.7 Hz, 1H); 4.86 (d, J = 7.7 Hz, 3H); 4.48 (d, J = 17.1 Hz, 1H); 4.12 (d, J = 17.6 Hz, 1H); 2.99–2.79 (m, 6H); 2.47 (s, 1H); 1.76 (d, J = 7.0 Hz, 3H); 1.55 (d, J = 10.9 Hz, 2H); 1.27 (d, J = 6.9 Hz, 4H); 1.03 (t, J = 7.1 Hz, 5H).^{13}C NMR (75.00 MHz, $CDCl_3$) δ: 206.9; 175.0; 164.5; 136.3; 135.3; 134.6; 130.8; 119.8; 117.2; 55.0; 49.2; 44.9; 40.8; 39.1; 30.9; 29.3; 28.4; 27.8; 25.75–25.7; 22.6; 13.8. HRMS (ESI) m/z: [M+Na]+ calcd for $C_{24}H_{31}N_5O_2$ 444.2478 found 444.2470

*2-(ciclohexanecarbonyl)-10-(4-(((3aS,5aR,8aR,8bS)-2,2,7,7-tetramethyltetrahydro-3aH-bis([1,3]dioxolo)[4,5-b:4',5'-d]pyran-3a-yl)methyl)-1H-1,2,3-tryazol-1-yl)-1,2,3,6,7,11b-hexahydro-4H-pyrazino[2,1-a]isoquinolin-4-one (**5f**)*

IR (KBr, cm^{-1}): 3387.4; 3128.7; 3085.1; 3018.7; 2929.2; 2853.9; 2664.8; 2243.8; 1631.3; 1513.2; 1422.6; 1361.4; 1328.0; 1246.9; 1127.2; 1040.5; 979.2; 918.8; 891.1; 871.0; 822.2; 795.4; 694.7; 535.1; 494.8; 410.6. ^1H NMR (300.00 MHz, $CDCl_3$) δ: 8.07 (s, 1H); 7.72 (s, 1H); 7.64 (s, 1H); 7.35 (d, J = 7.7 Hz, 1H); 5.16 (d, J = 12.3 Hz, 1H); 4.89 (s, 2H); 4.61 (d, J = 6.6 Hz, 1H); 4.41 (s, 1H); 4.24 (d, J = 7.4 Hz, 2H); 4.13 (s, 1H); 3.90 (s, 1H); 3.72 (t, J = 14.0 Hz, 5H); 3.02–2.87 (m, 4H); 2.04 (s, 1H); 1.73 (s, 2H); 1.42 (d, J = 6.1 Hz, 10H); 1.29 (d, J = 17.9 Hz, 12H).^{13}C NMR (75.00 MHz, $CDCl_3$) δ: 175.0; 164.5; 136.1; 135.6; 134.8; 130.9; 119.7; 117.6; 109.0; 108.7; 102.7; 77.6; 77.2; 76.7; 72.2; 71.1; 70.3; 65.6; 61.2; 55.0; 49.2; 44.9; 40.8; 39.1; 34.7; 31.7; 29.3; 29.1; 28.5; 26.6; 25.9; 25.7; 25.5; 25.4; 24.1; 22.7; 14.2. HRMS (ESI) m/z: [M+Na]+ calcd for $C_{33}H_{43}N_5O_7$ 644.3162 found 644.3159

*2-(ciclohexanecarbonyl)-9-(4-(hydroxymethyl)-1H-1,2,3-tryazol-1-yl)-1,2,3,6,7,11b-hexahydro-4H-pyrazino[2,1-a]isoquinolin-4-one (**5g**)*

IR (KBr, cm^{-1}): 3387.4; 3128.7; 3085.1; 3018.7; 2929.2; 2853.9; 2664.8; 2243.8; 1631.3; 1513.2; 1422.6; 1361.4; 1328.0; 1246.9; 1127.2; 1040.5; 979.2; 918.9; 891.1; 871.0; 822.2; 795.4; 728.5; 592.3; 494.8.^1H NMR (300.00 MHz, $CDCl_3$) δ: 8.12 (s, 1H); 7.75–7.50 (m, 2H); 7.40–7.27 (m, 1H); 5.10 (d, J = 13.5 Hz, 1H); 4.85 (d, J = 12.9 Hz, 4H); 4.49 (d, J = 17.4 Hz, 1H); 4.13 (d, J = 17.5 Hz, 1H); 3.09–2.80 (m, 4H); 2.51 (dd, J = 34.7, 23.6 Hz, 2H); 1.80–1.21 (m, 10H).^{13}C NMR (75.00 MHz, $CDCl_3$) δ: 175.1; 164.5; 148.9; 135.9; 135.6; 134.6; 130.8; 120.3; 119.7; 117.5; 56.4; 55.0; 49.2; 45.0; 40.8; 39.1; 29.3; 29.1; 28.4; 25.7; 25.6. HRMS (ESI) m/z: [M+Na]+ calcd for $C_{23}H_{29}N_5O_3$ 446.2270 found 446.2271

*(1-(2-(ciclohexanecarbonyl)-4-oxy-1,3,4,6,7,11b-hexahydro-2H-pyrazino[2,1-a]isoquinolin-9-yl)-1H-1,2,3-tryazol-4-yl)methyl acetate (**5h**)*

IR (KBr, cm^{-1}): 3408.8; 3138.2; 3094.2; 2930.1; 2854.6; 2663.7; 1740.3; 1640.7; 1512.5; 1421.6; 1362.4; 1326.0; 1295.6; 1241.5; 1127.1; 1041.1; 893.3; 826.3; 729.8; 645.3; 594.6; 535.9; 492.0.

^{1}H NMR (300.00 MHz, $CDCl_3$) δ: 8.16–8.11 (m, 1H); 7.76–7.63 (m, 2H); 7.34 (t, J = 8.3 Hz, 1H); 5.29 (s, 1H); 5.12 (t, J = 9.5 Hz, 1H); 4.90–4.82 (m, 3H); 4.49 (d, J = 17.5 Hz, 1H); 4.12 (d, J = 17.6 Hz, 1H); 3.06–2.84 (m, 5H); 2.47 (t, J = 10.9 Hz, 1H); 2.11 (s, 2H); 1.44 (dd, J = 103.5, 36.0 Hz, 10H).^{13}C NMR (75.00 MHz, $CDCl_3$) δ: 175.1; 171.0; 164.5; 148.9; 143.8; 136.5; 135.9; 130.8; 122.1; 120.3; 119.7; 117.6; 57.6; 56.4; 55.0; 49.2; 44.9; 40.8; 39.1; 29.3; 28.5; 25.7; 20.9. HRMS (ESI) m/z: [M+Na]+ calcd for $C_{24}H_{29}N_5O_4$ 474.2220 found 474.2218

NMR spectra

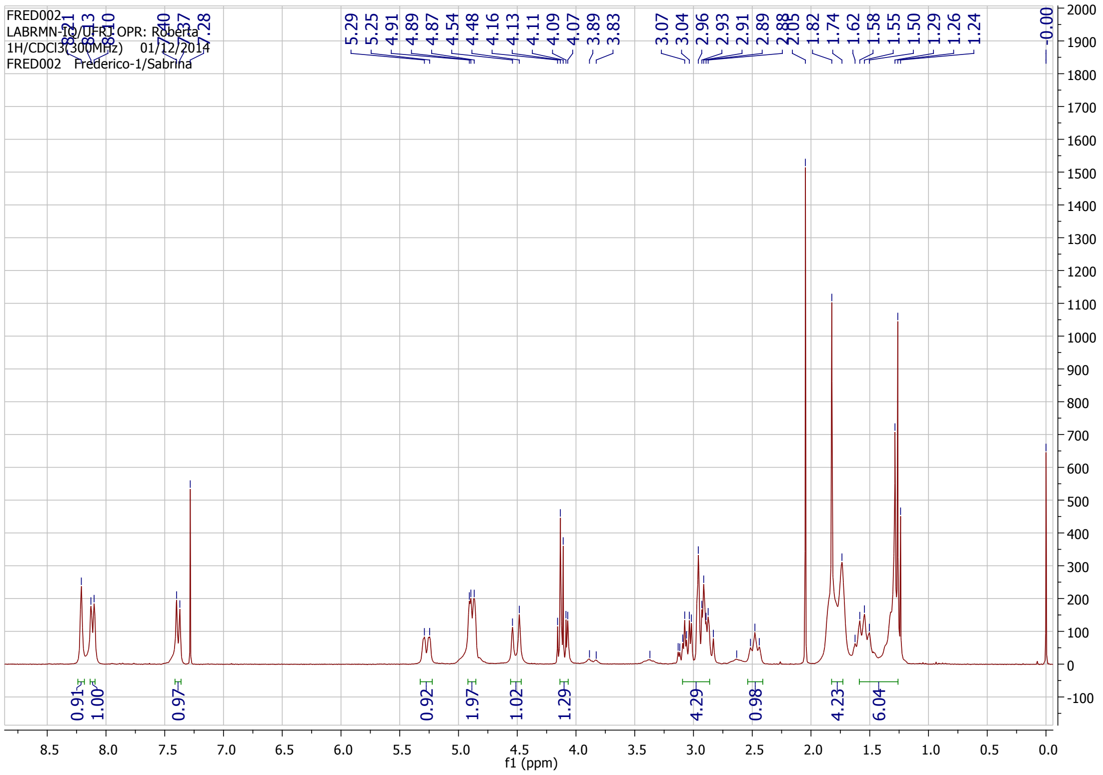

Figure S1 - ^{1}H NMR (300 MHz, $CDCl_3$) of **2**.

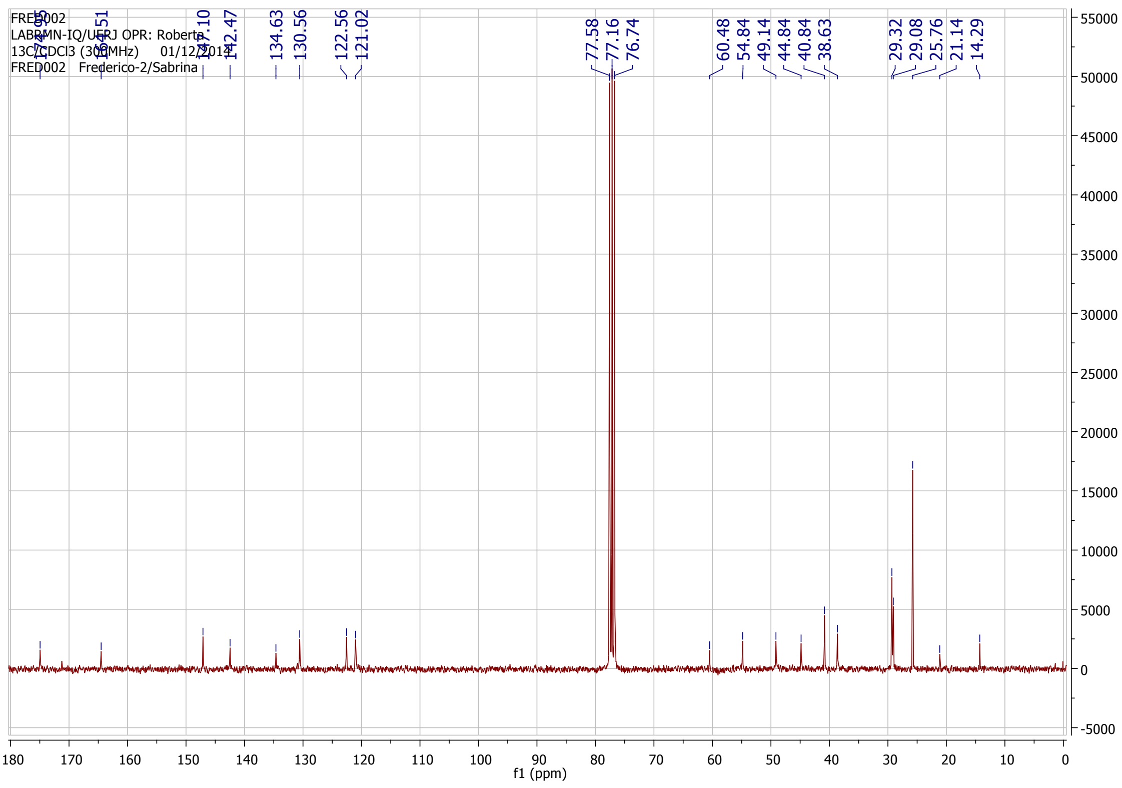

Figure S2 - ^{13}C NMR (75 MHz, $CDCl_3$) of **2**.

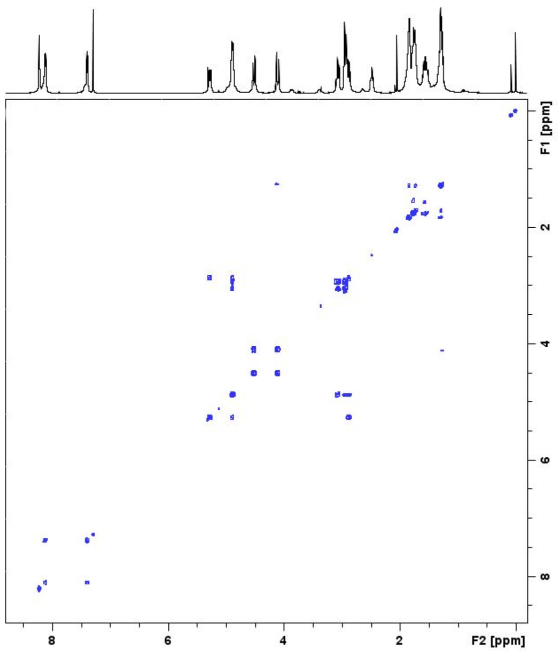

Figure S3 - COSY (500 MHz, $CDCl_3$) of 2.

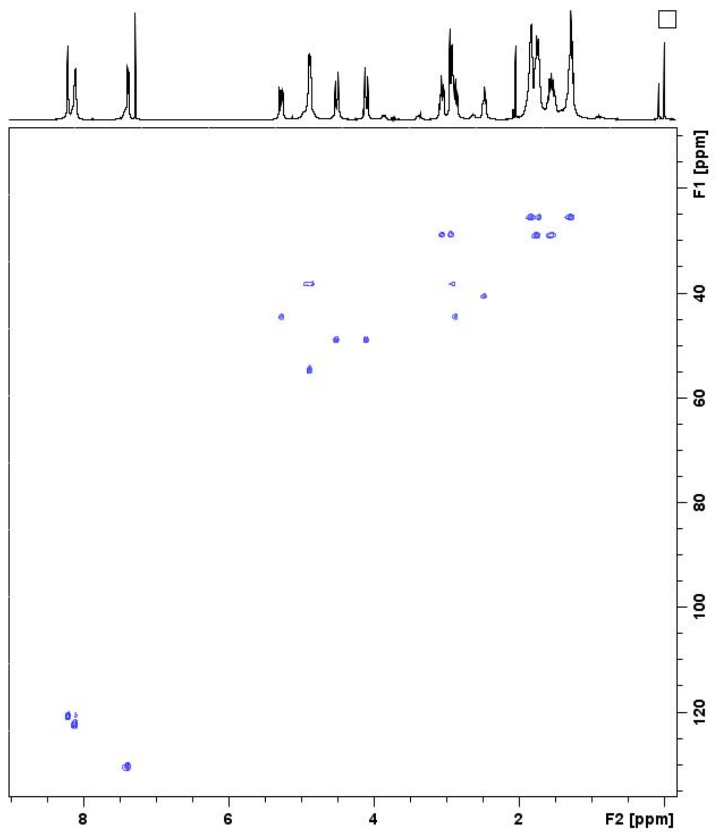

Figure S4 - HSQC (500 MHz, $CDCl_3$) of **2**.

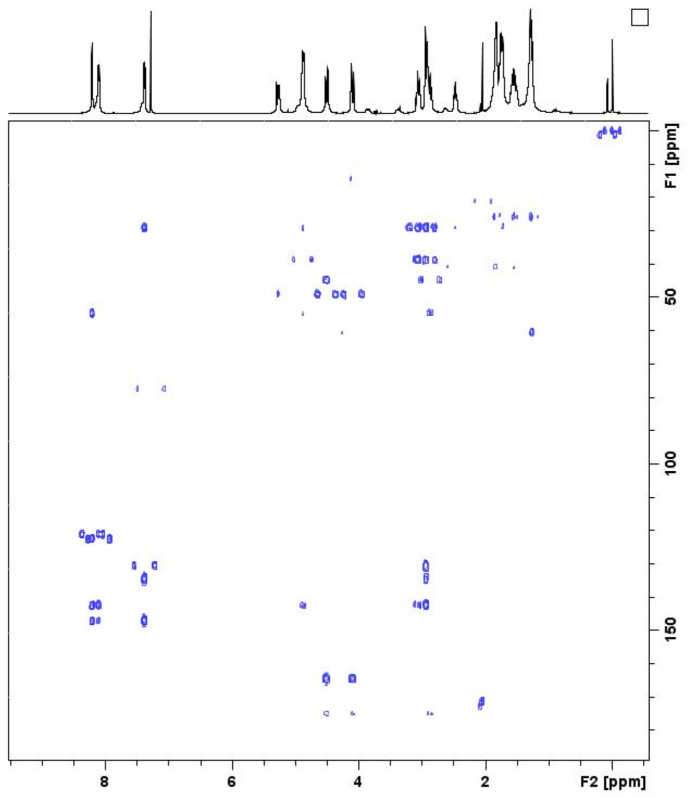

Figure S5 - HMBC (500 MHz, $CDCl_3$) of **2**.

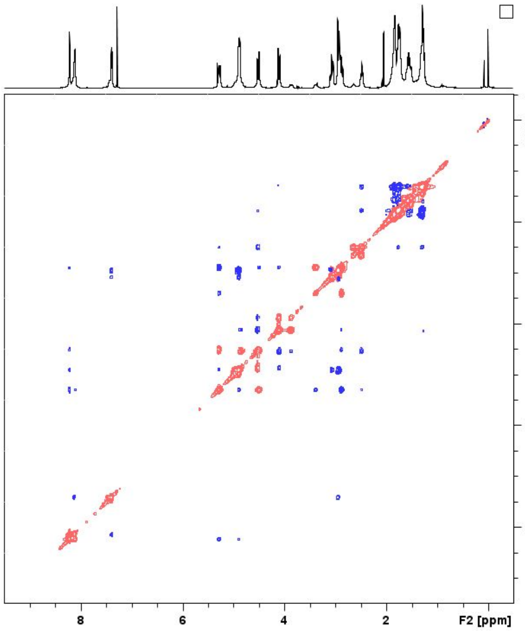

Figure S6 - NOESY (500 MHz, $CDCl_3$) of **2**.

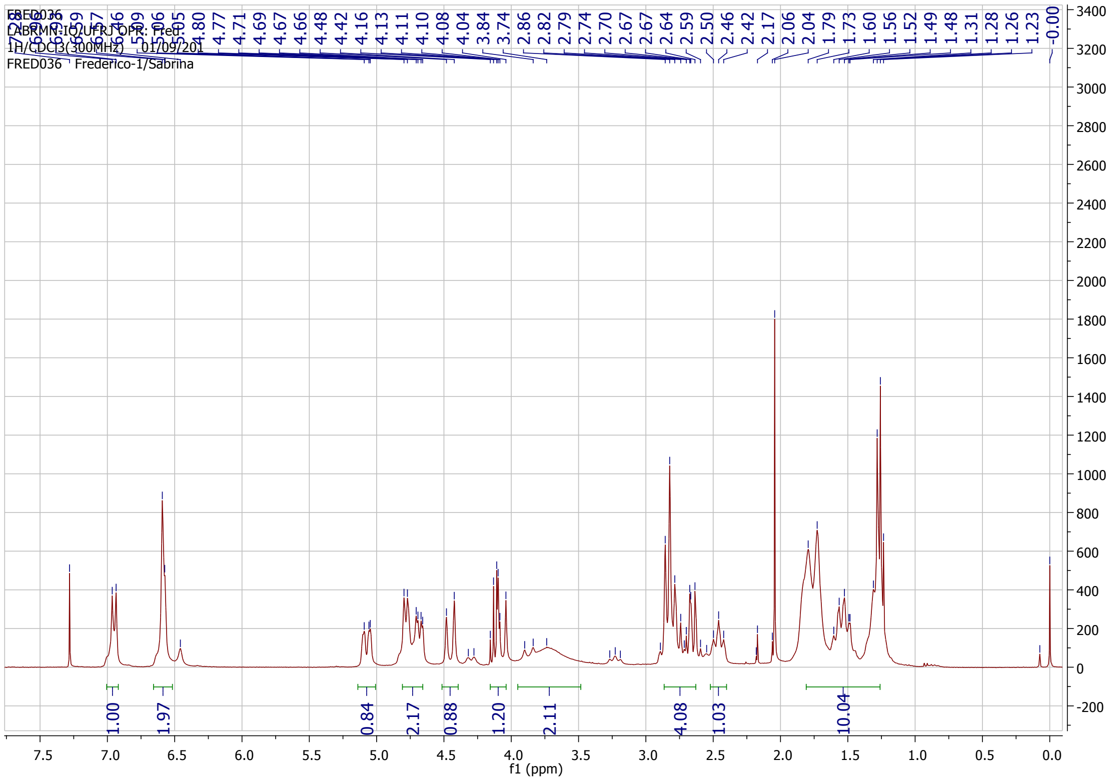

Figure S7 - ^{1}H NMR (300 MHz, $CDCl_3$) of **3**.

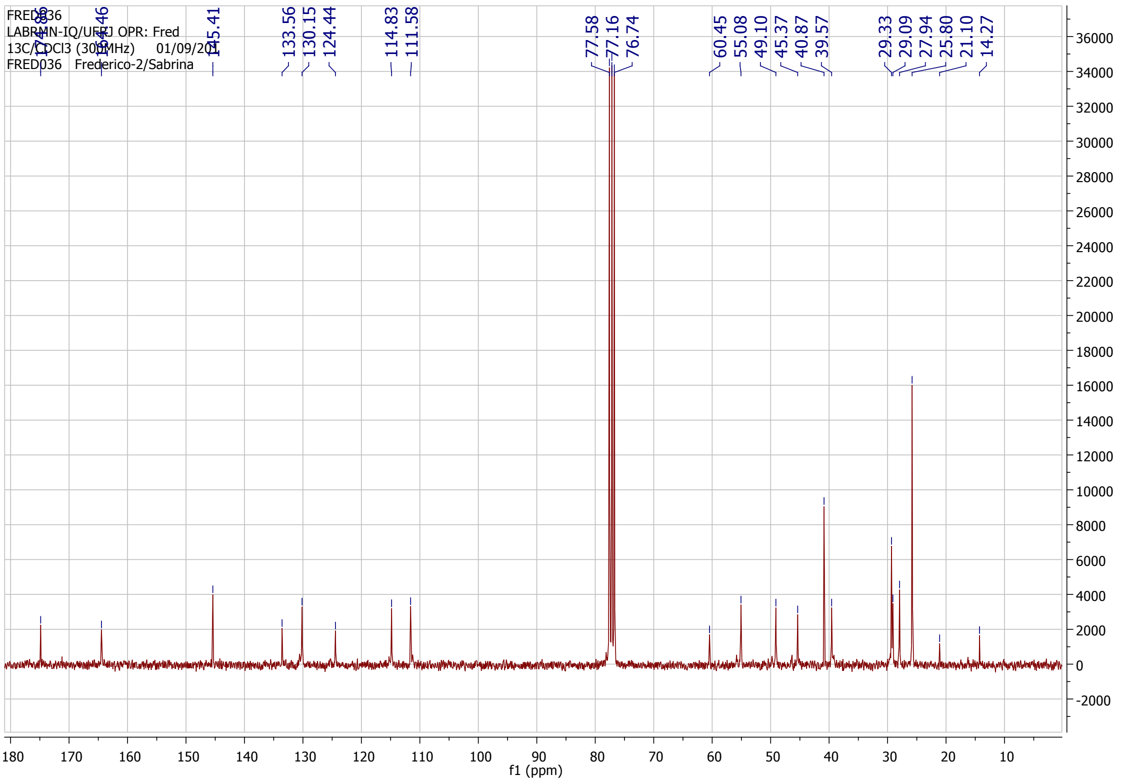

Figure S8 - ^{13}C NMR (75 MHz, $CDCl_3$) of **3**.

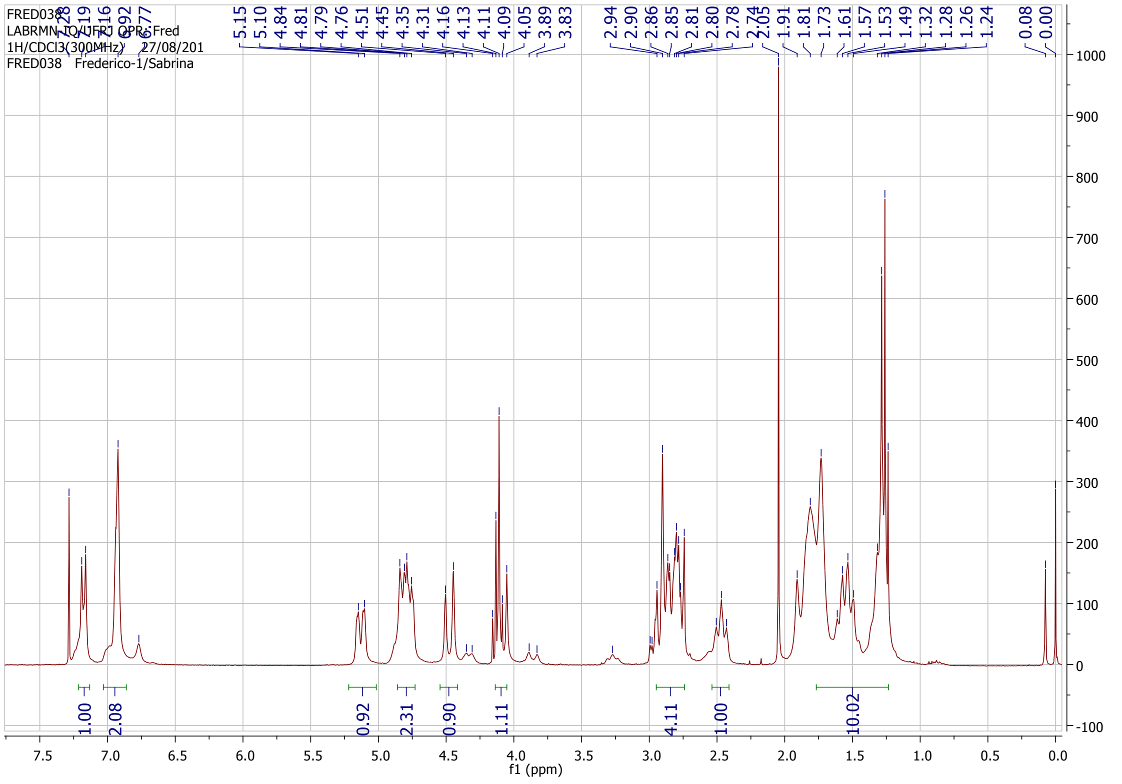

Figure S9 - ^{1}H NMR (300 MHz, $CDCl_3$) of **4**.

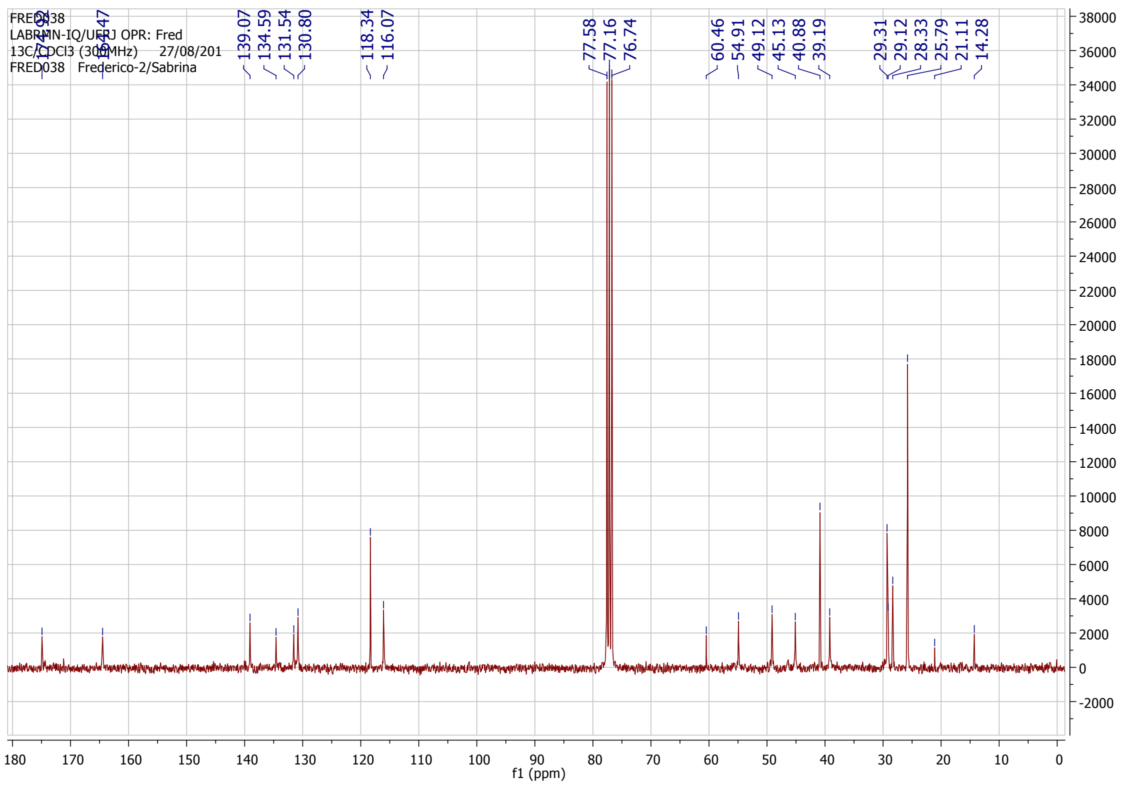

Figure S10 - ^{13}C NMR (75 MHz, $CDCl_3$) of **4**.

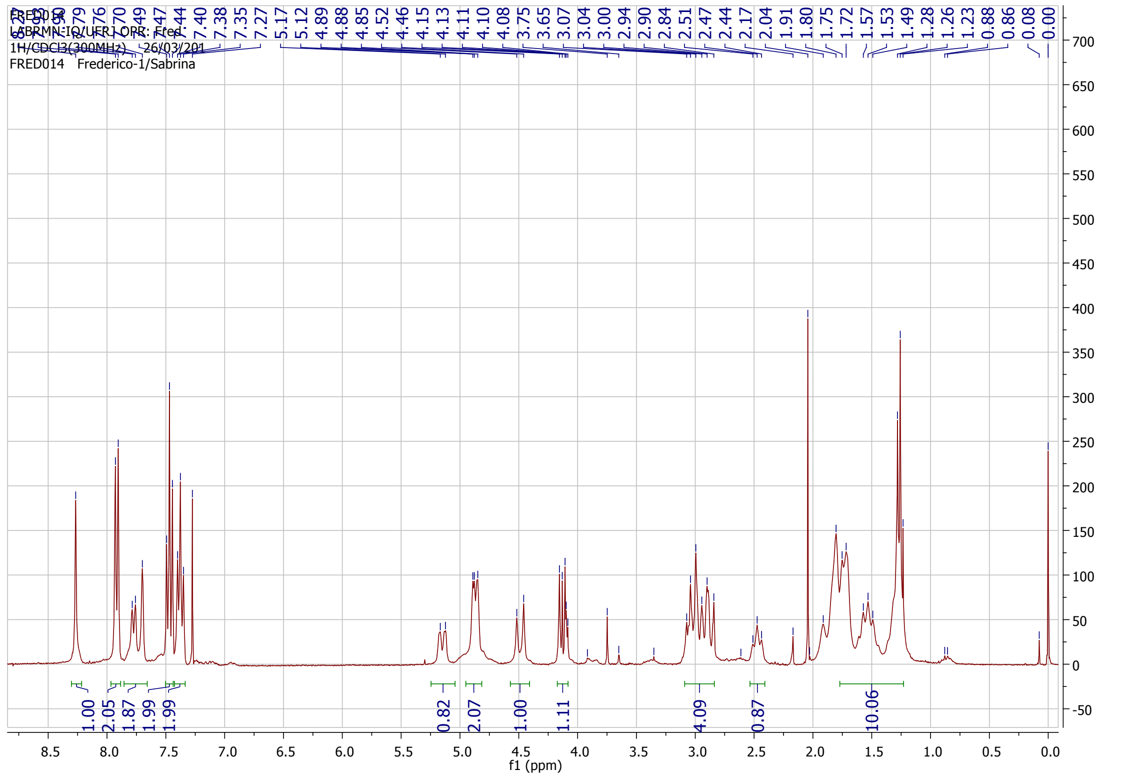

Figure S11 - ^{1}H NMR (300 MHz, $CDCl_3$) of **5a**.

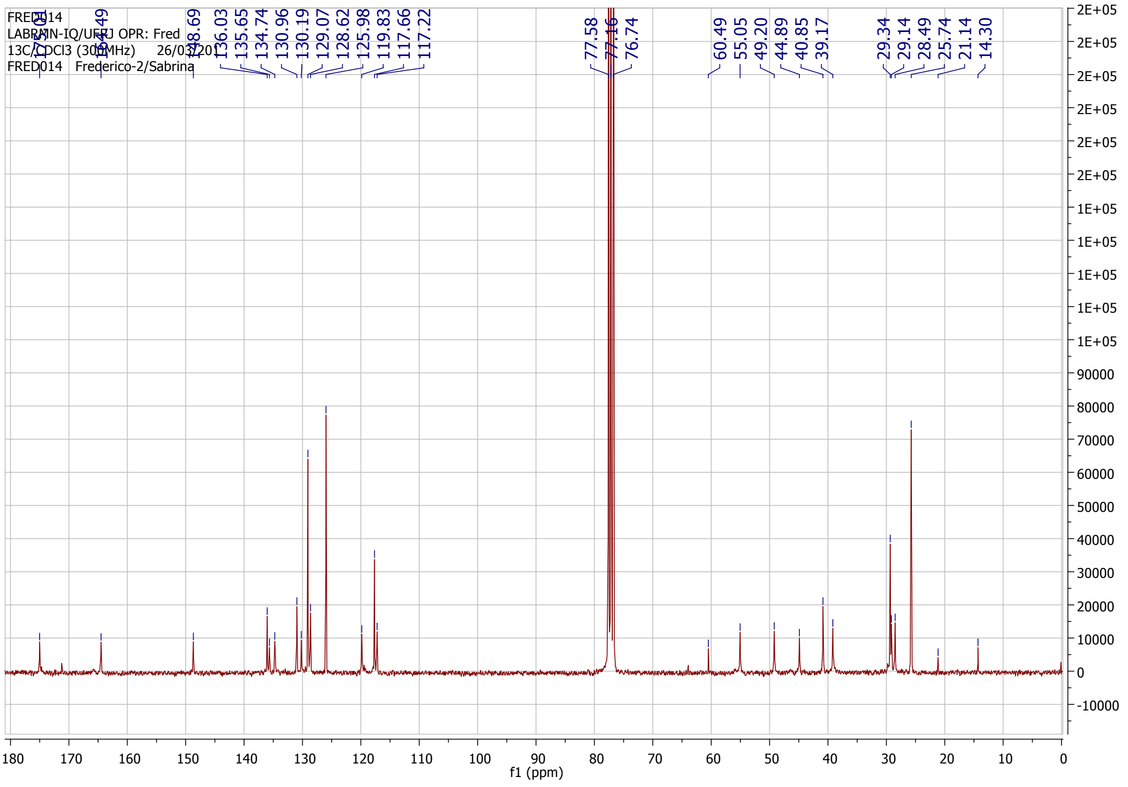

Figure S12 - ^{13}C NMR (75 MHz, $CDCl_3$) of **5a**.

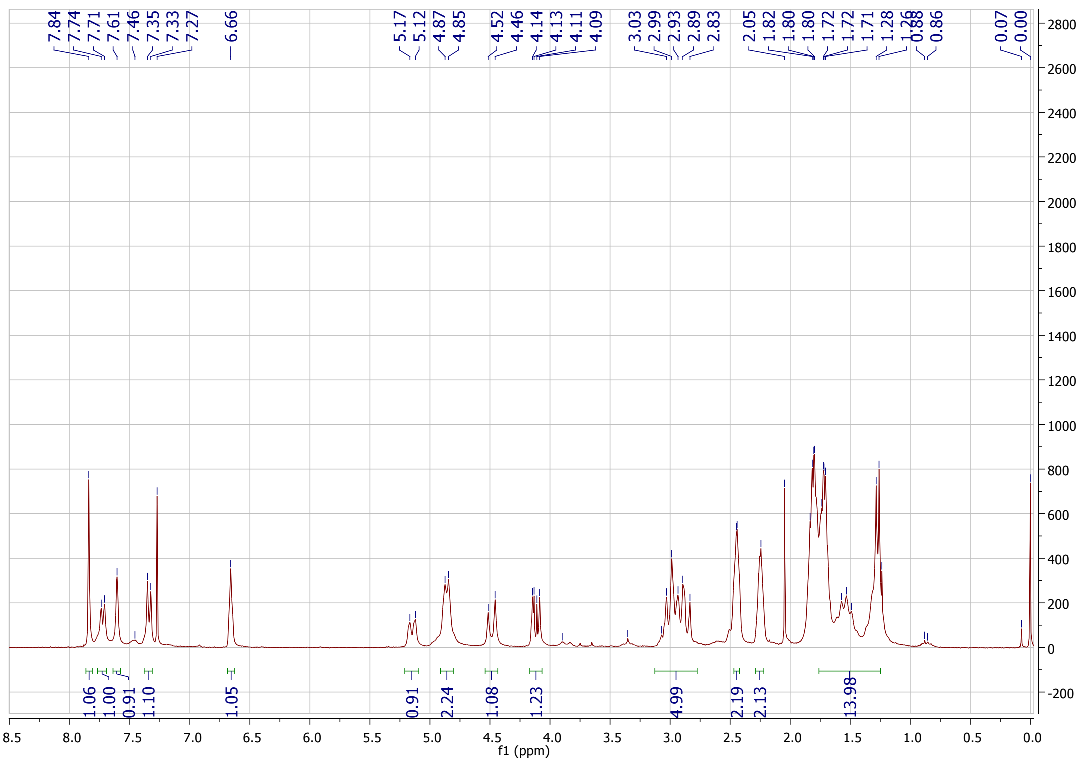

Figure S13 - ^{1}H NMR (300 MHz, $CDCl_3$) of **5b**.

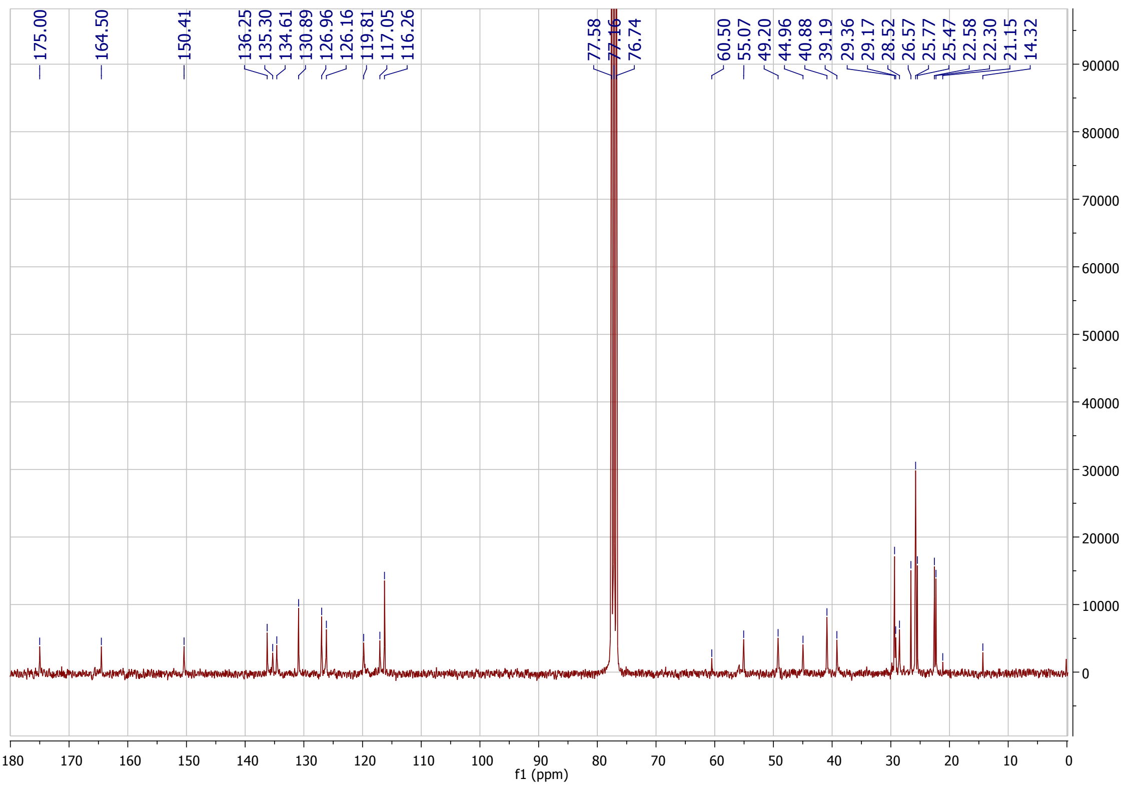

Figure S14 - ^{13}C NMR (75 MHz, $CDCl_3$) of **5b**.

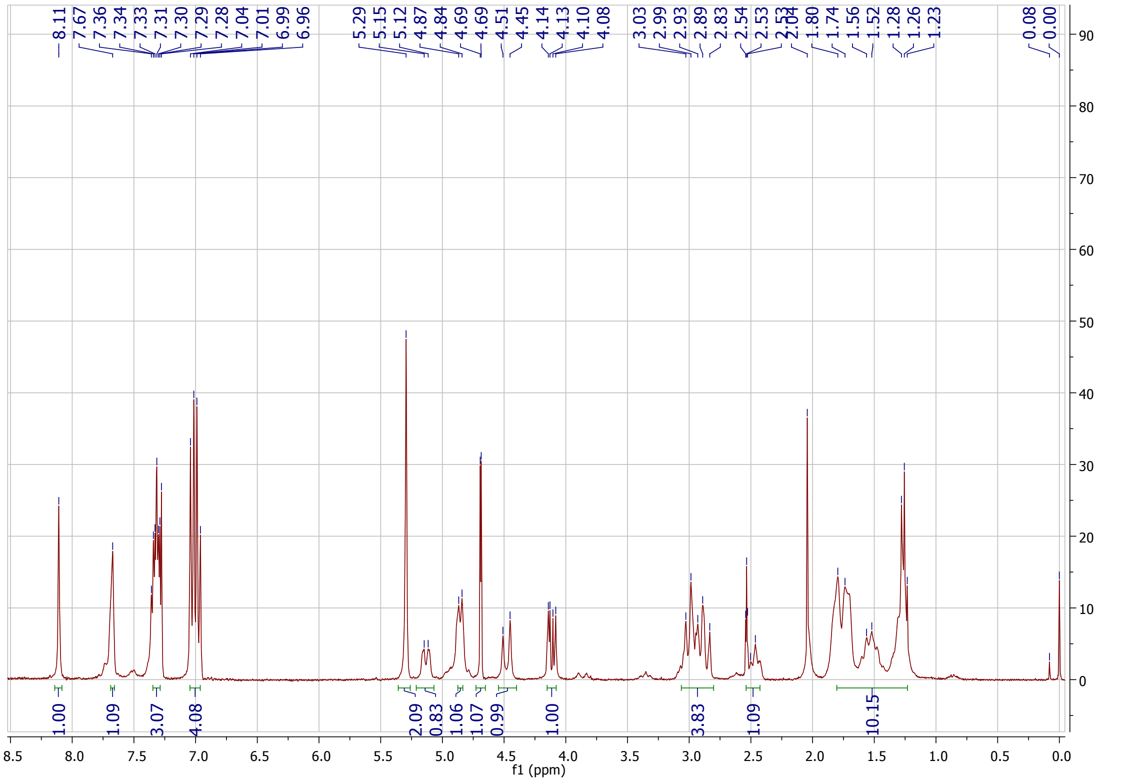

Figure S15 - ^{1}H NMR (300 MHz, $CDCl_3$) of **5c**.

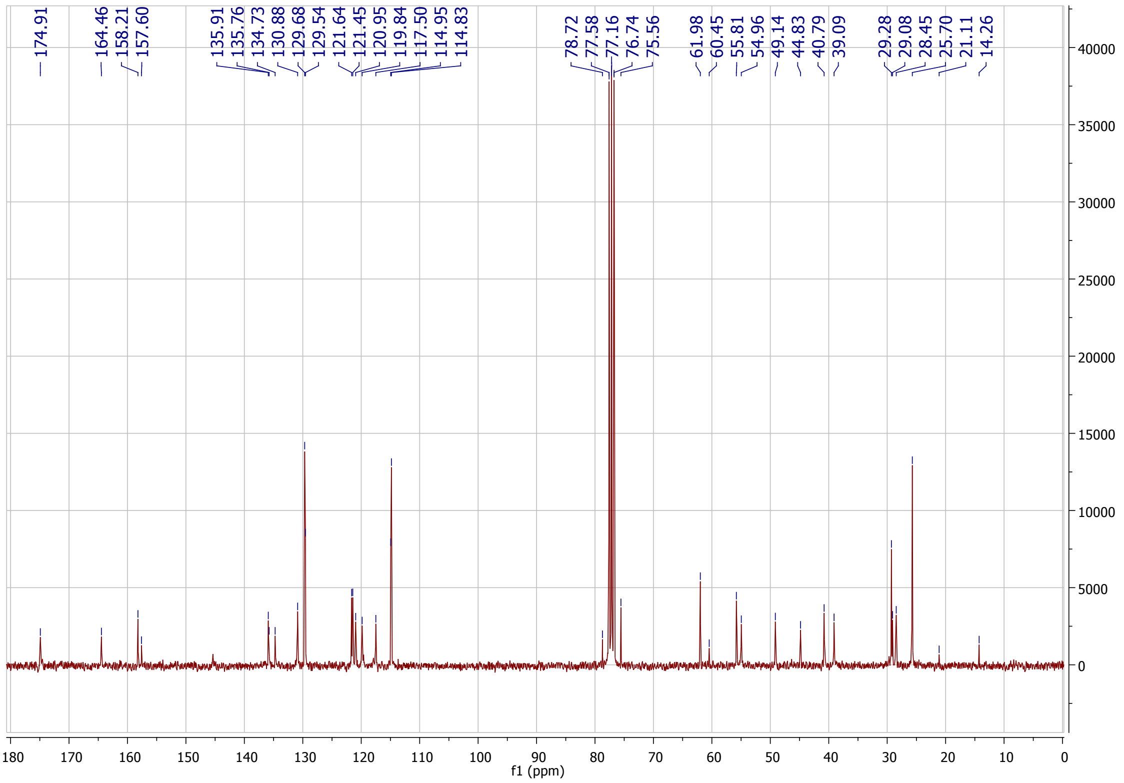

Figure S16 - ^{13}C NMR (75 MHz, $CDCl_3$) of **5c**.

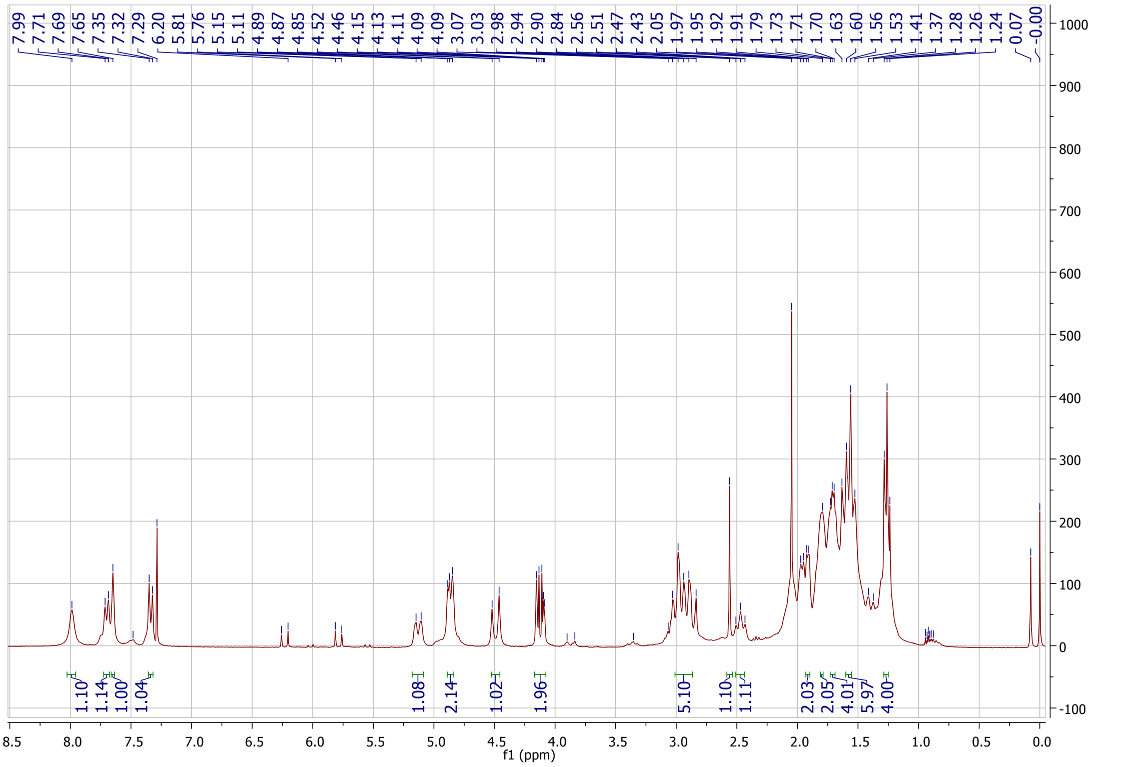

Figure S17 - ^{1}H NMR (300 MHz, $CDCl_3$) of **5d**.

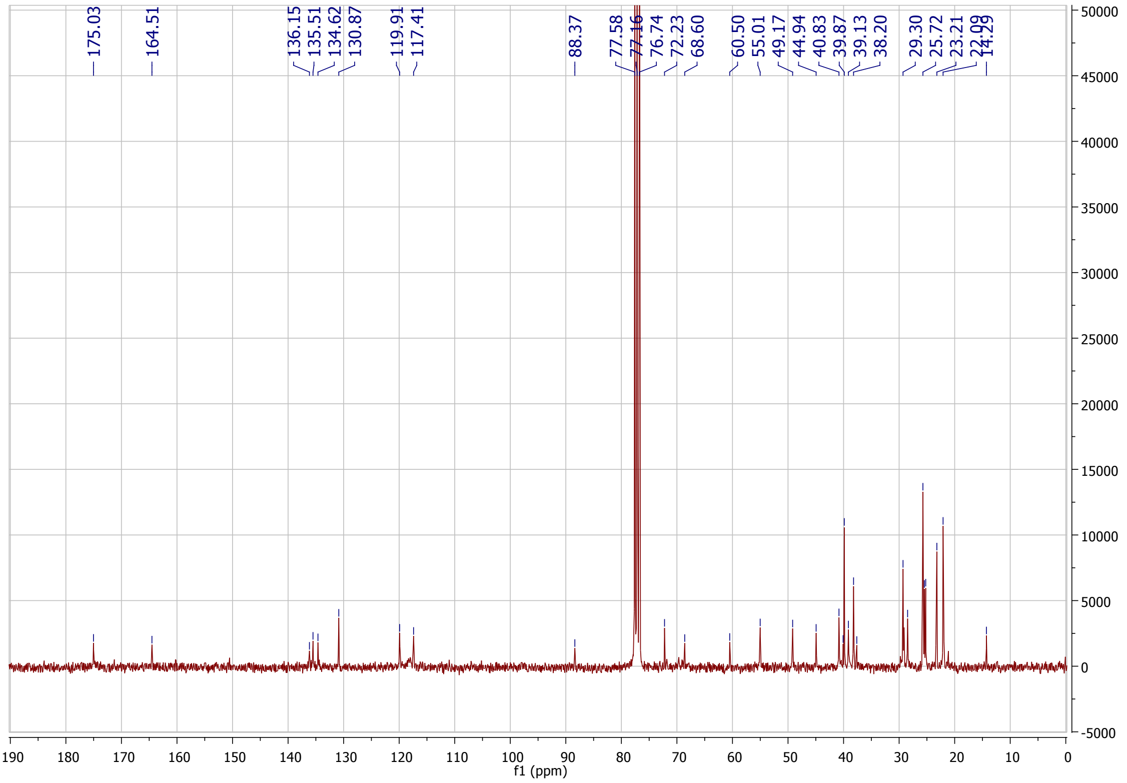

Figure S18 - ^{13}C NMR (75 MHz, $CDCl_3$) of **5d**.

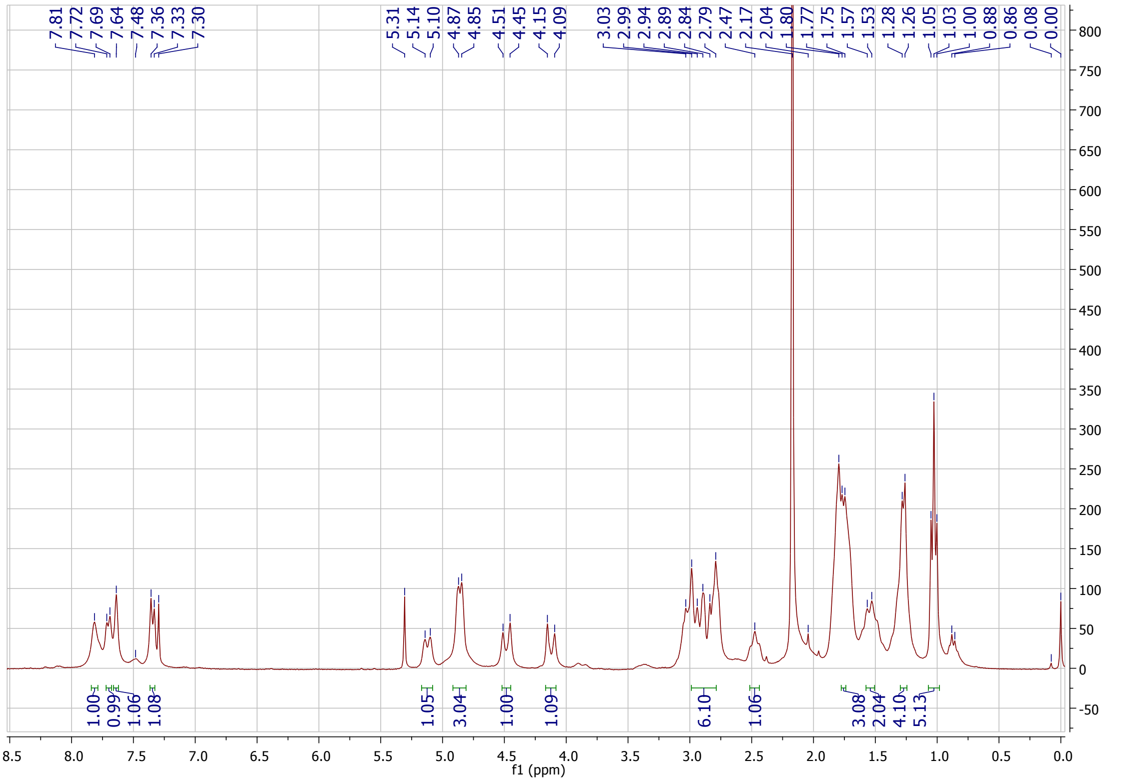

Figure S19 - ^{1}H NMR (300 MHz, $CDCl_3$) of **5e**.

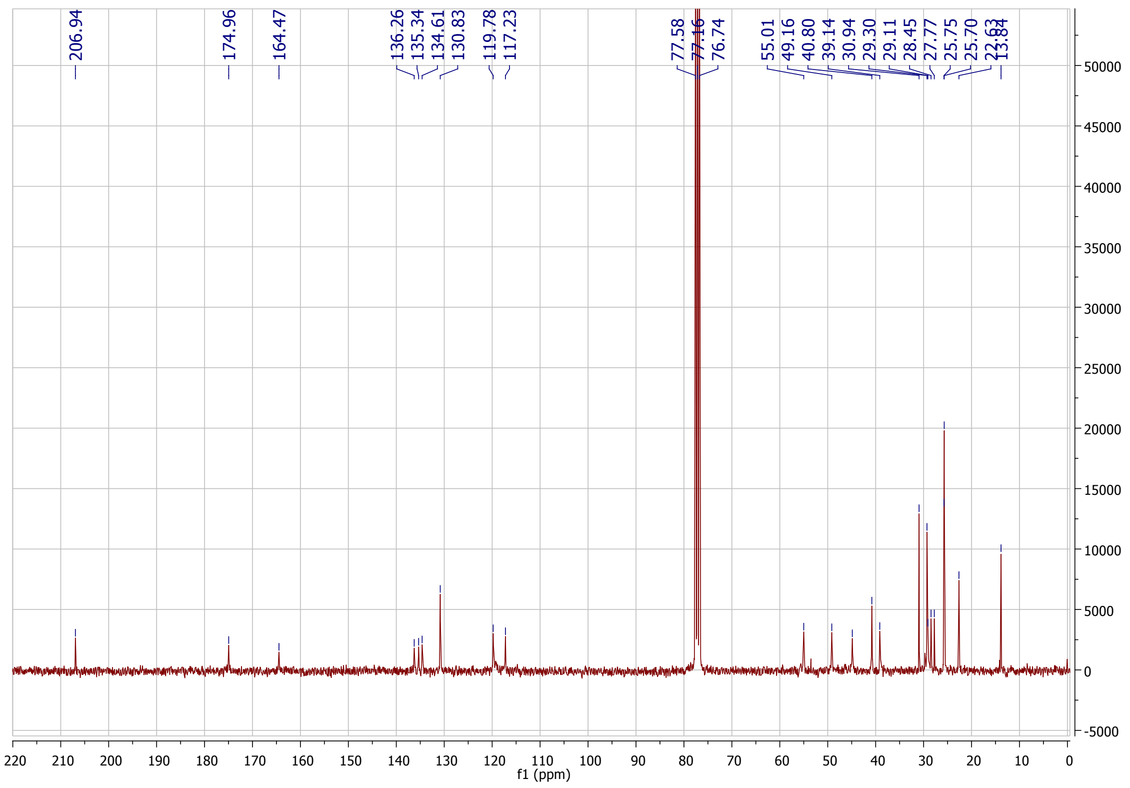

Figure S20 - ^{13}C NMR (75 MHz, $CDCl_3$) of **5e**.

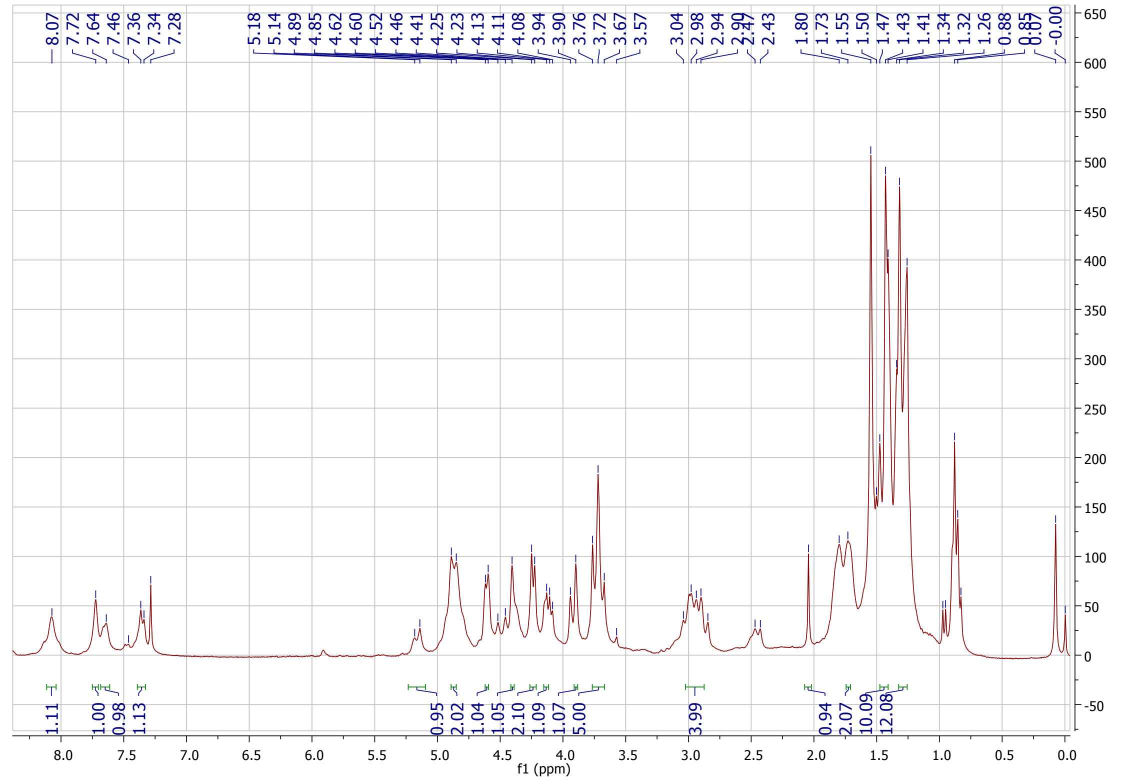

Figure S21 - ^{1}H NMR (300 MHz, $CDCl_3$) of **5f**.

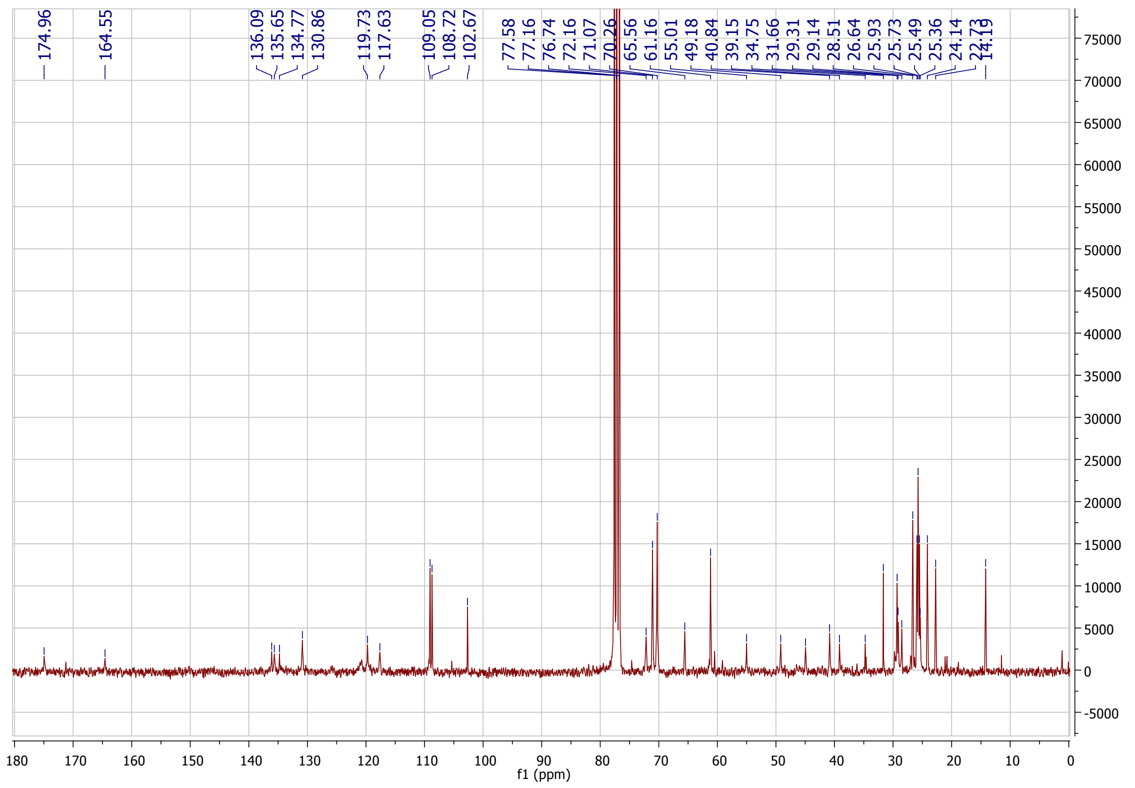

Figure S22 - ^{13}C NMR (75 MHz, $CDCl_3$) of **5f.**

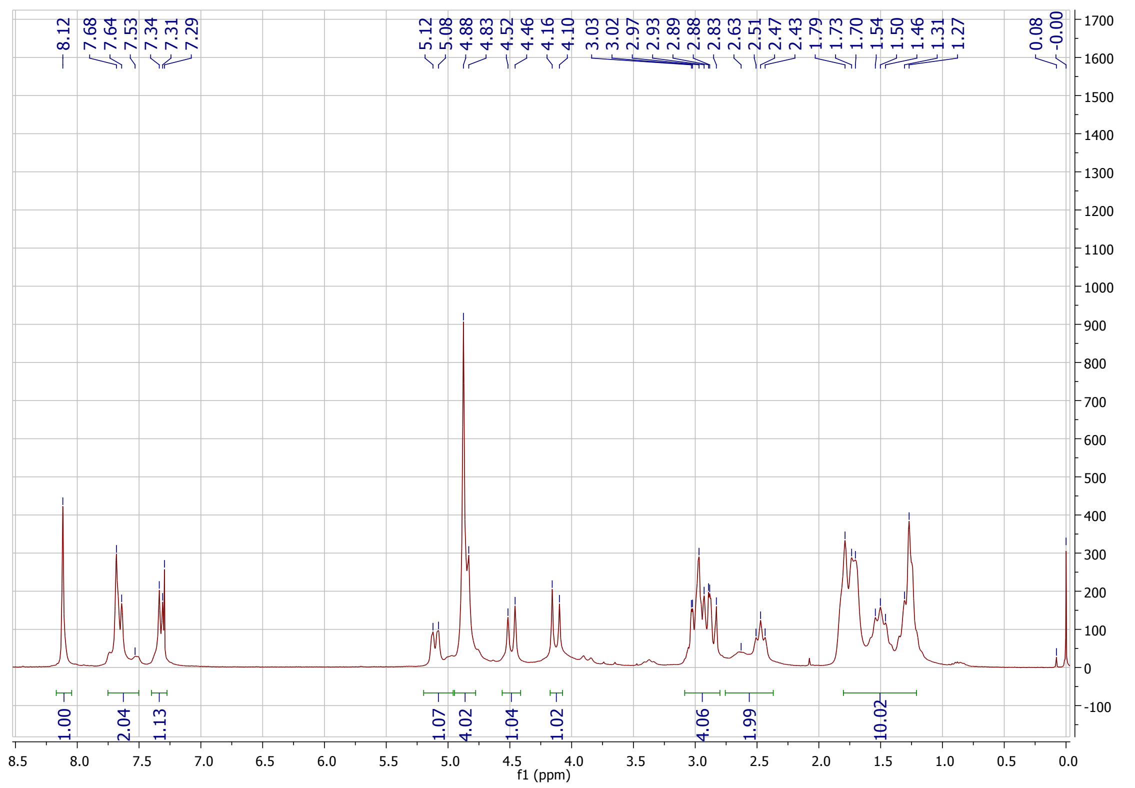

Figure S23 - ^{1}H NMR (300 MHz, $CDCl_3$) of **5g**.

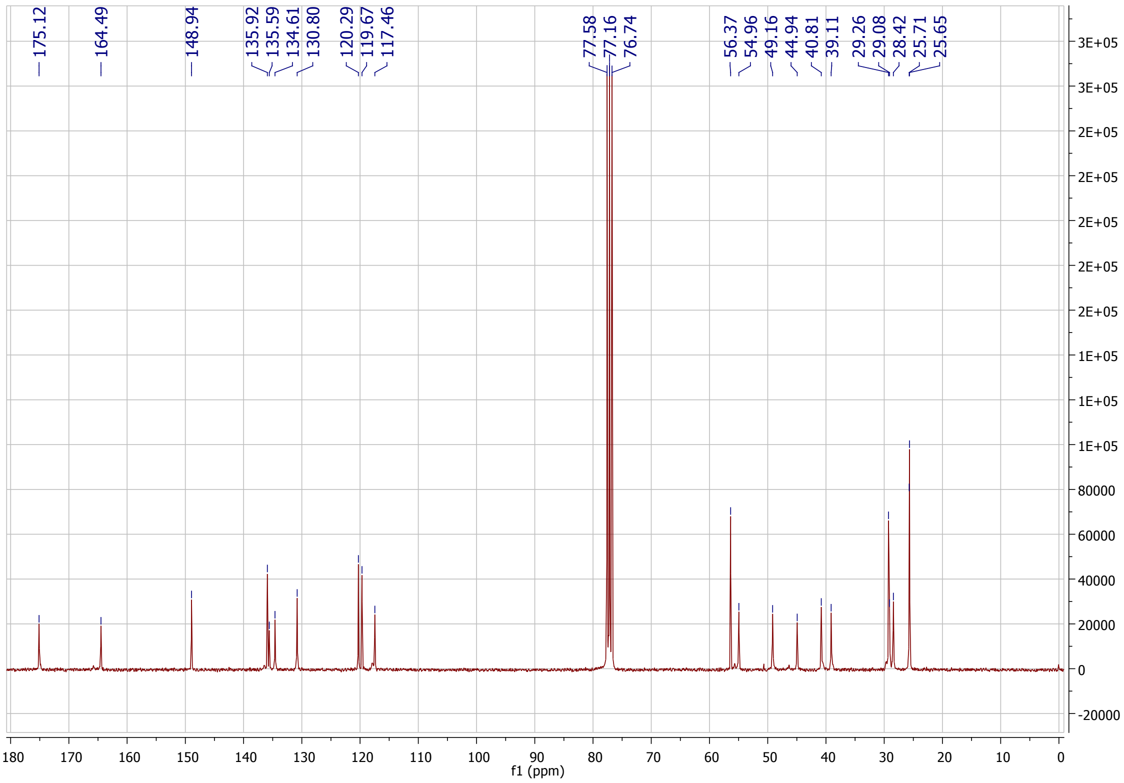

Figure S24 - ^{13}C NMR (75 MHz, $CDCl_3$) of **5g**.

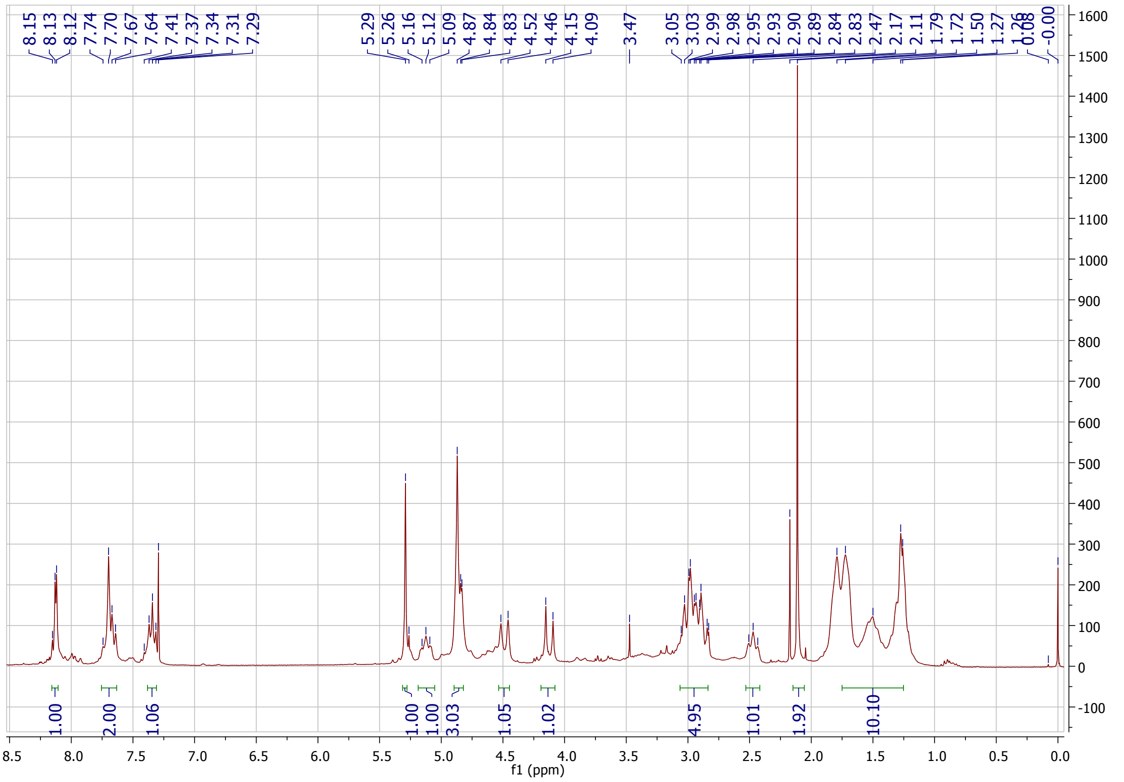

Figure S25 - ^{1}H NMR (300 MHz, $CDCl_3$) of **5h**.

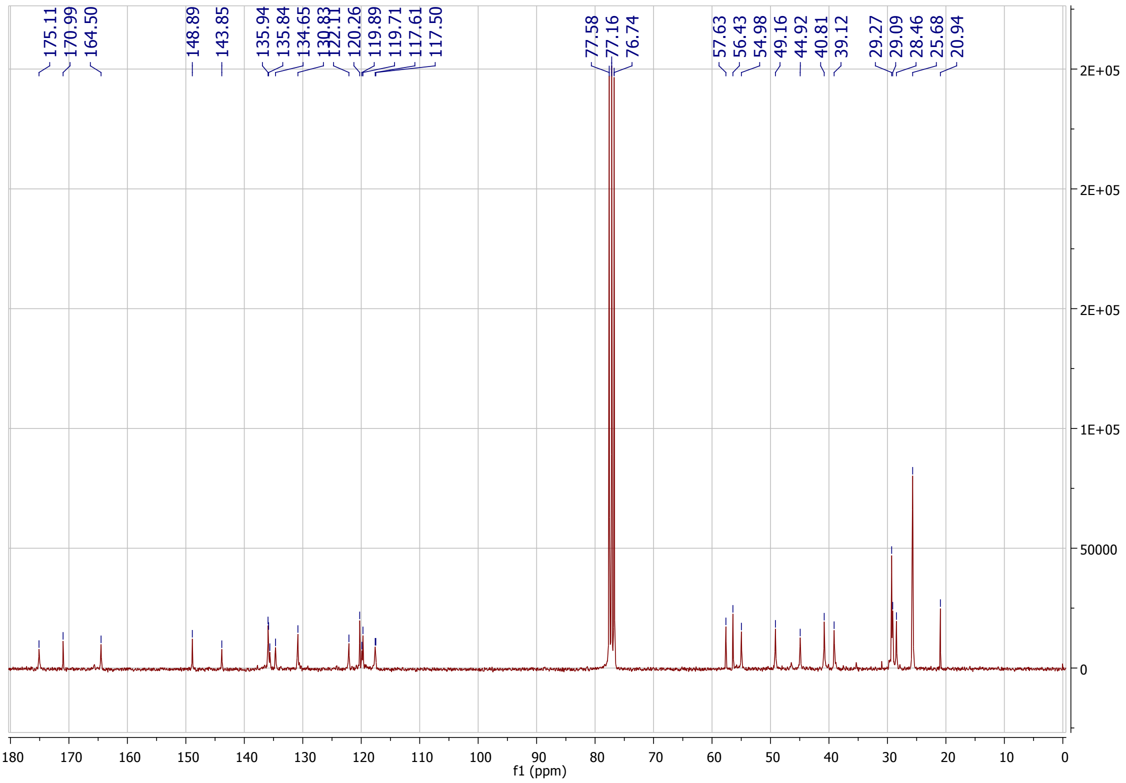

Figure S26 - ^{13}C NMR (75 MHz, $CDCl_3$) of **5h**.

Biology

Methods' details

XTT juvenile viability assay

Initially, a volume of a suspension carrying about 20-40 worms was added to each well of 96-well plates. Then, the volume of each well was adjusted to 200 µL by the addition of the M199 medium and the plates were incubated in 5% CO_2 overnight at 37 °C until the following day. Next, 10 µL of 0.1 % DMSO (negative viability control) or 100 µM of PZQ or its analogues were added to each well, followed by 40 µL of a XTT assay mixture (1.0 mg/mL XTT and 0.383 mg/mL phenazine methasulfate in M199 medium). Positive viability controls, which also received 0.1% DMSO, were prepared using worms heated for 15 minutes at 60 °C prior to plating. Immediately after this step (t_0) the plates was read at 450 nm in a Flexstation 3 Multi-mode microplate reader (Molecular Devices). After 48 hours of incubation a second reading was performed (t_1). The final reading (**A**) was calculated by subtracting the absorbance registered in t_0 from that of t_1.

The residual viability resulting from the action of each compound was determined by comparison, considering 0% residual viability the average of the positive controls (P) for the assay plate and 100% residual viability the average of the negative controls (N) for the same assay plate, according to the following formula: % Residual viability = ((A-P)/(N-P))*100.

Microscopy analysis of juvenile morphology and motility

After isolation, juvenile worms were resuspended in serum-free DMEM medium and transferred to 384 wells plates (1-15 worms per well, over up to 20 wells). The plates were then kept in an incubator at 37 °C with 5% CO_2 overnight. On the following day, 0.02 % DMSO (negative control) or 10 µM PZQ or PZQ analogues were added to the wells. After 72 hours of incubation, 4× bright-field time-lapse images (100 images, 300 ms intervals) were acquired from each well using an ImageXpress Micro Confocal high-content microscope (Molecular Devices) and compiled into a video for visualization.

A visual assigning of a viability score (0-3) was given to each worm based on a combination of both morphological and motility characteristics. For morphological assessment, parasites were scored as 1 if no significant damage was identified, and as 0 if apparent cellular damage was observed. Mobility scoring was based on parasite movement observed in a 30-second video: a score of 0 was assigned when no movement was detected; 1 when movement was observed in only one extremity; 2 when both extremities moved; and 3 when both extremities moved along with a change in the overall conformational arrangement of the worm. The final viability score was calculated by multiplying the individual scores from the morphological and motility assessments. Therefore, parasites with a final viability score of 3 were considered healthy, while lower scores were given to those with progressive damage degrees.

Adult worm ex-vivo high-content assay

Male and female worms were placed in 96-well microplates (one worm per well) and incubated with DMSO (negative control), PZQ (positive control), or test compounds at 0.01-10 µM for up to 72 hours. Brightfield timelapse images (100 images, 0.3-second intervals) were captured from each well before, immediately after, and every 24 hours following compound addition using an ImageXpress Micro Confocal High Content System equipped with a 2x objective. Image analysis was performed through a custom pipeline in CellProfiler software (v. 4.2.1, Windows OS).[57] During analysis, the area of each worm and its displacement between consecutive image captures (i.e., images taken at successive time points) were measured using the parameters "Area" and "False Negative Rate" from the MeasureObjectSizeShape and MeasureObjectOverlap modules, respectively. These measurements were then modeled by a multilevel generalized linear statistical model. The effects of the experimental treatments on parasite motility were estimated with the integrated nested Laplace approximation (INLA) method, available in the INLA package of R (https://www.r-inla.org). The half-maximal inhibitory concentration (EC_{50}) of each compound was calculated based on the worms' estimated motility using the nonparametric 4-parameter logistic equation in GraphPad Prism version 9.0.0 for Windows (GraphPad Software, Boston, MA, USA; www.graphpad.com).

Cytotoxicity assay

The cells were seeded into 96-well microplates at 5 x 10^3 (HepG2) or 5 x 10^4 (WSS-1) per well and kept overnight in an incubator (37°C, 5% CO_2, humid atmosphere) to allow for adherence. In the following day, the cells were incubated for up to 48 hours with PZQ and its analogues, either at a fixed concentration (10 µM) or across a range of concentrations (0.0001–300 µM), or with DMSO as a negative control. Two hours before the end of the experiment (t_0), a resazurin solution (final concentration: 0.01 mg/mL) was added to each well and the first fluorescence reading (λ_{ex} = 530 nm, λ_{ex} = 580 nm) was carried out in a FlexStation 3 Multi-mode microplate reader. At the end of the incubation (t_1), a second reading was performed. The fluorescence attributed solely to resorufin was measured by subtracting the fluorescence readings at t_0 from those at t_1. The results were expressed as percentual cell viability considering negative control as 100 %. The half-maximal cytotoxic concentration (CC_{50}) of each compound was determined as described for EC_{50} in adult worm assay, with the bottom parameter of the logistic equation constrained to 0.

Ca2+ reporter assay

HEK293 cells were transiently transfected with either *Sm*.$TRPM_{PZQ}$, *Sm*.$TRPM_{PZQ}$ mutants, *Fh*.$TRPM_{PZQ}$, *Eg.*$TRPM_{PZQ}$ or *Sm*.$TRPM_{MCLZ}$. Untransfected HEK293 cells were used as a control. Cells were seeded into black-walled, clear-bottom, poly-D-lysine–coated 384-well plates (Greiner Bio-One, Germany) at a density of 20,000 cells per well in DMEM supplemented with 10% fetal bovine serum (FBS). Plates were incubated for 24 hours at 37 °C in a humidified atmosphere containing 5% CO_2. Following incubation, the growth medium was removed and replaced with 20 µL of Fluo-4 NW calcium indicator dye (Invitrogen), reconstituted in assay

buffer containing 2.5 mM probenecid in HBSS (with Ca^{2+} and Mg^{2+}), 20 mM HEPES, 0.3% BSA (Sigma, A7030), and 0.03% L-ascorbic acid (Sigma). Cells were incubated for 40 minutes at 37 °C and subsequently for 20 minutes at room temperature. A total of seven PZQ derivatives were tested, using racemic PZQ included as a positive control on each assay plate. Drug dilutions were prepared in assay buffer without dye and probenecid and dispensed into separate flat-bottom 384-well plates. Calcium flux measurements were performed at room temperature using a FLIPR^{TETRA} instrument (Molecular Devices). Baseline fluorescence was recorded for 20 seconds, followed by the addition of 5 µL of each drug solution, after which fluorescence was measured continuously for 250 seconds.

Complementary data

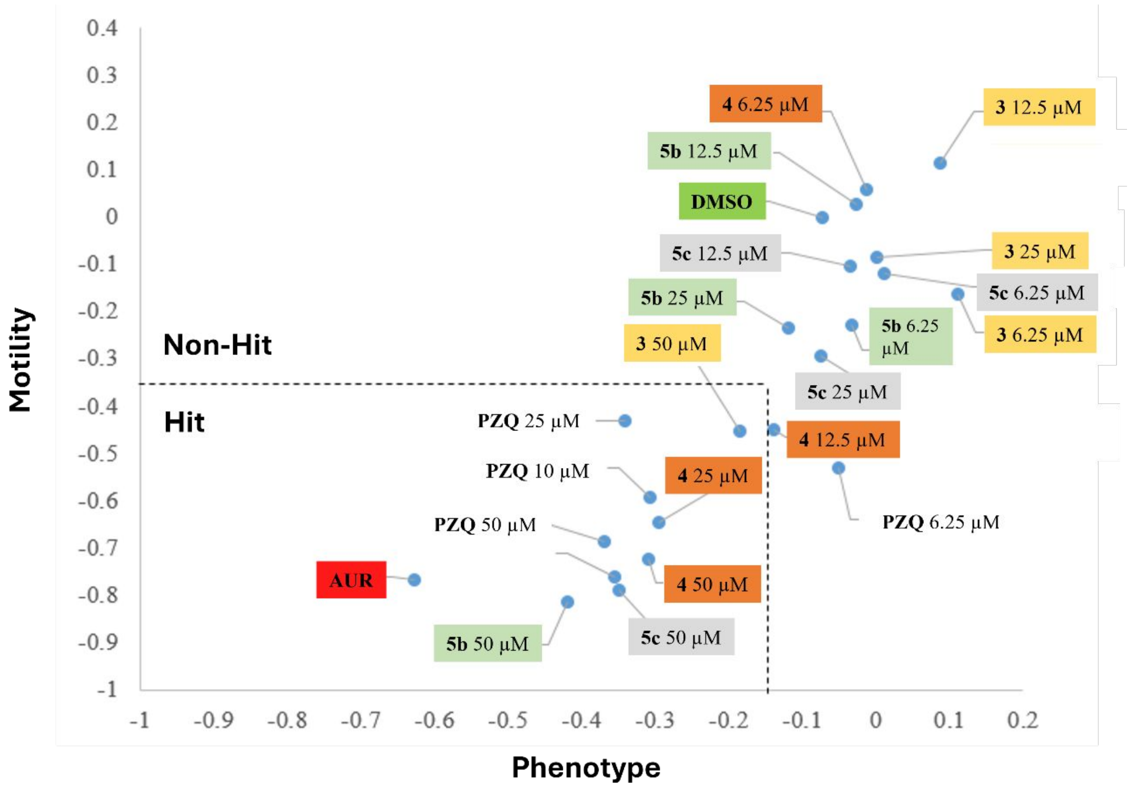

Figure S27- Titration (6.25 - 50 µM) of PZQ and its derivatives against *S. mansoni* schistosomula after 72h of incubation using the Roboworm high-content screening assay. Auranofin (AUR) was included at 10 µM as positive control. DMSO was included as a negative control. Compounds were considered hits if both their motility and phenotype scores were lower than -0.35 and -0.15, respectively (region delimited by the dashed lines).

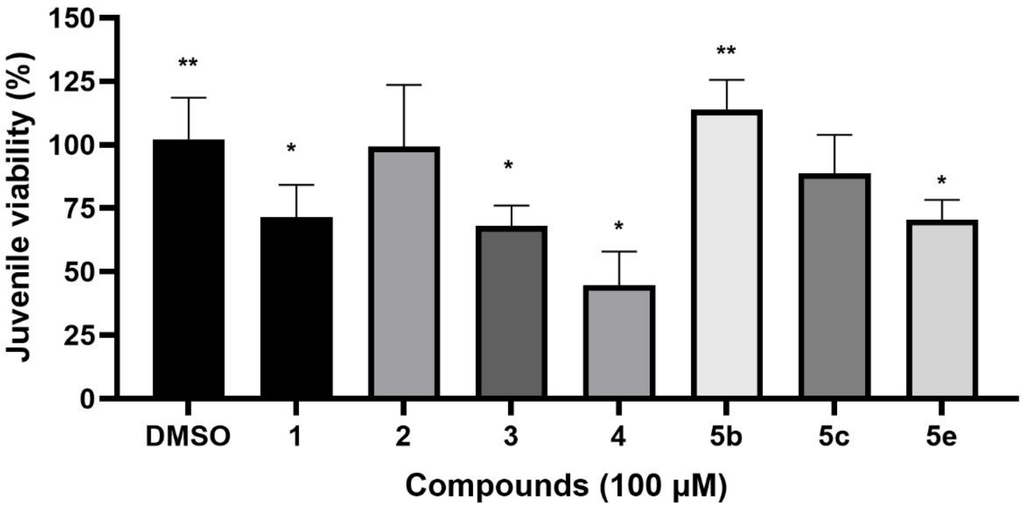

Figure S28- Effect of PZQ analogues on the viability of juvenile schistosomes after 48h incubation using the XTT assay. The bars represent the mean ± standard deviation. *p<0.05 compared to the DMSO-treated group. **p<0.05 compared to the PZQ-treated group. Statistical analysis was carried out in GraphPad Prism using One-way ANOVA followed by Dunnett's multiple comparison test.

Table S1- Viability score distribution of juvenile worms in phenotypic microscopy assay.

Compound	Viability score				Total
	0	1	2	3	
**DMSO**	23	5	27	65	120
**PZQ (1)**	56	12	27	14	109
**2**	70	3	7	74	154
**3**	38	2	14	38	92
**4**	48	4	25	12	89
**5b**	21	1	3	7	32
**5c**	19	1	4	72	96
**5e**	61	7	34	6	108

Table S2- Comparison of phenotypic profiles between DMSO- and PZQ analogue–treated juvenile schistosomes using Pearson's Chi-squared test.

Compound	p value	Statistical Difference (p < 0.05) vs. DMSO
**1**	1.20E-10	Yes
**2**	3.40E-07	Yes
**3**	5.34E-03	Yes
**4**	4.44E-09	Yes
**5b**	6.90E-06	Yes
**5c**	4.35E-04	Yes

**5e**	1.96E-14	Yes

Table S3 - Comparison of phenotypic profiles between PZQ- and PZQ analogue–treated juvenile schistosomes using Pearson's Chi-squared test.

Compound	p value	Statistical Difference ($p < 0.05$) vs. PZQ
**DMSO**	1.20E-10	Yes
**2**	1.44E-11	Yes
**3**	1.94E-05	Yes
**4**	4.15E-01	No
**5b**	8.22E-02	No
**5c**	6.16E-18	Yes
**5e**	1.37E-01	No

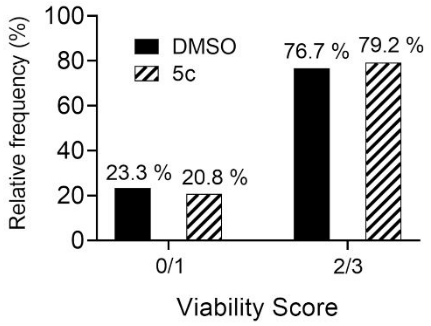

Figure S29 – Grouped analysis combining relative frequencies of scores 0/1 and 2/3 of juvenile schistosomes incubated with 10 µM **5c** or 0.02 % DMSO for 72 hours.

Table S3 – Half-maximal motility inhibition (EC_{50}) for male and female worms treated with PZQ and its analogues.

Cmp / Time	Male worm motility inhibition (EC_{50}; µM)				Female worm motility inhibition (EC_{50}; µM)			
	0 h*	24 h	48 h	72 h	0 h*	24 h	48 h	72 h
PZQ (**1**)	0.3/0.27 (0.28)	0.22/0.28 (0.25)	0.24/0.27 (0.25)	0.16/0.17 (0.16)	Not determined	Not determined	Not determined	Not determined
**2**	10.03/11.54 (10.7)	10.33/19.75 (14.3)	11.63/21.66 (15.9)	11.86/19.93 (15.4)	27.72	28.79	29.1	30
**3**	Undefined	3.44/8.1/5.61 (5.4)	3.94/4.56/5.03 (4.5)	3.8/3.29/3.58 (3.5)	8.36	9.04/10.15 (9.6)	9.36/10.54 (9.9)	8.77/14.28 (11.2)
**4**	2.83/1.93 (2.3)	2.56/1.42/1.74 (1.8)	2.37/1.1/1.71 (1.6)	1.22/1.0/1.72 (1.3)	14.79/3.02 (6.7)	3.15	26.76/3.34 (9.4)	3.48
**5b**	31.59/33.79 (32.7)	50.38/43.78 (47.0)	54.21/44.62 (49.2)	44.67/36.65 (40.5)	100.8	96.25	95	89.11
**5c**	No inhibition	Partial fit (>100)	Partial fit (>100)	Partial fit (>100)	Not determined	Not determined	Not determined	Not determined
**5e**	2.5/2.68 (2.6)	2.29/2.32/2.84 (2.5)	1.70/2.12/1.99 (1.9)	1.64/1.99/1.57 (1.7)	2.98/2.78/5.33 (3.5)	3.70/3.14/3.9 (3.6)	3.14/4.77 (3.9)	2.94/3.3 (3.1)

*Immediately after addition of compounds. The geometric mean of the EC_{50} values from different experiments is shown in parentheses.

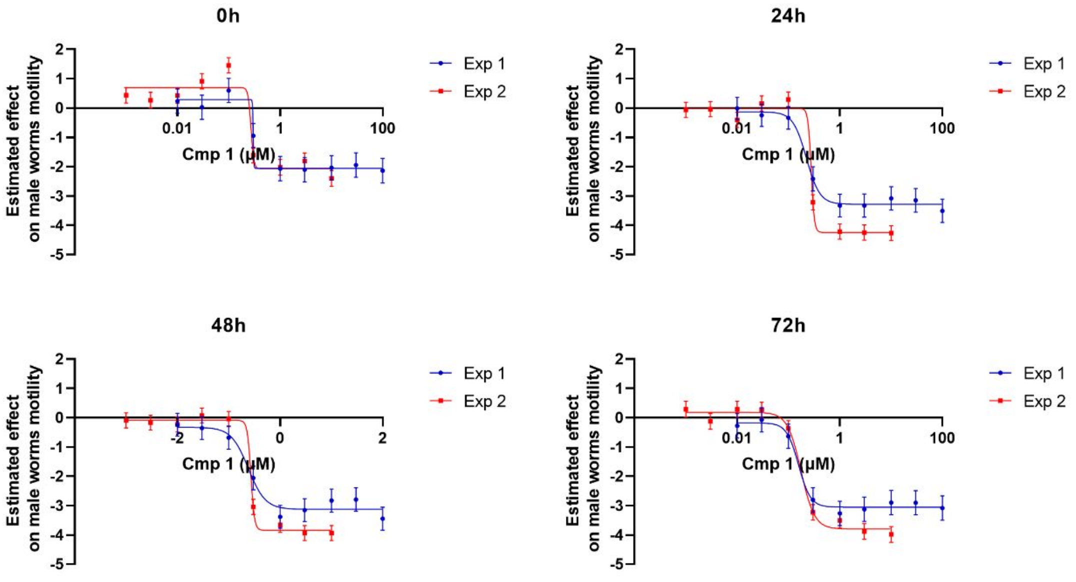

Figure S30 – Dose-response curves of compound **1** (PZQ) in male adult schistosomes.

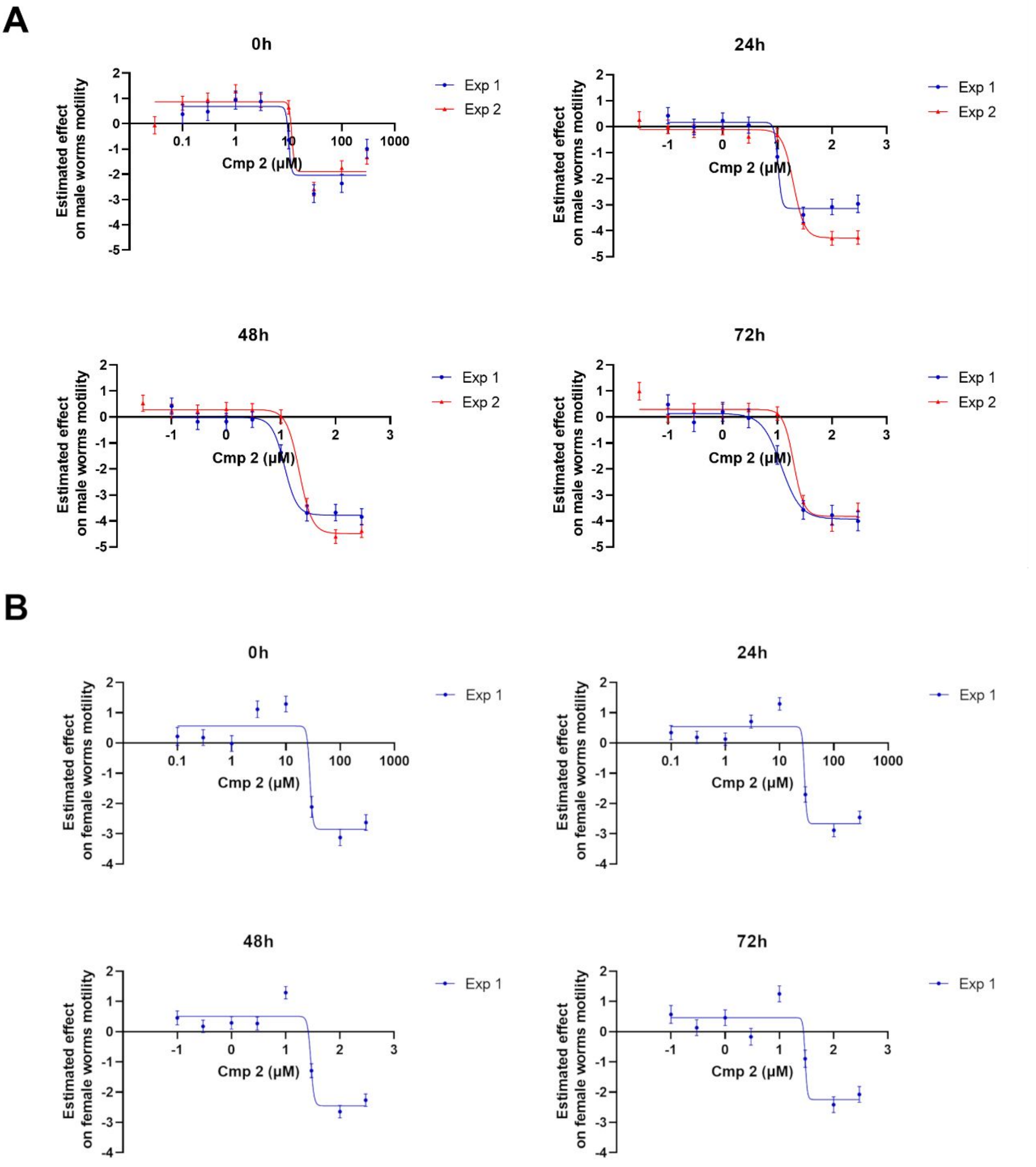

Figure S31 - Dose-response curves of compound **2** in male (A) and female (B) adult schistosomes.

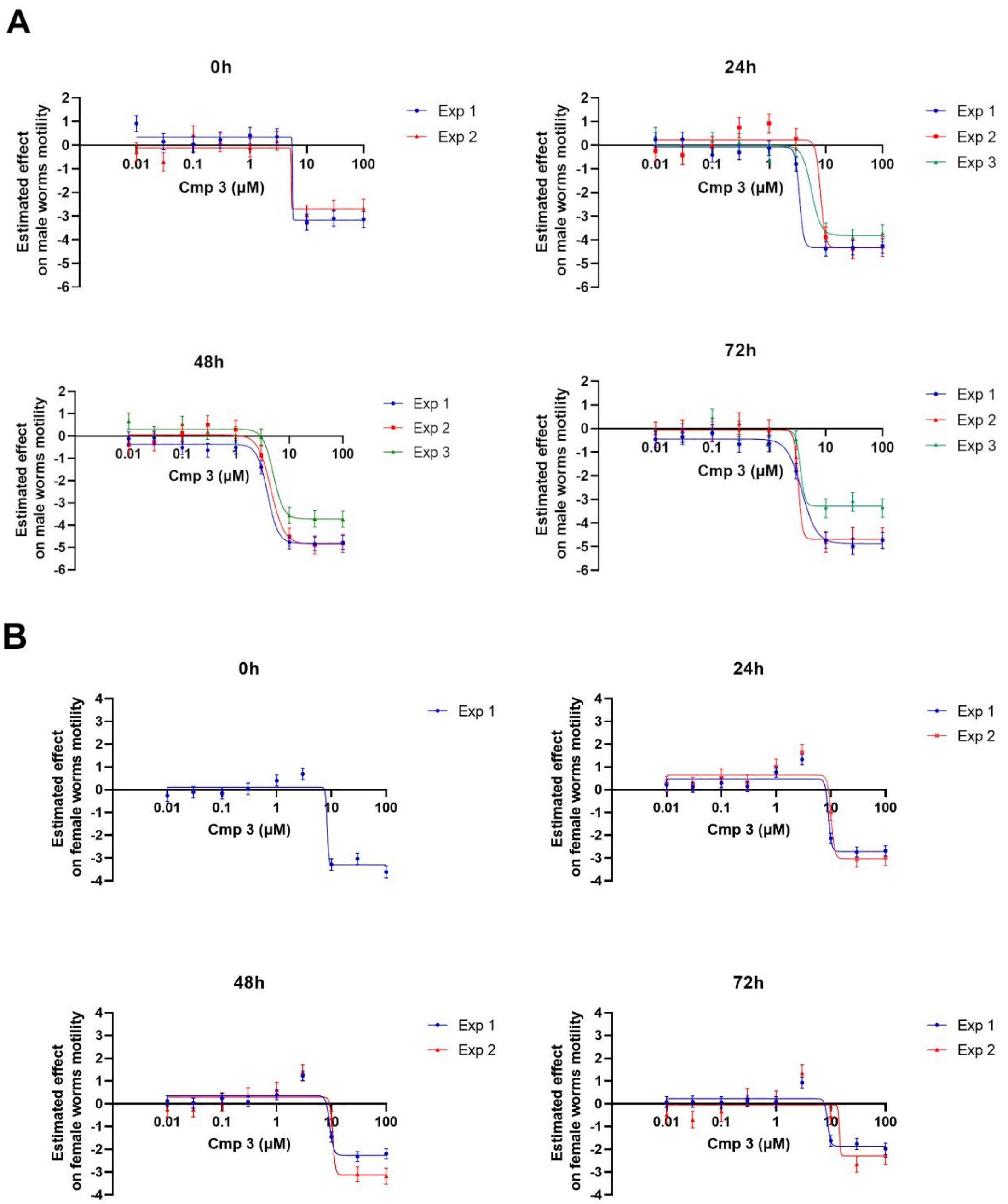

Figure S32 - Dose-response curves of compound **3** in male (A) and female (B) adult schistosomes.

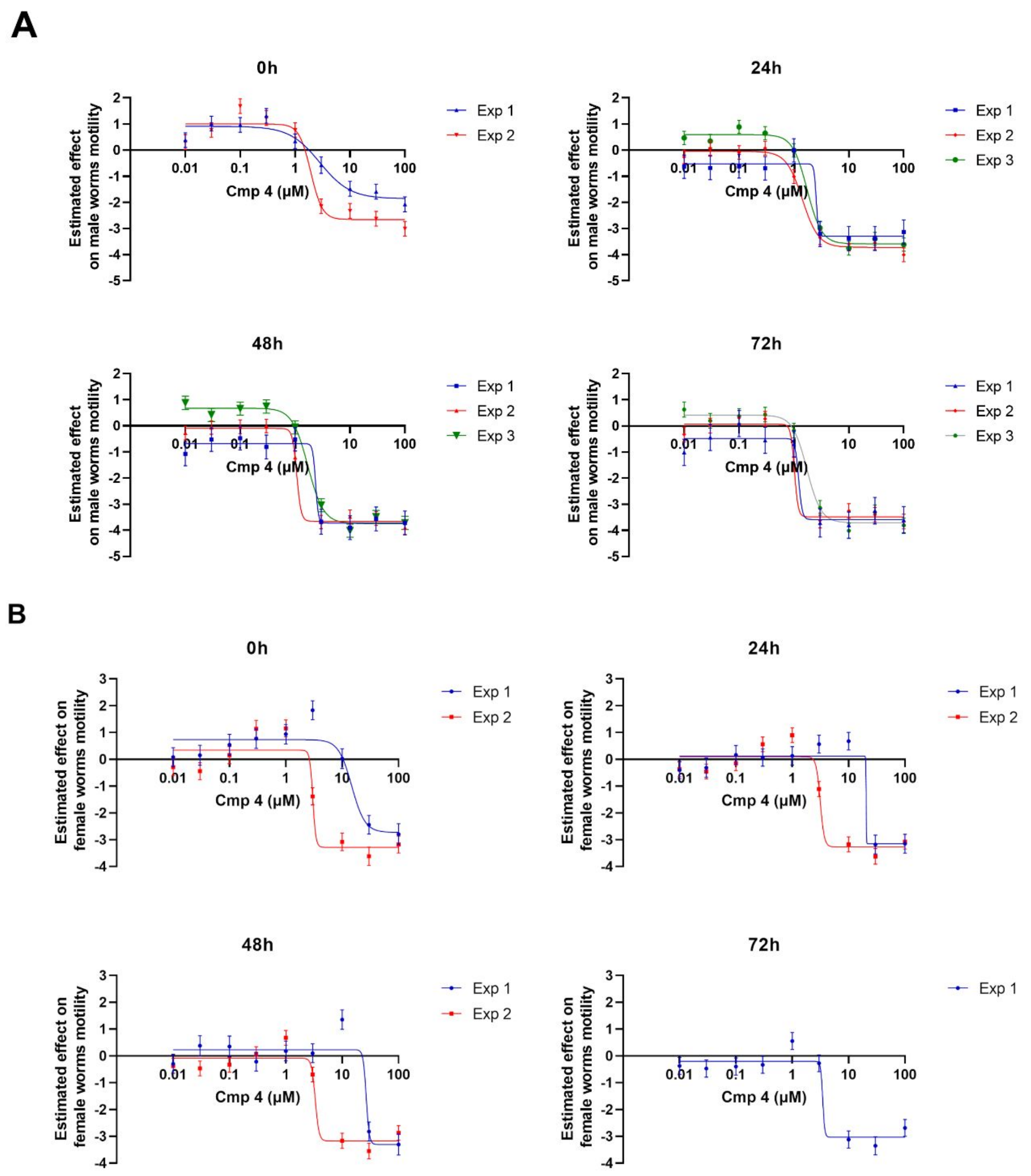

Figure S33 - Dose-response curves of compound **4** in male (A) and female (B) adult schistosomes.

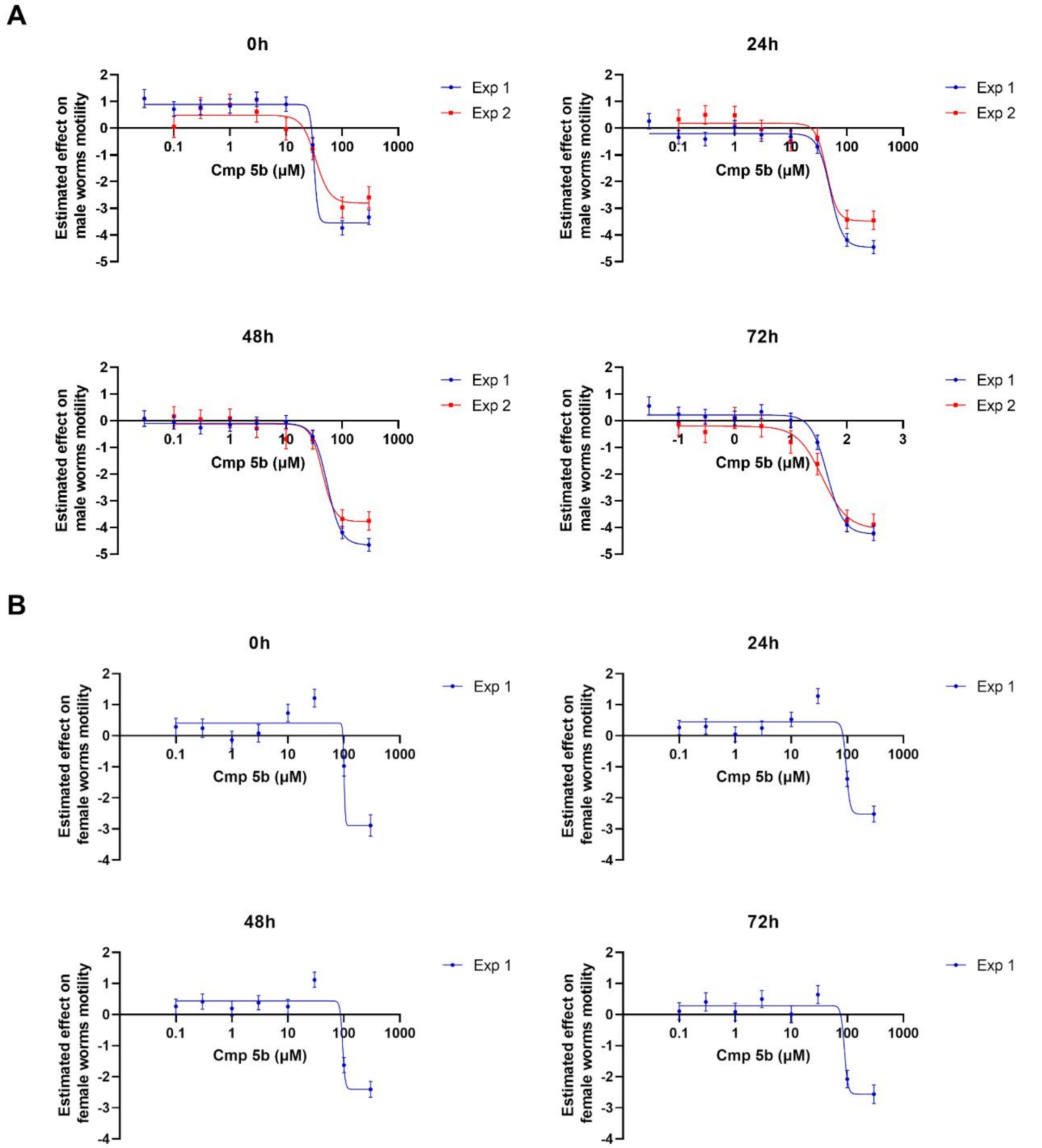

Figure S34 - Dose-response curves of compound **5b** in male (A) and female (B) adult schistosomes.

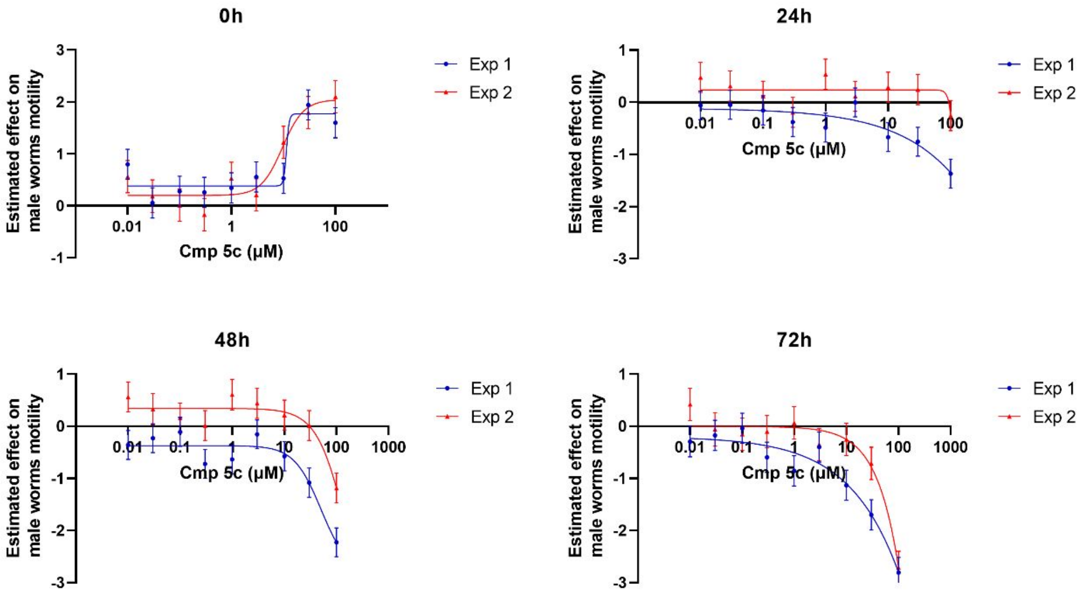

Figure S35 - Dose-response curves of compound **5c** in male adult schistosomes.

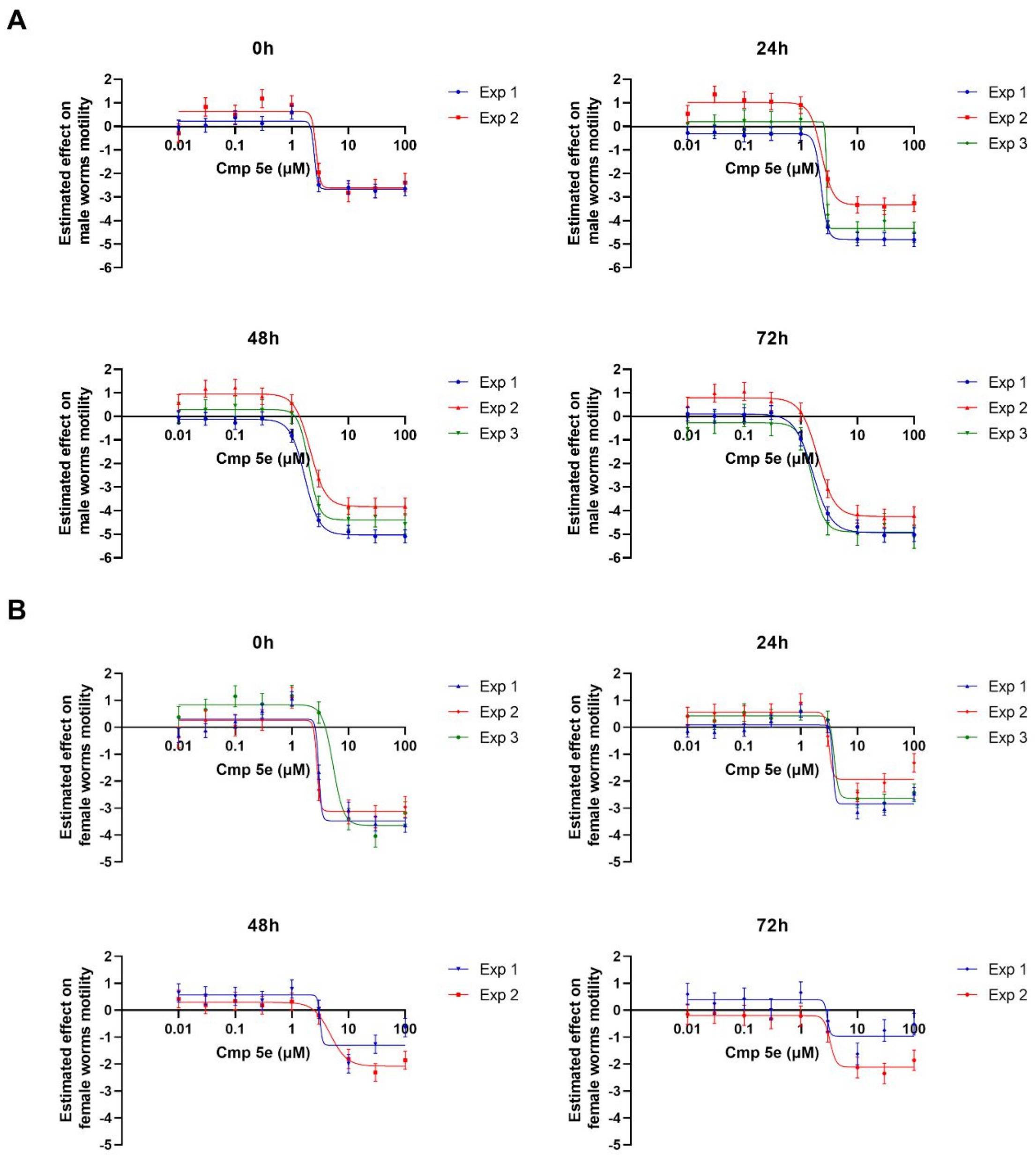

Figure S36 - Dose-response curves of compound **5e** in male (A) and female (B) adult schistosomes.

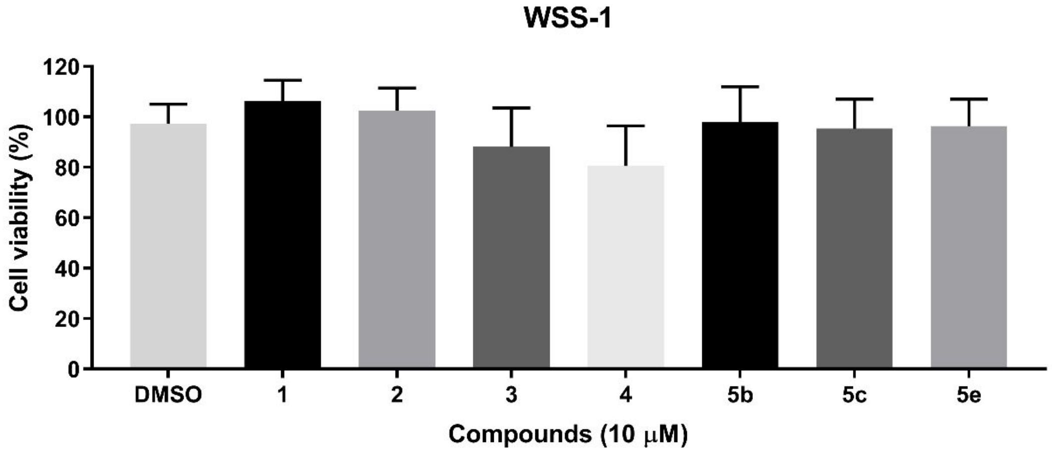

Figure S37 – WSS-1 cells viability after 48h of incubation with PZQ analogues. The bars represent the mean ± standard deviation. *p<0.05 compared to the DMSO-treated group. Statistical analysis was carried out in GraphPad Prism using One-way ANOVA followed by Dunnett's multiple comparison test.

Table S4 – Half-maximal cytotoxic concentration (CC_{50}), half-maximal motility inhibition (EC_{50}) and selectivity index for male and female worms treated with PZQ and its analogues.

Compound	HepG2 cytotoxicity (CC_{50}; μM)	Worm motility inhibition (EC_{50}; μM)		Selectivity Index (CC_{50}/EC_{50})	
		Male	Female	Male	Female
PZQ	> 300	0.24/0.27 (0.25)	Not determined	> 1200	Not determined
2	354.5/345.6/406.1 (367.8)	11.63/21.66 (15.9)	29.1	23.1	12.7
3	> 300	3.94/4.56 / 5.03 (4.5)	9.36/10.54 (9.9)	>66.7	>30.3
4	154.7/154.8/159.9 (156.4)	2.37/1.1/1.71 (1.6)	26.76/3.34 (9.4)	97.7	16.6
5b	28.71/27.44/29.37 (28.5)	54.21/44.62 (49.2)	95	0.6	0.3
5c	175.9/83.97/40.49 /100.8 (88.1)	Partial fit (>100)	Not determined	< 0.9	Not determined
5e	286.3/301.9 (294.0)	1.70/2.12/1.99 (1.9)	3.14/4.77 (3.9)	154.7	75.4

The geometric mean of the EC_{50} values from different experiments is shown in parentheses. For some compounds, EC_{50} values could not be calculated either because the experiment was not performed (not determined) or the concentrations tested were insufficient to reach the bottom plateau of the dose-response curve (partial fit).

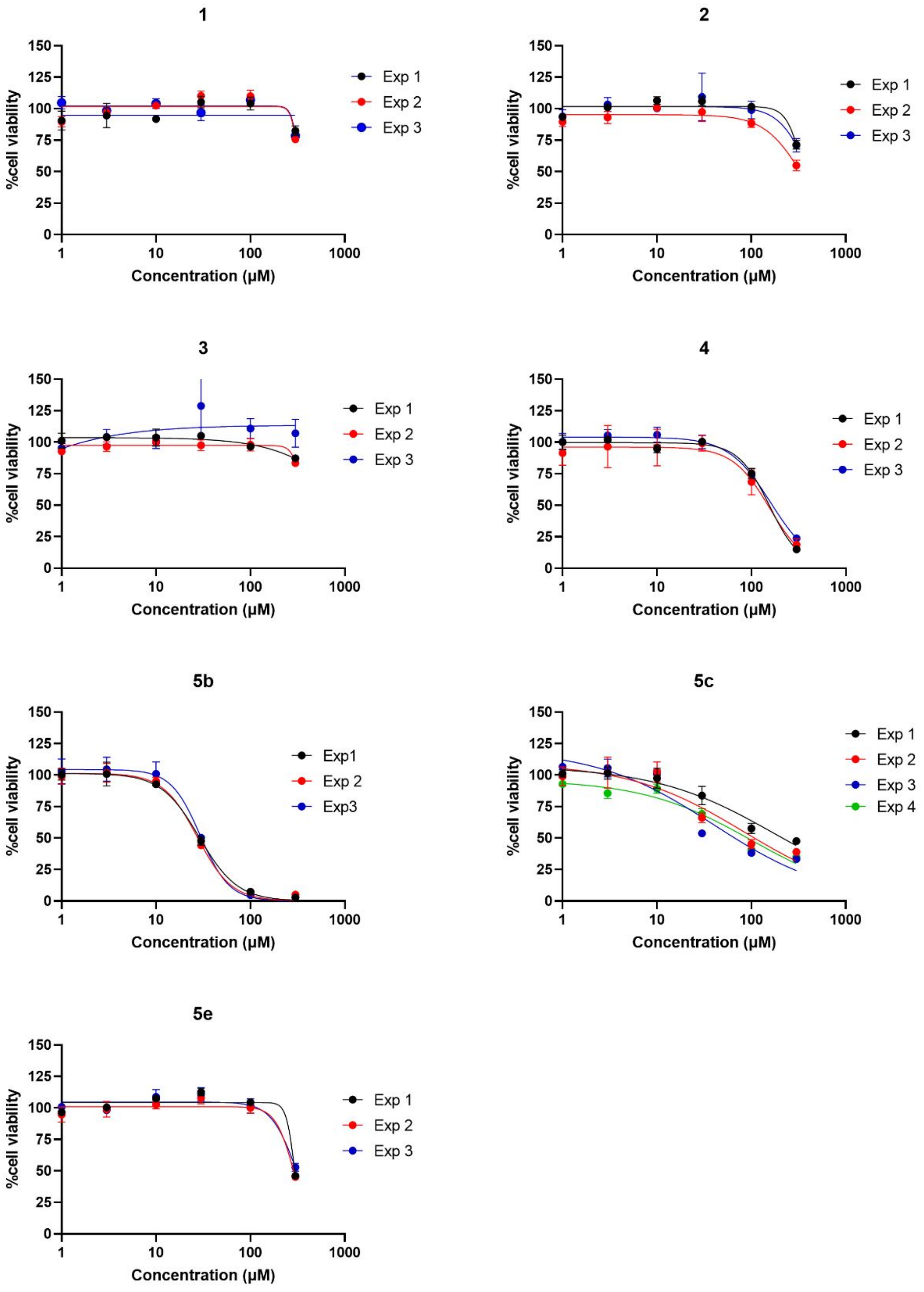

Figure S38 - Dose-response curves of HepG2 cell viability after 48 hours of incubation with PZQ analogues. Data expressed as mean ± standard deviation. For each curve, the bottom parameter of the logistic equation was constrained to 0.

In silico data

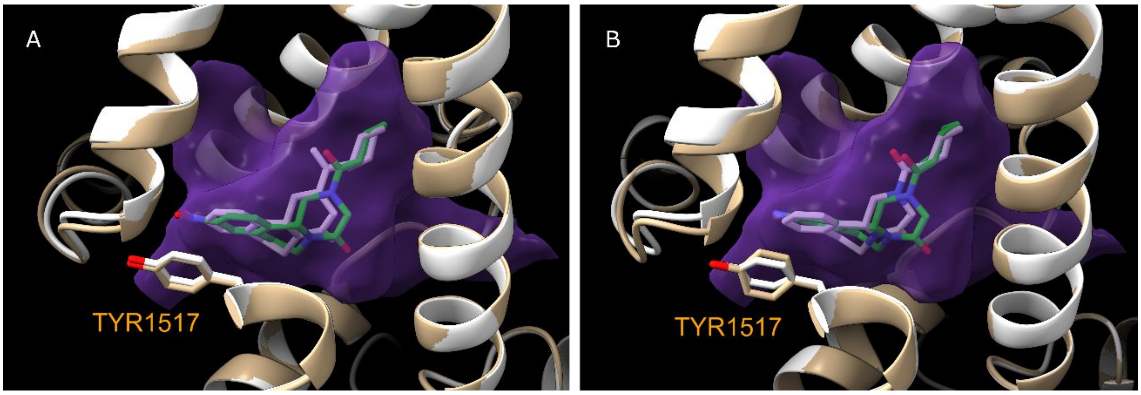

Figure S39 - Comparison of the binding pose of PZQ and its channel-active analogues **2** (A) and **3** (B) within the predicted PZQ binding site of the wild type *Sm*.TRPM$_{PZQ}$. Protein helices were colored white and copper to illustrate the binding poses of PZQ and its analogues, respectively. Compounds are represented as white (PZQ) or green (PZQ derivatives) thick sticks. The cavity surface of PZQ binding site is colored purple.

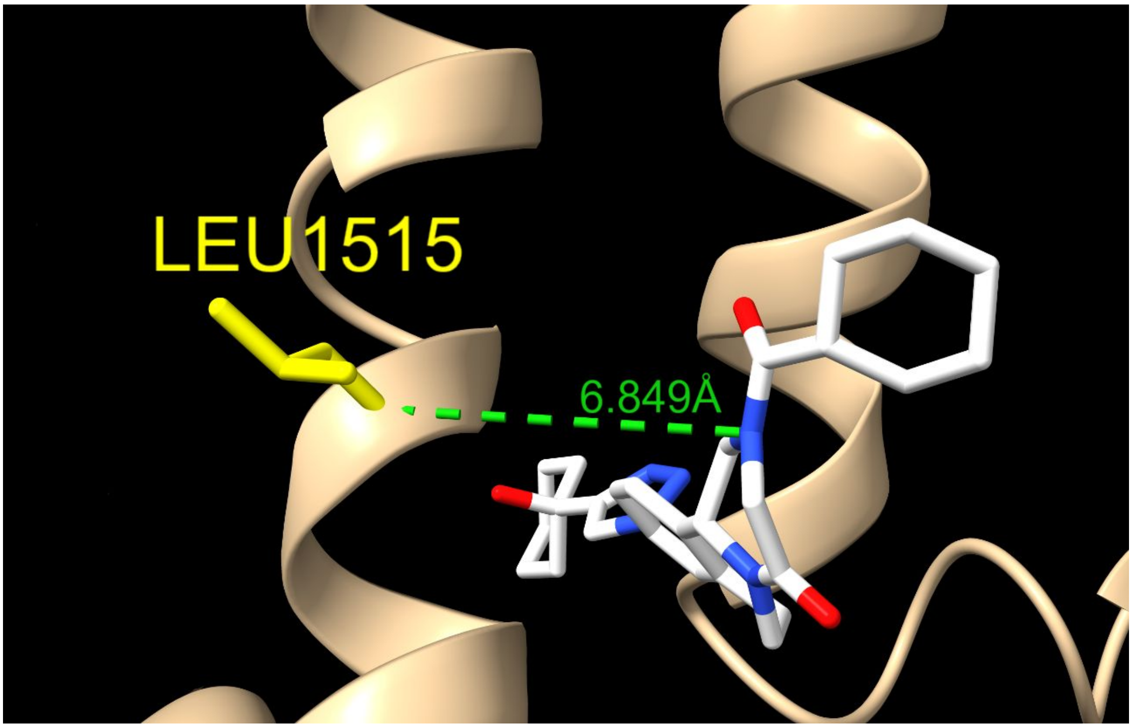

Figure S40 – Predicted binding pose of **5d** in *Sm.*TRPM$_{PZQ}$. Protein helices are shown in copper, while **5d** is represented as a thick white stick. The green dashed line indicates the minimal distance between **5d** and the side chain of leucine 1515 (LEU1515).
